# Performance-based clinical tests of balance and muscle strength used in young seniors: a systematic literature review

**DOI:** 10.1186/s12877-018-1011-0

**Published:** 2019-01-09

**Authors:** Ronny Bergquist, Michaela Weber, Michael Schwenk, Synnøve Ulseth, Jorunn L. Helbostad, Beatrix Vereijken, Kristin Taraldsen

**Affiliations:** 1 Department of Neuromedicine and Movement Science, Norwegian Univerity of Science and Technology, Trondheim, Norway; 2 Network Aging Research (NAR), Heidelberg University, Heidelberg, Germany; 3 Institute of Sports and Sports Sciences, Heidelberg University, Heidelberg, Germany

**Keywords:** Systematic review, Performance-based tests, Measurement properties, Older adults, Balance, Muscle strength

## Abstract

**Background:**

Many balance and strength tests exist that have been designed for older seniors, often aged ≥70 years. To guide strategies for preventing functional decline, valid and reliable tests are needed to detect early signs of functional decline in young seniors. Currently, little is known about which tests are being used in young seniors and their methodological quality. This two-step review aims to 1) identify commonly used tests of balance and strength, and 2) evaluate their measurement properties in young seniors.

**Methods:**

First, a systematic literature search was conducted in MEDLINE to identify primary studies that employed performance-based tests of balance and muscle strength, and which aspects of balance and strength these tests assess in young seniors aged 60–70. Subsequently, for tests used in ≥3 studies, a second search was performed to identify method studies evaluating their measurement properties. The quality of included method studies was evaluated using the Consensus-based Standards for selection of health Measurement Instruments (COSMIN) checklist.

**Results:**

Of 3454 articles identified, 295 met the inclusion criteria. For the first objective, 69 balance and 51 muscle strength tests were identified, with variations in administration mode and outcome reporting. Twenty-six balance tests and 15 muscle strength tests were used in ≥3 studies, with proactive balance tests and functional muscle power tests used most often. For the second objective, the search revealed 1880 method studies, of which nine studies (using 5 balance tests and 1 strength test) were included for quality assessment. The Timed Up and Go test was evaluated the most (4 studies), while the Community Balance and Mobility (CBM) scale was the second most assessed test (3 studies). For strength, one study assessed the reliability of the Five times sit-to-stand.

**Conclusion:**

Commonly used balance and muscle strength tests in young seniors vary greatly with regards to administration mode and outcome reporting. Few studies have evaluated measurement properties of these tests when used in young seniors. There is a need for standardisation of existing tests to improve their informative value and comparability. For measuring balance, the CBM is a new and promising tool to detect even small balance deficits in balance in young seniors.

**Electronic supplementary material:**

The online version of this article (10.1186/s12877-018-1011-0) contains supplementary material, which is available to authorized users.

## Background

Numerous studies have demonstrated that impairments in balance and decreased muscle strength in lower extremity muscles are important risk factors for early age-related decline in physical function [1–5], falls [3–6], future disabilities [7], hospitalization [5], and death [6–8]. Early declines in balance and muscle strength are already apparent in the third decade of life [9–12], with an accelerated decline occurring from the decade of young seniors aged 60 to 70 years [9, 13–15]. Especially age-related impairments in vision and the vestibular and proprioceptive systems, most obvious from 50 years and older [9, 16, 17], contribute to the acceleration of balance decline. For muscle strength, especially age-related changes in lean muscle mass greatly increase the risk for physical inactivity, mobility deficits, functional limitations and falls [2, 15, 18].

Balance and muscle strength tests can be used to assess and monitor individual’s health over time, and predict multi-morbidity, dependence in basic activities of daily living (ADLs) and early mortality [18–22]. Such tests also are of substantial value in predicting future health status and functional performance in older adults [22].

Numerous performance-based clinical tests assessing balance and/or muscle strength exist. Tests of grip strength, walking speed, sit-to-stand, and standing balance are shown to be markers of both current and future health [1, 18–21]. As a result, there is an increased interest in these tests and their potential use as simple screening tools in the general population to identify people who may benefit from targeted interventions aimed at preventing functional decline [1, 18, 23, 24].

However, in order to test balance and muscle strength adequately, it is important that the tests are sufficiently challenging since an early detection of loss of balance and muscle strength is important to prevent age-related functional decline in young seniors [25–29]. For young seniors, generally functioning at a higher level, it is questionable whether existing balance and muscle strength tests are sensitive enough to detect early subtle balance declines [1, 23]. Balance is a complex composite of multiple body systems including the ability to align different body segments and to generate multi-joint movements to effectively control body position and movement [30]. Since balance is highly task-specific, several aspects need to be assessed which can be categorized into static steady-state balance (i.e., maintaining a steady position in sitting or standing), dynamic steady-state balance (i.e., walking), proactive balance (i.e., anticipating a predicted disturbance such as crossing or walking around an obstacle), and reactive balance (i.e., compensating for a disturbance) [30]. Recent systematic reviews of the literature on balance tests have shown that widely used assessment tools such as the Berg Balance Scale (BBS) or Short Physical Performance Battery (SPPB) show ceiling effects in community-dwelling, healthy older adults aged 60 years and over [23, 31]. Ceiling effects of these instruments in higher functioning older adults will hamper the detection of early balance deficits, and thus intervention-related changes over time may not be detected [32, 33]. Although some balance tests such as the Fullerton Advanced Balance (FAB) scale [34], are developed for use in higher functioning older adults, these tests typically do not include tasks that challenge balance for the specific population of healthy, higher functioning older adults [35, 36].

For muscle strength, commonly used tests such as the Five times sit-to-stand (5STS) are not challenging enough in order to detect risk factors in higher functioning older adults [37]. Especially with regard to confirming the effects of an intervention, such tests have ceiling effects as most older adults can perform the test effortlessly and therefore do not show changes in performance level [37].

At present, no systematic literature review has examined which balance and muscle strength tests are used for the population of young seniors. The aim of this systematic review was to 1) identify any performance-based clinical tests used to measure balance and/or muscle strength in young seniors aged 60–70 years, and 2) evaluate the measurement properties of the most commonly used performance-based clinical balance and muscle strength tests.

## Methods

### Study design

The study is a two-step systematic literature review with two separate literature searches. The first step included the search and systematic review of performance-based clinical tests used for measuring balance or muscle strength in young seniors.

The second step included a search and a systematic review of methodological studies evaluating the measurement properties of performance-based clinical tests that have been used in ≥3 studies identified in step one.

### Search strategy

**Fig. 1:**
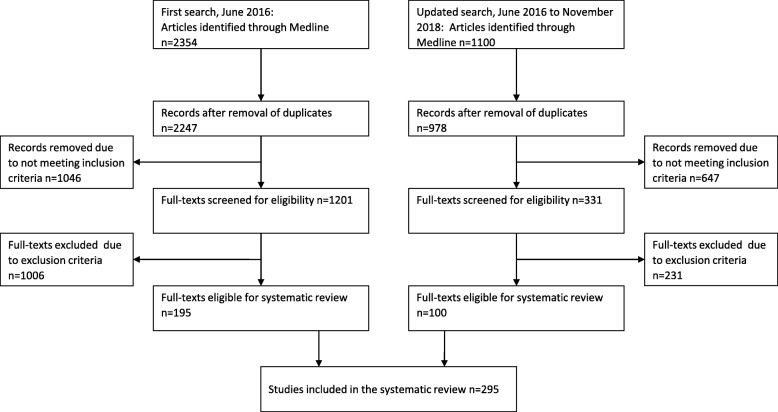
Study selection of performance based tests through the different phases (first search)

**Fig. 2:**
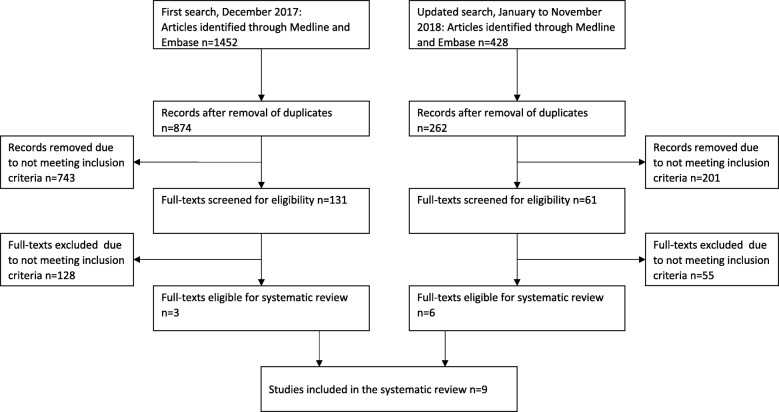
Study selection of method studies through the different phases (second search)

The search in step one was performed in MEDLINE to identify relevant studies published until June 1st 2016, with an update made to identify also newer studies published until November 5th 2018 (Fig. 1). A combination of free-text and MeSH-terms was used that represents the following concepts: ‘postural balance’, ‘muscle strength’, ‘movement’, motor activity’, ‘physical exertion’, ‘physical endurance’, ‘exercise tolerance’, and ‘physical fitness’. Additional search terms aimed to exclude animal studies, participants outside our target age group, and non-English studies (see Additional file 1). The search in step two was performed in MEDLINE and EMBASE to identify relevant method studies published until December 19th 2017, and also updated to include newer studies published until November 23rd 2018 (Fig. 2). We combined a search on the most commonly identified tests (≥3 articles) with a search on measurement properties, including validity, reliability, sensitivity, accuracy, responsiveness, and specificity (see Additional file 1).

### Inclusion/exclusion criteria

In the first step, articles were included if they (1) described a performance-based clinical test that measured aspects of balance and/or muscle strength, (2) included participants with an age or mean age between 60 and 70 years, and (3) were written in English. Articles were excluded if (1) in principal the test could not be completed without fixed laboratory equipment, (2) all groups were included on the basis of having a clinical condition (i.e., no healthy and/or control groups), and (3) manuscripts were reviews, books, posters, or conference proceedings. In the second step, articles were included if they (1) described a performance-based clinical test that was used in at least 3 studies identified in the first search, (2) evaluated one or more measurement properties in one or more of the tests described, and (3) included participants with an age or mean age between 60 and 70 years.

For the selection of articles in the first part of the study, two authors performed independent reviews of article abstracts. Discrepancies were discussed until agreement was achieved; if not, a third reviewer made the final decision. The tests detected were labelled “in-lab” when they required advanced, fixed lab equipment, or “out-of-lab”, if in principal they could be performed in a home setting. Despite gait speed being a very common measure of physical performance in older adults, it is not a specific measure of balance or muscle strength, but rather considered to be a general measure of health and function [38, 39]. Therefore we included only articles with tests of gait speed if the test included one or more additional test elements that challenge the sensory system beyond that of normal or fast walking and thus require a balance reaction (i.e. dynamic, proactive or reactive). Test batteries were included if one or more of the tests in the battery was in accordance with our definition of a performance-based test of balance and/or strength.

The review of full-texts was completed by three of the authors where one reviewed all articles and two reviewed one-half each. Discrepancies were discussed with one of the other reviewers and a decision was made based on consensus. For the second part of the study, two authors each screened one-half of the abstracts and full-texts of the methodological studies.

### Data extraction

Information from each full-text article was extracted into an excel sheet, containing information about the performance-based clinical tests (name of the test, measurement unit, scoring, and sample characteristics).

Results were categorized into sections representing balance or muscle strength measures. Since balance tests are task-specific, balance tests were categorized according to the framework of Shumway-Cook and Woollacoot [30, 1) static steady-state balance (i.e., maintaining a steady position in sitting or standing), including measures of postural sway obtained during quite standing (e.g. CoM sway); (2) dynamic steady-state balance (i.e., walking); (3) proactive balance (i.e., anticipating predicted disturbances such as crossing or walking around an obstacle); (4) reactive balance (i.e., compensating disturbances); and (5) results of balance test batteries. Muscle strength tests were categorized according to a previous published qualitative review [10], resulting in the following categories: (1) 1 Repetition Maximum (1RM); (2) Maximum Isometric Strength (MIS); and (3) Muscle Power.

### Assessment of measurement properties

The quality of the method studies included in the second step was evaluated by three independent reviewers using the COSMIN checklist [40]. COSMIN describes how to rate the quality of the following nine categories of measurement properties: internal consistency, reliability, measurement error, content validity, structural validity, hypotheses testing, cross-cultural validity, criterion validity, and responsiveness, with several items within each category [40]. Each category is rated as “poor”, “fair”, “good” or “excellent”, with a “worse-score-count”-approach, meaning that each category will get the lowest rating achieved for any of the items within that category [40]. As the criteria of each rating score can be different between categories, the method studies receive a rating for each measurement property assessed. Thus the quality of a study evaluating validity and reliability of a test can be rated “poor” for its assessment of validity, and “fair” for its assessment of reliability. Two amendments were made to the COSMIN guidelines. The first refers to the handling of missing cases. Because missing cases largely is an issue with questionnaires and not tests of physical performance, it was not considered relevant for the quality assessment, and thus articles were not given negative ratings for not addressing it. The second refers to sample sizes. Articles with sample sizes between 21 and 30 were rated as “fair” instead of “poor”, as the sample size affects the precision of estimates rather than the quality of the methodological study itself [41].

## Results

### Study selection

Out of 3454 articles identified, 295 articles were included in the full-text review (Fig. 1). In total, 69 balance tests and 51 muscle strength tests were identified (Table 1; Additional file 2). Out of these tests, 26 balance tests and 15 muscle strength tests were used in ≥3 articles. These tests were included in the second search on measurement properties, and revealed only three method studies from reviewing 874 abstracts and 131 full-text articles (Fig. 2).

**Table 1:** Summarized description of balance and strength tests

Balance test	N^a^	Age
Static steady-state balance		
Side-by-side, eyes open, 10 s (8 studies)	21,419	40–87 (62.6–70.4)
Side-by-side, eyes closed, 10 s (1 study)	37	60–81 (67.7 ± 5.3)
Side-by-side, eyes open, 30 s (10 studies)	14,003	52–90 (62.7–71.6)
Side-by-Side, on foam, eyes open, 30 s (1 study)	122	69.7–71.6
Side-by-side, eyes closed, 30 s (7 studies)	364	57–75 (64.7–71.6)
Side-by-side, 60 s (1 study)	54	60+ (66.0 ± 5.0)
Semi-tandem, 10 s (6 studies)	16,926	40–87 (62.6–70.0)
Semi-tandem, 30 s (4 studies)	13,416	52–90 (62.7–65.0)
Tandem, 10 s (8 studies)	17,100	40–87 (62.6–71.6)
Tandem, 30 s (3 studies)	13,410	52–90 (64.8–65.0)
Tandem, 60 s (1 study)	12	69.0 ± 3.0
OLS (5 studies)	2266	52–84 (64.0–69.1)
OLS, no time limit (3 studies)	718	50–79 (53.9–73.1)
OLS, eyes closed, no time limit (4 studies)	391	50–79 (60.0–67.1)
OLS, 15 s (1 study)	19	60–68
OLS, 25 s (1 study)	26	59.7–60.5
OLS, 30 s (10 studies)	4773	55–84 (62.0–69.0)
OLS, eyes closed, 30 s (2 studies)	1812	60–84 (63.2–69.0)
OLS, eyes open, 45 s (1 study)	60	62.9–64.4
OLS, eyes closed, 45 s (1 study)	60	62.9–64.4
OLS, alternating eyes open and eyes closed (1 study)	557,648	66.0
OLS, 60 s (19 studies)	39,736	34–90+ (61.8–77.0)
OLS, 60 s, eyes closed (6 studies)	536	60–84 (66.3–69.4)
OLS, 120 s (1 study)	501	65–74 (69.3–69.7)
Romberg Test (5 studies)	1262	50–80 (50.8–69.0)
Sharpened Romberg (2 studies)	76	62.5–72.8
Romberg with Jendrassik maneuver (1 study)	266	65–74 (69.5 ± 3.0)
Equi Test (1 study)	55	61–83 (69.3 ± 5.5)
SOT (1 study)	23	60–78 (66.2–71.3)
CTSIB (2 studies)	61	64.0–69.0
Dynamic steady-state balance		
Tandem walk (8 studies)	260	55–85 (65.5–77.0)
Step test (2 studies)	67	53–83 (65.7–66.9)
Four Square step test (6 studies)	470	55–81 (62.0–71.5)
Step width & length, eyes open and eyes closed (1 study)	56	66.7–72.8
MSL test (2 studies)	59	60–81 (67.7–77.0)
360° turn (1 study)	282	60–74
180° turn (2 studies)	99	55+ (61.8–68.5)
6 m backwards walk (3 studies)	77	65–84 (68.9–69.7)
10-m walk under single- and dual-task condition (1 study)	54	65–80
Floor Transfer Task (1 study)	102	61.2–67.0
SEBT (2 studies)	212	65.4–68.9
Dynamic balance/agility (2 studies)	120	60–84 (66.1–69.8)
Narrow corridor walk (1 study)	40	69.8 ± 7.5 (60+)
Sideway walk test (1 study)	32	61.8 ± 4.6
Proactive balance		
TUG (92 studies)	61,826	46–99 (61.4–77.0)
Chair rise and walk (1 study)	39	65–85
8-ft Up and Go (27 studies)	4724	51–89 (62.1–70.1)
FRT (30 studies)	13,679	50–99 (61.5–71.3)
LRT (1 study)	28	57–73 (65.9–66.0)
7 m obstacle walk (1 study)	134	69.6–70.3
Curved walking (1 study)	1054	65.0 ± 7.0
Zigzag walking (1 study)	81	50–74 (59.0–61.0)
Reactive balance		
Reactive balance test (1 study)	102	65–80 (69.8–70.0)
Push and release test (2 studies)	54	65–80
Adaptive gait test (1 study)	20	61–81
Step Execution Test (2 studies)	72	60–88 (67.7–69.6)
Backwards stepping test (1 study)	36	65–75 (66.2–68.3)
Crossover stepping test (1 study)	36	65–75 (66.2–68.3)
Limits of Stability test (1 study)	30	64.2 ± 7.3
Performance batteries		
BBS (35 studies)	2324	56–88 (61.4–74.0)
SPPB (34 studies)	17,687	60–89 (65.4–72.3)
Tinetti Test / Performance Oriented Mobility Assessment (7 studies)	8166	55.0–97.6 (62.5–66.8)
PPT (2 studies)	91	60–83 (67.4–68.8)
FAB scale (7 studies)	308	52–89 (61.8–69.5)
CS-PFP-10 (1 study)	26	60+ (68.6–72.3)
PPB (4 studies)	2149	64.0–69.9
CBM (3 studies)	132	55–70 (66.4–69.9)
8-level balance scale (2 studies)	102	55–70 (66.4–69.9)
FMM (1 study)	90	65.3 ± 4.6
Strength test	N^a^	Age
One repetition maximum		
Handgrip strength (81 studies)	130,821	34–89 (60.4–70.5)
Shoulder flexor strength (1 study)	85	65–84 (69.0 ± 0.4)
Hip muscle strength (2 studies)	45	55–75 (63.7–68.4)
Knee extensor strength (1 study)	85	65–84 (69.0 ± 0.4)
Leg strength (6 studies)	272	55–75 (61.1–69.3)
Toe grasping strength (2 studies)	722	52–78 (66.3–67.6)
Maximal Isometric Strength (MIS)		
Elbow extensor strength (1 study)	26	69.2–70.0
Hip extensor strength (1 study)	39	60–78 (68.5–69.7)
Hip flexor strength (2 studies)	818	60–78 (68.5–69.7)
Hip abductor strength (2 studies)	744	61.8–68.7
Knee extensor strength (11 studies)	1595	60.78 (61.1–71.6)
Knee flexor strength (1 study)	39	60–78 (68.5–69.7)
Leg strength (6 studies)	2544	50–79 (61.4–69.0)
Ankle dorsiflexor strength (7 studies)	357	60–78 (61.8–69.7)
Ankle plantar flexor strength (5 studies)	832	50–80 (61.8–68.5)
Functional muscle power		
Upper body functional muscle power		
30 s arm curl (20 studies)	5768	51–89 (61.9–69.9)
Abdominal Strength (2 studies)	252	59–60+ (63.0–66.9)
Single forearm contractions (1 study)	32	59–85 (66.0 ± 2)
Seated medicine ball throw (1 study)	36	68.8–68.9
Lower body functional muscle power		
Five times Sit-to-Stand (61 studies)	81,289	40–90+ (58.7–71.0)
One time sit-to-stand (7 studies)	414	60–74 (61.6–69.9)
Ten times sit-to-stand (6 studies)	73,283	50–81 (62.6–69.0)
15 s Sit-to-stand (1 study)	5777	65–79 (69.8–70.1)
30 s sit-to-stand (51 studies)	7493	51–91 (61.2–71.6)
1 min sit-to-stand (2 studies)	123	55–70 (62.2–70.7)
One time kneel-to-stand (1 study)	259	60+ (67.6 ± 7.0)
Floor rise to standing (7 studies)	172	65–84 (67.0–69.3)
Five Step Test (1 study)	621	50+ (66.8–69.4)
Stair climbing (2 studies)	1143	55–79 (63.8–67.5)
Stair climbing (8 steps) (2 studies)	111	65.6–67.8
Stair climbing (10 steps) (3 studies)	212	50–75 (62.7–71.5)
Stair climbing (11 steps) (3 studies)	77	65–84 (68.9–69.3)
Stair climbing (12 steps) (2 studies)	337	45–80 (58.7–64.8)
Stair climbing (14 steps) (1 study)	30	68.5 ± 5.1
Stair climbing (15 steps) (1 study)	134	69.6–70.3
Stair ascent (23 steps) (1 study)	62	60–83 (66.6–71.0)
Stair ascent (16 steps) (1 study)	48	60–80 (68.6 ± 6.1)
Stair ascent (10 steps) (4 studies)	158	62–80 (66.0–70.0)
Stair ascent (9 steps) (2 studies)	71	62.7–70.0
Stair ascent (4 steps) (1 study)	33	60–74 (64.4–65.7)
Stair ascent (one time) (1 study)	259	60+ (67.6 ± 7.0)
Stair descent (16 steps) (1 study)	48	60–80 (68.6 ± 6.1)
Stair descent (14 steps) (1 study)	33	67.0 ± 4.5
Stair descent (10 steps) (1 study)	19	66.0 ± 1.0
Stair descent (9 steps) (1 study)	48	69.8–70.0
Stair descent (one time) (1 study)	259	60+ (67.6 ± 7.0)
Functional leg extensor strength (1 study)	1133	55–79 (63.8–64.1)
Lift and reach (1 min) (2 studies)	123	55–70 (62.6–70.7)
Standing long jump (2 studies)	98	50–79 (63.7 ± 1.1)
Squat jump (1 study)	63	65–70 (67.5 ± 0.4)
Single knee extension contractions (1 study)	32	59–85 (66.0 ± 2.0)

^a^The total number included was the total number of participants in all studies per balance/strength test; *OLS* One-leg standing balance, *SEBT* Star Excursion Balance Test, *TUG* Timed Up and Go, *FRT* Functional Reach Test, *LRT* Lateral Reach Test, *SOT* Sensory Organization Test, *BBS* Berg Balance Scale, *SPPB* Short Physical Performance Battery, *PPT* Physical Performance Test, *FAB* Fullerton Advanced Balance, *CS-PFP-10* Continuous Scale-Physical Functional Performance-10 item test, *PPB* Physical Performance Battery, *CBM* Community Balance & Mobility scale, *FFM* Functional Movement Measurement

All studies included young seniors, where 282 studies had a sample with a mean age between 60 and 70 years and 13 studies [42–55] included participants with an age between 60 and 70 years exclusively.

### Balance performance tests

#### Static steady-state balance tests

A total of 28 tests assessing static steady-state balance were identified. Single-activity measures (24 tests) were grouped into four main activity domains: (1) Side-by-side, (2) Semi tandem, (3) Tandem, and (4) One-leg-stand. Variations were found in performance within each category regarding (1) time (range 10–120 s), (2) vision (eyes open; eyes closed), (3) surface (firm; foam), and (4) number of trials (range 1–6 trials). The method of scoring included (1) total time (s), (2) category of time intervals (categorized according to the total time), (3) percentage of participants able to hold the position, and (4) body sway measures (e.g., displacement of the Center of Pressure, CoP; sway velocity).

Three Romberg tests were identified, with variations in (1) time (range 10–60s), (2) standing positions (Side-by-Side; Side-by-Side and Tandem; Side-by-Side, Semi-tandem, and Tandem), (3) vision (eyes open; eyes closed), and (4) incorporated muscle strength element (i.e., abduction of the upper limbs). The method of scoring included (1) total time (s), (2), scoring (categorized according to the total time), and (3) percentage (ability to hold the position for a pre-determined time). Four other tests identified were the Equi test, the Sensory Organization Test (SOT), the modified Clinical Test of Sensory Interaction in Balance (mCTSIB), assessing measures of body sway (e.g., CoP displacement), and the 8-level balance scale, scoring balance performance according to the ability to perform progressively challenging standing positions.

#### Dynamic steady-state balance tests

A total of 14 tests assessing dynamic steady-state balance were identified: (1) the tandem walk, with variations in the distance walked (9.14 m; 10 m), (2) the Step test, with variations in the demand of the activity (using the worse leg), (3) The Four Square Step Test (FSST), (4) a step width and length measuring walking test, (5) the Maximum Step Length (MSL) test, (6) the 360° turn, (7) the 180° turn, (8) the 6 m backwards walk test, (9) the 10 m walk under single- and dual-task conditions, (10) the floor transfer task, (11) the Star Excursion Balance Test (SEBT), (12) a walking test measuring dynamic balance and agility, (13) the narrow corridor walk, and (14) the sideways walk test. The method of scoring included (1) total time (s), (2) distance (step width and length), (3) number of steps, (4) number of missteps, (5) percentage (inability to complete the test), and (6) scoring (categorized according to the total time for completion of test).

#### Proactive balance tests

Eight tests for assessing proactive balance control were identified. The Timed Up and Go (TUG) test was used in 92 studies, with variations in (1) set pace (self-paced; fast paced), (2) distance walked (range 2.44–3.05 m), (3) turn (walk to a line on the floor and return; walk to a cone, turn around the cone and return), (4) chair (with/without armrests; with/without backrest; height range 40–46 cm), (5) number of trials (range 1–4), (6) incorporated cognitive (counting backwards; saying animal names) and motor (carrying a cup of water) tasks, and (7) outcome measure (s; m/s; step-related variables; phase-related movement analyses; accelerations). One study investigated the chair rise and walk test, and 27 studies the 8-ft Up-and-Go test, both tests evaluated by time (s). Another 30 studies investigated the Functional Reach Test (FRT), with variations in (1) number of trials (range 1–5), (2) arms (extending the right or left arm forward; raising both arms in front), (3) hands (making a fist; with fingers extended), and (4) distance (tip of the middle finger; position of the third metacarpal). The method of scoring included (1) maximum distance reached (cm; inches), and (2) percentage (maximum distance reached normalized to height). Four other tests were the Lateral Reach Test (LAT), evaluated by the maximum distance reached (cm), and the 7 m obstacle walk, the Zigzag walking test, and the Curved walking test, all three evaluated by the total time (s) [109].

#### Reactive balance tests

Seven tests for assessing reactive balance control were identified: (1) the Reactive Balance Test, measuring oscillations in medio-lateral and anterior-posterior directions, (2) the Push and Release Test, measuring the amount of steps needed to regain balance, (3) the adaptive gait test, measuring gait speed (m/s) and the number of step errors, (4) the Step Execution Test, measuring reaction time (ms), (5) the Backwards Stepping Test, measuring ground reaction forces (N/kg),(6) the Crossover Stepping Test, measuring ground reaction forces (N/kg), and (7) the Limits of stability test, measuring reaction time (s), movement velocity (m/s), and maximum excursion (%).

#### Performance test batteries/scales

Nine performance test batteries that included different balance tasks were identified: (1) the Berg Balance Scale (BBS) which was used in 35 studies, (2) the Short Physical Performance Battery (SPPB), which was investigated in 34 studies, (3) the Tinetti Performance Oriented Mobility Assessment (POMA), which was investigated in seven studies, (4) the Fullerton Advanced Balance (FAB) scale, which was investigated in seven studies, (5) the Physical Performance Test (PPT) with variations in the number of included items (range 7–9), (6) the Continuous Scale-Physical Functional Performance-10 item (CS-PFP-10) test, (7) the Physical Performance Battery (PPB), (8) the Community Balance & Mobility (CBM) scale, and (9) the Functional Movement Measurement (FMM). All performance test batteries used a scoring scheme (e.g., 0 ‘unable to perform’ up to 4 ‘able to perform the task safely’) for the assessment of the performance.

### Muscle strength performance

#### One repetition maximum tests

We identified six tests measuring the One Repetition Maximum (1 RM) of upper- and lower-body extremities. Eighty-one studies investigated handgrip strength, with variations in (1) the measurement instrument (electronic; hydraulic; bulb hand dynamometer), (2) testing position (sitting; standing), (3) demand (both hands; dominant hand; preferred hand; adjusted size for men and women), and (4) number of trials (1–3). The method of scoring included (1) force (kg; pounds; kg/bodyweight; pounds/square; Newton; kilopascal), (2) percentage (force scores, i.e., kg classified as weakness), and (5) outcome (mean of trials; best trial). Other studies used 1 RM of shoulder flexors, hip muscles, knee extensors, legs, or toes, either assessed by force (kg) or torques.

#### Maximum isometric strength tests

There were nine tests measuring Maximum Isometric Strength (MIS). Eleven studies used MIS tests of knee extensors, with variations in (1) outcome (mean of trials; best trial), and (2) outcome dimension (kg; N/k; percentage, i.e., muscle strength/bodyweight). Six studies evaluated leg muscle strength, assessed by force (kg). Ankle dorsiflexor MIS tests were used in seven studies, either evaluated by force (kg, N/kg) or percentage (muscle strength/bodyweight). Five studies assessed ankle plantar flexor strength by force (kg). One study included MIS tests of hip extensors, two of hip flexors and hip abductors, evaluated by force (kg) or percentage (i.e., muscle strength in relation to total bodyweight). Elbow extensor strength was measured in one study by force (kg), as well as knee flexor strength, measured by percentage (muscle strength/bodyweight).

#### Muscle power tests

We identified 36 muscle power tests. For upper-body extremities, four tests were identified. The 30 s Arm Curl Test was used in 20 studies, with variations in the weight used (2.0 kg for all participants; 2.27 kg for women and 3.63 kg for men). The test recorded the number of repetitions in 30 s. Abdominal muscle power was investigated in two studies and the number of repetitions in 30 s was recorded. Single forearm contractions, evaluated by Maximum Voluntary Contraction (MVC, in kg), and seated medicinal ball throws, measured by maximum distance reached (cm), were investigated in one study each.

For lower-body extremities, six versions of sit-to-stand (STS) were used in 128 studies, with variations in (1) method of measurement (time to perform one repetition; time to perform five repetitions (5STS); time to perform ten repetitions (10STS); number of repetitions in 15 s (15 s STS); 30 s (30s STS); 60 s (60s STS)), (2) chair (height: standard; adjusted; range 30–60 cm; with backrest; without backrest; without armrests), (3) position (back at the back of the chair; sitting in the middle of the chair; sitting in the front half of the chair; sitting on the edge of the chair), (4) time of measurement (starting/finishing in a sitting or standing position), (5) pace (self-paced; fast paced), (6) number of trials (range 1–3), and (7) outcome (mean of trials; best trial). The method of scoring included (1) total time (s), (2) repetitions, (3) scoring, (4) force (N/s in kg; W in kg), and (5) speed (stands per minute).

There were seven different types of stair climbing tests investigated in 11 studies with variations in (1) number of steps (standard flight of stairs; range 8–15 steps), and (2) method of measurement (time; stair climbing power; W).

Six studies investigated stair ascent, and 4 studies investigated stair descent. Tests varied in (1) number of stair steps (range 1–23) and (2) method of measurement (time; score).

Eight other tests for measuring muscle power of lower-body extremities were identified: (1) Lift and Reach, assessed by repetitions over 1 min, (2) Floor rise to standing, assessed by time (s), (3) Five Step Test, assessed by time (s), (4) One-Time Kneel-to-Stand, assessed by time (s), (5) Functional Leg Extensor Muscle Strength, assessed by the maximum weight in relation to bodyweight, (6) Standing Long Jump, assessed by distance (cm), (7) Squat jump, assessed by maximum ground reaction force (N*kg-1), rate of force development (N*kg-1), and force (N), and (8) Single Knee Extension Contractions, assessed by maximum work rate.

### Assessment of measurement properties

Thirty-nine tests were used in ≥3 articles that were identified through step 1. In step 2, nine studies were identified that assessed measurement properties of four balance tests/scales (10s Tandem stance, TUG, SPPB, CBM) and one strength test (5STS). The quality assessment of these nine included method studies [42, 52, 56–63] are shown in an additional file (see Additional file 3). The quality of the study that assessed validity and reliability of the 10s Tandem stance [61] was rated “poor” according to the COSMIN checklist [40]. Four studies assessed the measurement properties of the TUG, with their study quality rated “good” [42, 59] for measures of validity, and “poor” for measures of reliability [59, 60]. Three studies assessed measurement properties of the CBM, and for measures of validity, the quality of these studies were rated as “fair” [52, 58, 62], for internal consistency as “poor” [52], and for reliability as “good” [52, 62]. The quality of the study assessing the SPPB was rated “excellent” for validity and “good” for reliability [57] in younger seniors. For strength, the study assessing reliability of the 5STS was rated as “fair” [56].

## Discussion

In the first step, this systematic review identified 120 performance-based clinical tests used to measure balance and/or muscle strength in young seniors, of which 69 measured balance and 51 measured muscle strength. The TUG (92 articles), BBS (35 articles), and SPPB (34 articles) were the most used balance tests in our sample. Different variations of STS (e.g. 5STS, 30s STS) were most often used to assess muscle strength (128 articles), with the 5STS as the most commonly used test (51 articles), followed by the 30s STS (51 studies). In the second step, ten method studies were identified for the 39 performance-based clinical tests which were most commonly used. The method studies evaluated measurement properties of the 10s Tandem stance, TUG, SPPB, CBM, and 5STS n samples of young seniors.

Proactive balance was the aspect of balance that was tested most frequently, with TUG as the most frequently used test (92 articles; 61,826 participants). This finding aligns with an earlier review that found TUG to be the most used test to predict falls in healthy community-dwelling older adults aged ≥60 years [31]. TUG is fast to perform and easy to administer, and cut-offs between 12 and 13 s have shown moderate to high sensitivity and specificity in predicting falls in older adults [42, 64]. However, the TUG is a general test of mobility that provides little or no information on underlying balance deficits [30]. Performance of TUG is a relatively complex task in terms of motor performance, including a ‘sit-to-stand’-movement, walking, turning and a ‘turn-to-sit’-movement, but for young seniors, the score of total duration may not be sensitive enough to reveal early signs of functional decline [20]. The instrumented version of TUG could potentially be a more useful test of balance and mobility in higher functioning groups, as more details of the quality and quantity of the performance can be obtained objectively than merely the total duration [65].

For balance performance test batteries, BBS was the most commonly used test (35 articles; 2324 participants), closely followed by the SPPB (34 articles; 17,687 participants). BBS is widely used and has been coined the “gold standard” of balance assessment tools [66]. BBS is a significant predictor for ADL disability onset in older adults aged 80 and over [67], but in samples with a mean age in the mid-seventies it suffers from ceiling effects [68–70], even in older adults with a falls history [31]. A previous systematic review recommended the SPPB as the best performance-based tool for measuring physical function in older adults due to superior qualities related to validity, reliability, and responsiveness compared to other tests [71]. This review generally reported little ceiling effects for the SPPB in the “general (mixed) population” of community-dwelling older adults. However, when applied in higher-functioning community-dwelling older adults, the SPPB also showed ceiling effects [32, 72]. Despite being extensively used in older people in general and receiving appraisals for its measurement properties, the BBS and SPPB do not appear to be good enough for assessing physical performance in well-functioning young seniors due to ceiling effects. In this review, the method study assessing the measurement properties of the SPPB was rated “excellent” for its measure of validity and “good” for its measure of reliability [57]. However, the result of the method studies are not considered in this quality rating, but relatively high mean scores on the SPPB in this study (9.7 ± 2.0) align with the findings of other studies in healthy young seniors [32, 72].

The most frequently used muscle strength test across all categories were those including some variation of the ‘sit-to-stand’-movement (128 studies), with the 5STS (61 articles; 81,289 participants) and the 30s STS (51 articles; 7493 participants) being the most popular among them.

The 5STS is commonly used as a test of physical performance in clinical assessments [73], and is also part of the SPPB test battery. We found a large variety in how this test was administered, thus making comparisons between versions a challenge. In the original and most applied protocol, the subject is “timed from the initial sitting position to the final standing position at the end of the fifth stand” [74]. In an earlier meta-analysis, the mean score on 5STS from 4184 participants between 60 and 69 years was 11.4 s [75]. This is relatively fast compared to identified cut-offs of 13.6 s for indication of increased disability and morbidity [76], and 15 s for predicting recurrent fallers [77]. However, as also this test lacks validation in young seniors, we have no basis for recommending this performance-based clinical test as a good measure for this specific population.

The second most used tool with a STS-variation was the 30s STS, originally developed to overcome floor effects of the 5STS [78]. We did not identify any method study that assessed the measurement properties of 30s STS, but in community-dwelling adults with a mean age of 70.5 ± 5.5 years, the test-retest reliability (ICC .89) and concurrent validity was moderate, with associations with weight-adjusted 1 RM leg-press of *r* = .71 (women) and .78 (men) [78]. Therefore, the 30s STS could be suitable to measure physical performance in young seniors, but further studies are warranted to confirm this.

In the second step, nine method studies were identified, with only four out of 26 balance tests and one out of 13 strength tests having been used in ≥3 articles. It is apparent that very few of all available tests for measuring balance and/or strength have been assessed for their measurement properties in healthy young seniors. The quality of most of the method studies rated in this review ranged only from “poor” to “fair”. However, there seems to be a shift in focus towards the current target group in the literature, as indicated by the high number of new studies that was identified in the updated literature search (Figs. 1 and 2).

The CBM and the 10s Tandem Stance were two of the tests that emerged as being used in ≥3 studies in the updated search. Therefore, these tests were added to the updated search of method studies. In two of three method studies assessing the CBM [52, 58], the measures of reliability were all high (>.97) and validity good to excellent in young seniors [52, 58]. However, study quality was rated “poor” with regard to validity measures with the COSMIN checklist. The studies assessing the CBM reported no ceiling effects in young seniors due to its challenging, higher level tasks [52, 58], and the CBM could be considered a feasible tool to adequately assess balance performance in healthy, higher functioning young seniors. The study assessing the 10s Tandem Stance found that valid and reliable measures of the Centre of Pressure (COP) can be obtained from a Wii Balance Board (WBB), compared to a laboratory force plate [61]. Such a device could be a suitable tool for a home-based assessment of balance/posture measures. However, COP measures as assessed by the WBB have not been evaluated in younger seniors so far.

New method studies of tests that were already included before the updated search, such as TUG, SPPB, and 5STS, indicate that not only new tests, but also well-established tests are evaluated for their potential suitability in measuring balance and/or strength in young seniors. The TUG showed excellent reliability, but both studies were rated as “poor” regarding their overall methodological quality [59, 60]. Another study, rated “good” according to COSMIN, found cut-off scores of 12.47 s on the TUG to be an accurate measure for screening of fall risk [42], while another study reported low discriminative ability of the TUG for healthy older adults vs. older adults with a history of falls [63], which is in line with previous findings concluding that the TUG is able to discriminate between fallers and multiple fallers, but not between non-fallers and fallers [79].

Based on the findings in this review, there seems to be only one promising scale for adequately assessing balance in healthy young seniors, i.e. showing no ceiling effects and having measures of high validity and reliability, namely the CBM, However, important measures such as responsiveness to identify intervention-related changes are currently lacking for this balance scale.

A limitation of this systematic review is the restriction to English written articles which might have influenced the final number of identified tests. However, this review was based on a broadly designed literature search which aimed at getting a broad overview of existing performance-based clinical tests used for measuring balance and/or muscle strength in young seniors. Due to the large number of identified and included articles, our search is unlikely to have missed any frequently used tests.

## Conclusion

This systematic review identified a large number of performance-based clinical tests that have been used to measure balance and/or muscle strength in young seniors. The most commonly used balance tests suffer from ceiling effects in young seniors. Additionally, there is a wide variety and hence lack of consensus on how to administer balance and muscle strength tests, and how to report their outcomes. There is a need for guidance on how to administer and conduct balance and strength tests to improve their informative value and comparability of outcomes. Only nine method studies were identified that assessed the measurement properties of tests used in young seniors, indicating that more studies are required to identify suitable tests for assessing balance and strength in young seniors. Only in the last 2 years, three studies assessing the measurement properties of the CBM in healthy young seniors have been identified, indicating that it could be a promising tool to adequately measure balance. The CBM has a standardised assessment procedure and studies show that it is the only scale applied in young seniors not showing ceiling effects [52, 58], being more challenging and thus more sensitive to detect changes in balance performance in healthy younger seniors. However, more research is needed to further analyse its measurement properties, especially in terms of responsiveness and sensitivity to change [52, 58, 62].

In general, more challenging tests are needed to adequately assess young senior’s physical performance, especially when aiming to identify early declines in function so that preventive strategies can be initiated in a timely manner.

## Additional files

Additional file 1: Database search. Brief description: includes all search strings for MEDLINE and EMBASE for both, part 1, i.e., identifying existing tests and part 2, i.e., identifying methodological studies for identified tests which have been used in ≥3 studies (identified thorugh part 1). (DOCX 15 kb)

Additional file 2: Description of balance and strength tests. Brief description: Large table which contains all identified balance and strength tests with detailed description of test administration, scale design, and study population. (DOCX 1691 kb)

Additional file 3: The quality of studies assessing validity and/or reliability of included balance and strength tools and the rating of the reported results. Brief description: Overview of the identified methodological studies. (DOCX 28 kb)

## Abbreviations

ADL: Activities of Daily Living
BBS: Berg Balance Scale
CBM: Community Balance and Mobility Scale
COM: Center of Mobility
COP: Center of Pressure
COSMIN: Consensus-based Standards for selection of health Measurement Instruments
CS-PFP-10: Continous Scale-Physical-Functional-Performance-10 item test
FAB: Fullerton Advanced Balance Scale
FMM: Functional Mobility Measurement
FRT: Functional Reach Test
FSST: Four Scare Step Test
LRT: Lateral Reach Test
mCTSIB: modified Clinical Test of Sensory Interaction in Balance
MIS: Maximum Isometric Contraction
MSL: Maximal Step Length
MVC: Maximum Voluntary Contraction
POMA: Performance Oriented Mobility Assessment
PPB: Physical Performance Battery
PPT: Physical Performance Test
RM: Repetition Maximum
SEBT: Star Excursion Balance Test
SOT: Sensory Organization Test
SPPB: Short Physical Performance Battery
STS: Sit to Stand
TUG: Timed Up and Go
WBB: Wii Balance Board
