# Supplementary material for: Performance-based clinical tests of balance and muscle strength used in young seniors: a systematic literature review
Source: BMC Geriatr. 2019 Jan 9;19:9. doi: 10.1186/s12877-018-1011-0 (PMC6327480; doi:10.1186/s12877-018-1011-0)
Supplement: Supplementary file 1 — Database search. Brief description: includes all search strings for MEDLINE and EMBASE for both, part 1, i.e., identifying existing tests and part 2, i.e., identifying methodological studies for identified tests which have been used in ≥3 studies (identified thorugh part 1). (DOCX 15 kb) [file 12877_2018_1011_MOESM1_ESM.docx]

**Additional file 1
Database searches**

Search for performance-based tests in MEDLINE (from 1946) to 5 November 2018 (last update).

1. ((young or younger or early) adj2 (retired or retirement or elderly or senior*1 or elder*1)).ti,ab.
2. (older adult*1 or older healthy adult*1 or older active adult*1 or older healthy individual*1 or older active individual*1 or older active men or older active women or older healthy men or older healthy women).ti,ab.
3. ((year*1 or age or aged) and ("50-70" or "50-65" or "51-69" or "55-70" or "55-69" or "60-70" or "60-65" or "61-69")).ab.
4. 1 or 2 or 3
5. muscle strength/ or movement/ or motor activity/ or physical exertion/ or physical endurance/ or exercise tolerance/ or physical fitness/ or postural balance/
6. (measured or measurement* or measuring or assess* or test*1 or scale*1).ti,ab. or Geriatric assessment/ or Anthropometry/ or outcome*.mp.
7. (fitness or physical function or physical performance or balance or strength).ti,ab.
8. limit 7 to medline
9. 7 not 8
10. (gait or leg*1 or walking or walk or knee or knees or postural sway or stand or standing or lower extremit* or lower limb*1).mp. or (go or step or steps or stepping).ti,ab.
11. (4 and (5 or 9) and 6 and 10) not animals/

Search for methodological studies of identified performance-based tests in MEDLINE (from 1946) and EMBASE (from 1974) to 23 November 2018 (last update).

1. (tandem walk* or tandem stand* or (side-by-side and (stand or feet or standing)) or feet together or semi-tandem or one leg* stand* or step test or timed up go or "8 foot up" or eight foot or functional reach or (grip strength and (measur* or test* or assess*)) or arm curl or sit to stand or chair stand or chair rise or stair climbing or stair ascent or isometric strength or handheld dynamomet* or performance oriented mobility scale or tinetti or fullerton advanced balance scale or berg balance or short physical performance battery).m_titl.
2. Observer variation/ or "Predictive value of tests"/ or Psychometrics/ or psychometr*.ti. or Reference Values/ or exp "Reproducibility of Results"/ or "Sensitivity and Specificity"/ or Validation studies.pt. or Evaluation Studies.pt. or accura*.ti. or clinimetr*.ti. or consisten*.ti. or develop*.ti. or discrimina*.ti. or feasib*.ti. or predictiv*.ti. or propert*.ti. or psychometr*.ti. or reliab*.ti. or repeatab*.ti. or reproducib*.ti. or responsive*.ti. or sensitiv*.ti. or specificity*.ti. or subscale*.ti. or suitab*.ti. or test-retest.ti,ab. or useful*.ti. or utility.ti. or valid*.ti. or varia*.ti.
3. 1 and 2

Search for methodological studies of newly identified performance-based tests in MEDLINE (from 1946) and EMBASE (from 1974) to 23 November 2018 (last update).

1. tandem stan* or standing balance or short physical performance battery or SPPB or ankle dorsiflexor or floor transfer or sit* ris* test or "community balance and mobility scale" or "timed up and go" or "instrumented timed up and go" or itug
2. Observer  variation/  or "Predictive  value of tests"/  or Psychometrics/ or  psychometr*.ti. or Reference  Values/ or exp "Reproducibility  of Results"/ or "Sensitivity and  Specificity"/ or Validation studies.pt. or  Evaluation Studies.pt. or accura*.ti. or clinimetr*.ti.  or consisten*.ti. or develop*.ti. or discrimina*.ti. or  feasib*.ti. or predictiv*.ti. or propert*.ti. or psychometr*.ti.  or reliab*.ti. or repeatab*.ti. or reproducib*.ti. or responsive*.ti.  or sensitiv*.ti. or specificity*.ti. or subscale*.ti. or suitab*.ti. or test-retest.ti. or  useful*.ti. or utility.ti. or valid*.ti. or varia*.ti.
3. 1 and 2
