# Supplementary material for: Performance-based clinical tests of balance and muscle strength used in young seniors: a systematic literature review
Source: BMC Geriatr. 2019 Jan 9;19:9. doi: 10.1186/s12877-018-1011-0 (PMC6327480; doi:10.1186/s12877-018-1011-0)
Supplement: Supplementary file 2 — Description of balance and strength tests. Brief description: Large table which contains all identified balance and strength tests with detailed description of test administration, scale design, and study population. (DOCX 1691 kb) [file 12877_2018_1011_MOESM2_ESM.docx]

**Additional file 2** Description of included balance and strength tests

| **Test characteristics** | | | **Scale** | | **Study population** | | | |
| --- | --- | --- | --- | --- | --- | --- | --- | --- |
| **Balance test** | **Detailed Description** | **Unit** | **Level** | **Items** | **N^a^** | | **Age** | **Sex^b^** |
| **Static steady-state balance** | | | | | | | | |
| Side-by-side, eyes open, 10 s  (8 studies) | Holding the position [1-7]  Three trials [8] | Time (s) [3, 4, 6]  Score [1, 5]  Sway velocity CoG (degrees/s) [8]  % of participants able to hold the position [2, 7] | N; O; R | 1 | 21419 | | 40-87  (62.6-70.4) | 12005 F, 9294 M |
| Side-by-side, eyes closed, 10 s  (1 study) | Three trials [8] | Sway velocity CoG (degrees/s) | R | 1 | 37 | | 60-81  (67.7±5.3) | 28 F, 9 M |
| Side-by-side, eyes open, 30 s  (10 studies) | Holding the position, eyes open [9-11]; with and without cognitive task, two trials [12]  Comfortable foot position with their arms by their side [13]  Habitual standing posture with arms by their side, feet hip-width apart, in their usual toe-out angle, looking straight ahead at a dot positioned at eye level on a plain background, approximately 1 m away [14]  Two trials (with audio-biofeedback; without audio-biofeedback) [15]  Two trials (comfortable stance with eyes open; narrow stance with eyes open) [16, 17]  Three trials (eyes open; visual feedback about the performance from the computer screen; following visual cues on the computer screen to move the body to hit targets identified on the screen) [18]  Twelve 30-s trials, randomly completed: three with no postural threat, nine with a possible perturbation (a push forward or pull backward to the upper trunk by the examiner; three of nine with a perturbation after 30s, six of nine with a perturbation at 1s, 5s, 10s, 15s, 20s or 25s [19] | Time (s) [9, 11]  CoP displacement (mm [16, 17]; cm [10, 13, 14])  % of time that the trunk tilt within specified angle limits; RMS and MPF of trunk tilt [15]  Postural control, movement time, path length [18]  Trunk roll/trunk pitch angle and velocity [19]  Velocity (cm/s) [14]  ML, AP, vertical acceleration and RMS [12] | R | 1-12 | 14003 | | 52-90  (62.7-71.6) | 6793 F, 7142 M |
| Side-by-Side, on foam, eyes open, 30 s  (1 study) | Holding the position [10] | CoP displacement (cm) | R | 1 | 122 | | 69.7-71.6 | 90 F, 32 M |
| Side-by-side, eyes closed, 30 s  (7 studies) | Holding the position [10, 18]; with and without cognitive task, two trials [12]  Two trials (comfortable and narrow stance) [16, 17]  Comfortable foot position with their arms by their side [13] | CoP displacement (mm [16, 17]; cm [10, 13])  Postural control, movement time, and path length [18]  ML, AP, vertical acceleration and RMS [12] | R | 1-2 | 364 | | 57-75  (64.7-71.6) | 258 F, 138 M |
| Side-by-side, 60 s  (1 study) | Two trials [20] | CoP displacement (cm) | R | 1 | 54 | | 60+  (66.0±5.0) | 30 F, 24 M |
| Semi-tandem, 10 s  (6 studies) | Holding the position [2-7] | Time (s) [3, 4, 6]  Score [5]  % of participants able to hold the position [2, 7] | N; O; R | 1 | 16926 | | 40-87  (62.6-70.0) | 9482 F, 7378 M |
| Semi-tandem, 30 s  (4 studies) | Holding the position [9, 12, 21]  Two trials (with audio-biofeedback; without audio-feedback) [15] | Time (s) [9, 12, 21]  % of time that the trunk tilt within specified angle limits; RMS and MPF of trunk tilt [15] | R | 1-2 | 13416 | | 52-90  (62.7-65.0) | 6444 F, 6964 M |
| Tandem, 10 s  (8 studies) | Holding the position [2-4, 6, 7, 10, 22, 23] | Time (s) [3, 6]  CoP displacement (mm [4]; cm [10, 22])  % of participants able to hold the position [2, 7, 23] | N; R | 1 | 17100 | | 40-87  (62.6-71.6) | 9494 F, 7503 M |
| Tandem, 30 s  (3 studies) | Holding the position [9, 12, 21] | Time (s) | R | 1 | 13410 | | 52-90  (64.8-65.0) | 6444 F, 6964 M |
| Tandem, 60 s  (1 study) | Three trials [24] | Time (s) | R | 1 | 12 | | 69.0±3.0 | 12 F, 0 M |
| OLS  (5 studies) | N/A [25-29] | Time (s) [25-29]  Score [28]  n (%) balance lost <5s [27] | N; O; R | 1 | 2266 | | 52-84  (64.0-69.1) | 1197 F, 1069 M |
| OLS, no time limit  (3 studies) | On the dominant leg [30]  On the right leg [31]  Three trials [32] | Time (s) | R | 1 | 718 | | 50-79  (53.9-73.1) | 409 F, 309 M |
| OLS, eyes closed, no time limit  (4 studies) | Holding the position as long as possible on the dominant leg with eyes closed [33, 34]  Three trials with eyes closed [35]  Lifting one leg from the floor as long as possible [36] | Time (s) | R | 1 | 391 | | 50-79  (60.0-67.1) | 176 F, 38 M |
| OLS, 15 s  (1 study) | Three trials on each leg with eyes open and eyes closed [37] | CoP displacement (cm) | R | 2 | 19 | | 60-68 | 9 F, 10 M |
| OLS, 25 s  (1 study) | Two trials on each leg [38] | n (%) able to hold  20 s | N | 2 | 26 | | 59.7-60.5 | 18 F, 8 M |
| OLS, 30 s  (10 studies) | Holding the position [12, 39-42]; average of three trials [43]; best of two trials [44]  Holding the position on each leg [45]; two trials each leg, best of all four trials [46]  Three trials on each leg, eyes open [47]  Dominant limb, contralateral knee remaining at 90°, arms folded across the chest, head straight [48] | Time (s) | R | 1-2 | 4773 | | 55-84  (62.0-69.0) | 1677 F, 1844 M |
| OLS, eyes closed, 30 s  (2 studies) | Three trials on each leg [47, 49] | Time (s) | R | 1-2 | 1812 | | 60-84  (63.2-69.0) | 927 F, 885 M |
| OLS, eyes open, 45 s  (1 study) | Arms crossed over the chest [50] | Time (s) | R | 1 | 60 | | 62.9-64.4 | 53 F, 7 M |
| OLS, eyes closed, 45 s  (1 study) | Arms crossed over the chest [50] | Time (s) | R | 1 | 60 | | 62.9-64.4 | 53 F, 7 M |
| OLS, alternating eyes open and eyes closed, 45 s  (1 study) | Arms crossed over the chest; mean and best of three trials [51] | Time (s); categorization as normal (>9s eyes open; >5s eyes closed) | N; R | 2 | 557648 | | 66 | N/A |
| OLS, 60 s  (19 studies) | Holding the position on the dominant leg [52-55]  Holding the position, eyes open [56-58]  One trial on each leg [59, 60]  Two trials on the preferred leg [8, 61]  Two trials on each leg [16, 17, 62-64]  Three trials (two trials on the preferred leg; one trial on the opposite leg) [24]  On the preferred leg, opposite knee flexed, arms at their sides; best of two trials [52]  Standing on solid surface (floor) or compliant surface (double-folded 2 cm thick exercise mat) [65] | Time (s) | R | 1-2 | 39736 | | 34-90+  (61.8-77.0) | 21542 F, 18239 M |
| OLS, 60 s, eyes closed  (6 studies) | Holding the position [56, 57, 66, 67]  Two trials on each leg [68]  Standing on solid surface (floor) or compliant surface (double-folded 2 cm thick exercise mat) [65] | Time (s) | R | 1-2 | 536 | | 60-84  (66.3-69.4) | 211 F, 291 M |
| OLS, 120 s  (1 study) | One trial on each leg [69] | Time (s) | R | 2 | 501 | | 65-74 (69.3-69.7) | 279 F, 222 M |
| Romberg Test  (5 studies) | N/A [39]  Feet together and tandem stand, both stances with eyes open and eyes closed, 10 s [70]  Parallel, semi-tandem and tandem stand, 10 s [4]  Standing with both feet together and eyes closed, 60 s [71]  Four progressively challenging conditions; in the fourth condition, participants had to maintain balance on a foam-padded surface with their eyes closed, thereby reducing visual and proprioceptive inputs and increasing their reliance on vestibular inputs; each for 30 s [72] | Time (s) [39, 70, 71]  Score [4]  Pass/fail (%) | N; O; R | 1-4 | 1262 | | 50-80  (50.8-69.0) | 215 F, 181 M |
| Sharpened Romberg  (2 studies) | Bipedal position with eyes open and eyes closed [73, 74] | Time (s) | R | 2 | 76 | | 62.5-72.8 | 26 F, 50 M |
| Romberg with Jendrassik maneuver  (1 study) | Standing with both feet together, eyes closed and performing abduction of the upper limbs for 30 s [30] | n (%) able to hold the position >20 s | R | 1 | 266 | | 65-74  (69.5±3.0) | 142 F, 124 M |
| Equi Test  (1 study) | Twelve 20-s trials in a side-by-side position; 6 conditions (each condition twice): (1) normal vision, fixed support, (2) eyes closed, fixed support, (3) vision sway-referenced, fixed support, (4) normal vision, support sway-referenced, (5) eyes closed, support surface sway-referenced, (6) vision and support surface both sway-referenced [75] | N/A | R | 6 | 55 | | 61-83  (69.3±5.5) | 36 F, 19 M |
| SOT  (1 study) | Six 20-s trials, standing on a force platform, with the platform and/or visual surround sway referenced, according to subject’s anteroposterior sway (1-3 motionless platform, 4-6 sway-referenced platform) [76] | Body sway angles | R | 6 | 23 | | 60-78  (66.2-71.3) | 0 F, 23 M |
| CTSIB  (2 studies) | Standing with hands at the sides, feet together, completing 6 sensory scenarios with various visual and supporting conditions; Visual disturbance provided by a rotating RDT image without a central rod [77]  Modified, i.e. four 30-s trials: quite standing on a firm surface with eyes open and eyes closed, quite standing on a compliant (foam) surface with eyes open and eyes closed [22] | CoP displacement (cm) [22]  Postural sway acceleration (m/s^2^) [77] | R | 4 | 61 | | 64.0-69.0 | 19 F, 5 M |
| 8-level balance scale  (1 study) | Side-by-side standing, narrow base Romberg (eyes open; eyes closed), semi tandem (eyes open), tandem (eyes open; eyes closed), one leg stand (eyes open; eyes closed; eyes closed with cognitive distractor). Participants have to complete successfully a balance task for 30 s before progressing to the next task. The highest level performed successfully was rated [78, 79] | Score (0-8) | O | 9 | 102 | | 55-70  (66.4-69.9) | 78 F, 25 M |
| **Dynamic steady-state balance** | | | | | | | | |
| Tandem walk  (7 studies) | On a beam, two trials [80]  3 m [78, 79]  6 m [81]; placing one foot in front of the other making sure that, with each step, the heel of one foot is directly in front of the toes of the other foot; walking forwards as fast as possible without falling or making any mistakes [42]  10-foot line, as quickly as possible without making mistakes (i.e. stepping completely off the line or failing to follow a heel-to-toe pattern), three trials [24]  Pre-marked 9.14m tape line on the floor [82]  On level ground and on a slightly-raised balance beam, both with and without the use of the anchors (i.e. two flexible cables, whose ends participants hold in each hand, to which 125g weights are attached at the opposing ends, and which rest on the ground; as the participants walk, they pull on the cables, dragging the anchors; 20 randomized trials [83] | Time (s) [24, 78, 79, 81, 82]  Number of missteps [24, 42]  n (%) who failed [80]  Step speed; single-, double-support duration; trunk acceleration [83] | O; R | 1 | 260 | | 55-85  (65.9-77.0) | 169 F, 48 M |
| Step test  (2 studies) | Stepping one foot on, then off, a 7.5-cm block as quickly as possible in 15 s [84]; with the worse leg [85] | Number of steps on/off the block | R | 1 | 67 | | 53-83  (65.7-66.9) | 38 F, 29 M |
| Four Square step test  (6 studies) | Stepping as fast as possible in forward, sideways, and backward directions over 4 canes resting flat on the floor in a cross formation with the tips of the canes facing together, moving first in a clockwise direction and then counter-clockwise position, without touching the canes, both feet make contact with the floor in each square before moving to the next [8, 45, 86-89] | Time (s) | R | 1 | 470 | | 55-81  (62.0-71.5) | 363 F, 95 M |
| Step width & length, eyes open and eyes closed  (1 study) | Footprints recorded on a 0.9x6.1 m (3x20 ft) paper walkway, triangular (base=5 cm) shapes were cut from adhesive moleskin and attached to the soles of the shoes at the midline of the toes. A square moleskin shape (5x5 cm) was attached to the midline of the heel. A stamp pad inker was used to apply black ink to the triangular and squared shaped moleskin [74] | Distance (mm) | R | 1 | 56 | | 66.7-72.8 | 41 F, 15 M |
| MSL test  (2 studies) | Standing with the feet together and then stepping out as far as possible with the preferred leg adjacent to a yardstick taped on the floor, before returning to the starting position, two trials [8]  Stepping maximally with one leg while keeping the other leg planted and then return to the initial position in one step; for each leg and direction (front, side, back); five trials [24] | Distance (inches) | R | 1 | 59 | | 60-81  (67.7-77.0) | 50 F, 9 M |
| 360° turn  (1 study) | Making a 360° turn, allowed to use assistive devices [5] | Score | O | 1 | 282 | | 60-74 | 228 F, 54 M |
| 180° turn  (2 studies) | Standing with arms by side and feet comfortably apart and pointing to the tape, then turn 180° on the spot within a designated area marked on the floor; as fast as possible, usual footwear; started from the word “GO” and stopped when shoulders and feet facing in the opposite directions; three turns to each direction with 1-minute rest break between trials [90]  Hands by the side, positioned on a 40cm x 60cm square drawn on the ground; then turning 180° at self-selected speed by taking steps within the square to face the opposite direction [91] | Time (s), steps (n) | R; O | 1 | 99 | | 55+  (61.8-68.5) | 52 F, 47 M |
| 6m backwards walk  (3 studies) | 6m backwards walk, placing one foot directly behind the heel of the other with the shoes touching [92-94] | Time (s) | R | 1 | 77 | | 65-84  (68.9-69.7) | 44 F, 40 M |
| 10-m walk under single- and dual-task condition  (1 study) | 10 m instrumented walkway, single and dual task (walking while counting backwards aloud) conditions in 1) normal gait pattern, 2) narrow gait, 3) overlapping gait, and 4) tandem gait [70] | Stride time, stride length, stride width, stride velocity | R | 8 | 54 | | 65-80 | N/A |
| Floor Transfer Task  (1 study) | Standing upright on a mat, transferring to a sitting position on the floor mat, then returning to standing in any preferred way [95] | Time (s) | R | 1 | 39 | | 61.2±7.5 | 27 F, 12 M |
| SEBT  (2 studies) | Balancing on the stance leg and reaching with the opposite leg as far as possible, five reaches in the anterior, medial, and posterior directions, calculating the star composite reach distance, i.e. sum of the normalized reach distances for the right and left leg for all reach directions [96]  Standing at the center of a grid placed on the ﬂoor, with eight lines extending at 45° increments from the center of the grid, placing one leg in the center of the grid, with the opposite leg reaching as far as possible along the eight deﬁned directions in order to touch the furthest point on the ﬂoor as lightly as possible so as to avoid using the reach leg for support, and then return to the center of the grid without losing balance, the distance from the center of the grid to the reached point is measured [97] | Distance (cm) | R | 6 | 212 | | 65.4-68.9 | 107 F, 99 M |
| Dynamic balance/agility  (2 studies) | Rapidly standing from a chair, walking around cones, and returning to the chair [98, 99] | Time (s) | R | 1 | 120 | | 60-84  (66.1-69.8) | 43 F, 79 M |
| Narrow corridor walk  (1 study) | N/A [100] | time (s) | R | 1 | 40 | | 60+  (69.8±7.5) | N/A |
| Sideways walk test  (1 study) | Standing with the inner sides of the feet touching the starting line, and then walking sideways along a 5 m walkway at self-selected speed, i.e. abduction of the leading leg, followed by adduction of the trailing leg with the inner sides of feet touching each other; 3 trials toward both sides, randomized manner [101] | time (s), steps (n) | O; R | 2 | 32 | | 61.8±4.6 | 22 F, 10 M |
| **Proactive balance** | | | | | | | | |
| TUG  (91 studies) | As fast as possible [48, 50, 52, 81, 102, 103]  Best of three trials [104]  Two trials [105-107]; mean of two trials [108]; natural and fast speed [109]  With and without a cognitive task (saying animal names) [110]; counting backwards (substracting 3 from 100) [77]  3 m version; two trials [111]; as fast as possible [112]  Getting up from a chair with armrests, walk 3m, return and sit down again [97, 98]; walk 3 m to a mark placed on the floor [113]  Getting up from a chair, walk 3 m, turn, walk back, and sit down [9, 54, 114-117]; as quickly as possible, without running; two trials [118]; three trials [119]; habitual gait [34, 49, 70, 120, 121]; chair without armrests [95]  Rising from a chair (40 cm high), walking 3 m, turning around, and sitting down again as fast as possible; two trials [122, 123]  Rising from a chair, walking to and from a point located 3 m ahead at preferred speed, and then sitting down again [124]; two trials [61]; mean of three trials [90]; mean of two trials [107]  On a command, participants get up from an armless, backless chair (43 cm high), walk forward 3 m, turn around, walk back to the chair and sit down again [38]  On cue, participants rise from the chair, walk 3 meters to a line on the floor and return to their initial seated position [125, 126]; normal armchair (44 cm high [16]); marker of 20 cm diameter [127]  Sitting in a free-standing padded armchair, then stand up without use of arms, walk at a comfortable and safe pace to a line on the floor 3 m away, turn and walk back to the chair and sit down again [128]  Sitting in a normal chair (45 cm high), with the back against the chair, standing up, walking 3 m as quickly and safely as possible past a line on the floor, turn around, walk back to the chair, and sit down once again with the back against the chair; two trials [30]  Rising from a chair (45 cm high) without using the arms to assist, walk 3 m to a cone, turn around the cone and return to the seat [129]  Standing up from a standard chair of 45 cm height, walking 3 m, turning around a cone, returning to the chair, and sitting down again in the shortest time possible without running [78, 79]; two trials [52]  Standing up from a seated position, walk a distance of eight feet at usual pace, return to the chair, and sit back down [130]  Getting up from a chair, walking 10 ft, turning, walking back, and sitting down; three trials [54, 131]  Sitting with the back against the chair (approximately 46 cm high), on a command participants rise from a standard arm chair, wearing their own shoes and/or using an ambulatory aid, walk a distance of 10 ft, and return to the seat with their back resting against the back of the chair [132]  Sitting with the back against the chair (46 cm high, with armrests), on a command participants rise from a standard arm chair stand from a seated position, walk 3 m at their usual pace, turn around, walk back to the chair and sit down; walking aids permitted [133]  Sitting in a chair, then stand up without using the hands, walk to the end of a 10-ft pathway, turn around, walk back and sit down as quickly and safely as possible [21]  Getting up from a sitting position in an armless chair, walk 2.5 meters, return and sit down again in the same chair. A flag indicated the distance of 2.5 m from the chair; mean of three trials [134]  Sitting in a chair and on a command, standing and moving as quickly as possible around a cone placed 2.5 m away from the chair and return to the chair and sitting down [96, 135]  Sitting on a chair (43 cm high), with back support, travel a distance of 2.43 m, turn around a cone positioned at the end of the route, return, and sit down again at the chair; two trials [136]  Rising from a chair on a command, walk 8 ft, and return to sit in a chair [37]  3-trials: 1) Get up from a chair, walk 3 m straight on, turn around a cone, walk back to the chair, and sit down; 2) with an additional cognitive task (counting backwards in step 3, starting with 97), and 3) with an additional motor control task (transporting a cup of water without spilling any water during the TUG) [137]  Standing up and sitting down in a chair, walking and turning while simultaneously completing a cognitive task of counting backwards from 100 in 3’s [87, 89]  Dynaport was fixed with an elastic belt at the level of lumbar segment L3 over the participant’s clothes; Standing up from a chair without the use of the arms, walking 7 m, turning around a pion, walking 7 m back to the chair, and sitting down without the use of the arms; as fast as possible without running; two trials [138]  N/A [10, 28, 33, 35, 43, 51, 65, 86, 100, 101, 139-153] | Time (s) [9, 10, 16, 21, 28, 30, 33-35, 37, 38, 43, 51, 52, 54, 61, 65, 70, 77-79, 81, 86, 87, 89, 90, 95-98, 100-129, 131-137, 139, 141-148, 150-153] Time (m/s) [49, 140]  time (s; each phase); amplitude; range of the movement; variability; smoothness of the movement for sit-to-stand, stand-to-sit, and for the two turns; step-related variables (e.g., stride time, number of steps); ML and AP acceleration signals (measures of stability and smoothness of gait) [138] | R | 1-3 | 61826 | 46-99  (61.4-77.0) | | 9229 F, 12033 M |
| Chair rise and walk  (1 study) | Starting from a seated position, then stand up and walk as quickly as possible in a predetermined straight line to a pylon 9.14 m, go around the pylon, and return to the original seated position [82] | Time (s) | R | 1 | 39 | 65-85 | | 20 F, 19 M |
| 8-ft Up and Go  (27 studies) | Part of the SFT [29, 42, 47, 57, 154-166]  Getting out of a chair, walk 8 ft, turn around a cone, return to the chair and sit down as quickly as possible [167-171]; two trials [172]  Sitting in a chair, hands on thighs and feet flat on the floor, on a command, stand up, walk as quickly as possible around a cone placed 8 ft ahead of the chair, and return to a fully seated position on the chair [173]; two trials [174]  N/A [175, 176] | Time (s) | R | 1 | 4724 | 51-89  (62.1-70.1) | | 2581 F, 992 M |
| FRT  (30 studies) | Two trials [111], mean [107]  Three trials [177]  Part of the SFT [42, 105, 160, 162-166]  Reaching forward as far as possible without moving the feet [11, 178]  Maximum distance a person can reach forward beyond arm’s length while standing in a fixed position, three trials [76]  Measuring participant’s balance with a tape measure horizontally on the wall and the participant reaching forward as far as possible from the waist without losing balance [97]  Standing with the feet shoulder-with apart, making a fist, and raising the arm to be parallel with the floor. The assessor took an initial reading on the yardstick, using the knuckle of the third metacarpal as the landmark, then reaching forward along the yardstick without moving the feet [88]  A yard stick was positioned horizontally next to the right side of the participant at the height of the acromion; Standing naturally and raising the right arm forward to 90° (parallel to the yard stick), and at the ‘go’ signal, reach forward as much as possible at their own pace [91]  Standing and then raising both arms in front to shoulder level while the heels touch the ground [66, 67, 179]; two trials [122]  Reaching forward beyond arm’s length while maintaining a fixed base of support in the standing position; right and left arm recorded [70]; five trials [18]  Participants place their feet behind a marked line and whilst maintaining a ﬁxed base of support reach forward along a preplaced measure tape [93]  Extending the right or left arm forward, while standing with legs apart, two trials [61]  Raising the arm closest to the wall to shoulder height; the position of the third metacarpal is recorded. Subjects are instructed to keep the feet flat on the floor and lean forward as far as possible without losing balance, touching the wall, or taking a step; two trials [16]  Raising one arm at 90 degree with fingers extended. A yardstick was mounted on the wall at shoulder height. The distance that a participant could reach while extending forward from the initial upright posture to the maximal anterior leaning posture without moving or lifting the feet is visually measured in cm, according to where the middle finger tip is positioned on the mounted yardstick; two trials [123]  Standing close against a wall with a measurement tape fixed on the wall and keep the shoulder in 90° flexion parallel to the tape; reach forward maximally with arm outstretched equal to shoulder’s height without moving the feet or touching the wall; mean of three trials [180]  Standing with the feet a comfortable distance apart and behind a line perpendicular and adjacent to a wall, the arm closest to the wall is then raised to shoulder height, and the position of the tip of the middle ﬁnger is measured; feet ﬂat on the ﬂoor and leaning forward as far as possible without losing balance, touching the wall, or taking a step. The position of the tip of the middle ﬁnger is then recorded at the point of furthest reach, and the difference between the two points is recorded as the maximal distance; three trials [30]  N/A [94, 107, 151] | Distance (cm) [11, 16, 18, 30, 42, 61, 66, 67, 70, 91, 93, 94, 97, 105, 107, 111, 122, 123, 151, 160, 162-166, 179-181]  Distance (inches) [88]  % (normalized using height) [76, 178] | R | 1 | 13679 | 50-99  (61.5-71.3) | | 8577 F, 4072 M |
| LRT  (1 study) | Standing with the back to (but not in contact with) a wall, feet placed in a standardized position with 0.1 m between the most medial aspects of the heels, with each foot angle at 30°, then reaching directly sideward as far as possible without overbalancing, taking a step or touching a wall; two trials [16] | Distance (cm) | R | 1 | 28 | 57-73  (65.9-66.0) | | 3 F, 25 M |
| 7m obstacle walk  (1 study) | 7 m walk with stepping over a 30 cm obstacle at the 4 m point, normal pace; two trials [135] | Time (s) | R | 1 | 134 | 69.6-70.3 | | 85 F, 49 M |
| Zigzag walking  (1 study) | Walk along a 10-m walkway with four cones placed 2 m apart on the floor between the start and finish points as quickly as possible. The cones were set to alternate from side to side with a distance of 0.5 m from a line drawn through the start and finish points. Participants walk around the outside of each cone and walk through the finish point; two trials [182] | Time (s) | R | 1 | 81 | 50-74  (59.0-61.0) | | 40 F, 41 M |
| Curved walking  (1 study) | Walking three times (i.e., 1,080°) around a marked circle on the floor with a diameter of 1.2 m. Walks in the clockwise and counterclockwise directions were alternated to avoid the effects of direction. Participants started with the single walking trial (walking three times counterclockwise, followed by three times clockwise); followed by adding a dual task; and another dual task (subtracting serial 7 s) [183] | Time (s), n (checked boxes/subtractions) | R | 1 | 1054 | 65.0±7.0 | | 526 F, 528 M |
| **Reactive balance** | | | | | | | | |
| Reactive balance test  (1 study) | Stand erect in bipedal step stance with hands placed on hips and gaze fixated on a cross on the nearby wall on a two-dimensional balance platform. Medio-lateral perturbation impulses are unexpectedly be applied in order to investigate reactive postural control (10 s intervals); three trials [70] | Summed oscillations of the platform in medio-lateral and anterior-posterior directions | R | 2 | 54 | 65-80 | | N/A |
| Push and release test  (2 studies) | Standing in a comfortable stance with eyes open and pushing backward against a palm of the examiners' hand. After the examiner suddenly releases his or her hands, participants are required to regain balance [70, 147] | Amount of steps to regain balance | O | 1 | 102 | 65-80  (69.8-70.0) | | 26 F, 22 M |
| Adaptive gait test  (1 study) | Walking barefoot at self-selected comfortable pace within a narrow, 6.1-m-long path with a cognitive task (reciting the days of the week in reverse order); four trials [184] | gait speed (m/s), step errors (n) | R | 1 | 20 | 61-81 | | 69.1±8.6 |
| Step Execution Test  (2 studies) | Standing barefoot and upright on a force platform viewing an 'X' displayed on a screen, 3 m in front, step as quick as possible (step length 50-60 cm), following a tap cue on their heel, nine trials (forward, backward, sideward) [185]  Stand with the foot of the preferred leg on a foot-pad, and react to an auditory stimulus by stepping rapidly onto a second foot-pad 18 inches away; two trials [8] | Reaction time (ms) | R | 1 | 72 | 60-88  (67.7-69.6) | | 9 F, 28 M |
| Backwards stepping test  (1 study) | When signaled, lean as far backwards as possible, and then take a backward step with the unloaded leg; three trials (eyes open, eyes closed) [186] | Ground reaction force (N/kg) | R | 1 | 36 | 65-75  (66.2-68.3) | | 31 F, 5 M |
| Crossover stepping test  (1 study) | When signaled, lean as far laterally as possible, and then take a crossover step with the unloaded leg; three trials (eyes open, eyes closed) [186] | Ground reaction force (N/kg) | R | 1 | 36 | 65-75  (66.2-68.3) | | 31 F, 5 M |
| Limits of stability test  (1 study) | Maximum distance that each subject could shift their center of gravity (COG) without losing balance (forward, backwards, and both sides) [112] | Reaction time (RT), movement velocity (MVL), maximum Excursion (MXE) | R | 4 | 30 | 64.2±7.3 | | 19 F, 11 M |
| **Performance batteries** | | | | | | | | |
| BBS  (35 studies) | 14 balance tasks (5 static, 9 dynamic) with varied difficulty (e.g. sit-to-stand, standing with eyes open and eyes closed, tandem stand, one-leg stand, transfers, reaching for an object, a 360° turn; each scored from 0 to 4 [15, 21, 33, 47, 50, 52, 54, 65, 75, 90, 95, 98, 101, 109, 112, 113, 116, 117, 126, 132, 140, 142, 148, 149, 151, 153, 185, 187-193] | Score (0-56) | O | 14 | 2324 | | 56-88  (61.4-74.0) | 1255 F, 8728 M |
| SPPB  (34 studies) | Three hierarchical standing balance tests (side-by-side, semi-tandem, tandem position for 10 s each), 4-m walk at usual speed (m/s), and five repeated chair stands as quickly as possible (s), each scored from 0 to 4 [11, 52, 96, 100, 121, 129, 165, 176, 180, 194-217] | Score (0-12) [11, 52, 96, 100, 121, 129, 165, 176, 180, 194-204, 207, 208, 210-213, 215-217]  Score summarized in quartiles (lower body function: poor, fair, good excellent) [206]  % score (7-9; 10-12 points) [205] | O | 3 | 17687 | | 60-89  (63.0-72.3) | 10992 F, 4187 M |
| Tinetti Test / Performance Oriented Mobility Assessment  (7 studies) | Tinetti’s balance and gait evaluation [73, 103, 218]  13-item balance and 9-item gait assessment, each scored from 0 (unable) to 1 (able to perform) [219]  Static sitting balance (rising from the sitting position without using), standing balance (the first five seconds after the subject’s sternum was gently pushed by the examiner, and when stance was stabilized, staggering or excessive sway of the subject was examined with the subject standing and his eyes closed); 360° turn, observing steadiness and continuity of steps [220-222] | Score (0-28) [73, 103, 218]  Score (0-22) [219]  POMA balance score [220-222] | O | 3-28 | 8166 | | 55.0-97.6  (62.5-66.8) | 4916 F, 2524 M |
| PPT  (2 studies) | Two versions, i.e. a 9-item scale, including writing a sentence, simulated eating, 360° turn, putting on and removing a jacket, lifting a book and putting it on a shelf, picking up a penny from the floor, a 50-foot walk test, and climbing stairs (scored as two items); and a 7-item scale, not including stairs; each scored from 0-4 [85]  Nine items, including Romberg test, chair sit-to-stand, lifting a book from waist height to a shelf at shoulder level, putting on and taking off a coat, picking up a penny from the floor, 360° turn, 15 m walk, ascending one flight of stairs, climbing 4 flights of stairs; each scored from 0-4 [118] | 7-item score (0-28) [85]  9-item score (0-36) [85, 118] | O | 7-9 | 91 | | 60-83  (67.4-68.0) | 54 F, 37 M |
| FAB scale  (6 studies) | Ten static and dynamic balance tasks (stand, reach, turn in a circle, step up and over, tandem walk, one-leg stand, stand on foam with eyes closed, two-footed jump, walk with head turns, maintain a reactive posture), each scored from 0-4 [78, 79, 158, 164, 190, 223]  Task 1-4 [161] | Score (0-40)  Score (0-8) [161] | O | 10 | 308 | | 52-89  (61.8-69.5) | 187 F, 72 M |
| CS-PFP-10  (1 study) | 10 household tasks, including carrying a pot of water from one counter to another; carrying groceries onto and off a 4-step platform; transferring laundry; donning and removing a jacket; sweeping kitty litter into a dustpan; climbing stairs; sitting down and getting up from the floor; picking up 4 scarves from the floor; 6 m walk; maximal reach [202] | Score (0-100) | O | 10 | 26 | | 60+  (68.6-72.3) | 22 F, 4 M |
| PPB  (4 studies) | Modified version of the SPPB, more challenging tasks, i.e. 10 repeated chair stands, single leg stance, narrow walk (walking between 2 parallel lines separated by 20 cm) [144, 215, 224, 225] | Score [144, 215, 224]  Time (s; individual tasks) [144] | O; R | N/A | 2149 | | 64.0-69.9 | 556 F, 494 M |
| CBM  (3 studies) | 13 tasks: One-leg stance, tandem walking, 180° tandem pivot, lateral foot scooting, hopping forward, crouch and walk, lateral dodging, walking  and looking, running with controlled stop, forward to backward walking, walk, look and carry, descending stairs, and step-up x 1 step [78, 79, 226] | Score (0-96) | O | 13 | 132 | | 55-70  (66.4-69.9) | 39 F, 12 M |
| FMM  (1 study) | Seven tasks including deep squat, hurdle step, in-line lunge, shoulder mobility, active straight leg rise, trunk stability push-up, and rotary stability [227] | Score (0-21) | O | 7 | 90 | | 65.3±4.6 | N/A |
| **Test characteristics** | | | **Scale** | | **Study population** | | | |
| **Strength test** | **Detailed Description** | **Unit** | **Level** | **Items** | **Na** | | **Age** | **Sexb** |
| **One repetition maximum** | | | | | | | | |
| Handgrip strength  (81 studies) | Both hands, best trial [228]  Bi-handgrip strength, two trials [105]  Standing and then grasping a grip device; best of three trials [31]; mean of two trials [147]; best of two trials [229]  Sitting, elbow fully extended in front on shoulder height; mean of three trials [230]  Sitting position, shoulders adducted, neutrally rotated, elbow flexed at 90°, forearm neutral   - dominant hand; best of three trials [231] - both hands, three trials for each hand, best score [121]   Electronic / hydraulic dynamometer   - best trial (number not specified) [9, 40] - two trials [199] - three trials [82]; mean of three trials [196] - best of three trials [49, 57, 146, 184, 188, 232, 233] - both arms, mean of six trials [124] - standing; mean of three trials [234] - Sitting, shoulders adducted, neutrally rotated, elbow flexed at 90°, forearm neutral; mean of two trials [235]; mean of three trials [236] - dominant hand [176]; arm by side; best of three trials [168, 237] - dominant hand, sitting in an upright position, arm of the measured hand unsupported and parallel to the body; one trial [70] - dominant hand, sitting, dominant shoulder in rest position, elbow flexed 90° without support, forearm and wrist at neutral position; best of three trials [201] - dominant hand, sitting comfortably, dominant arm by side, elbow flexed 90°, hand held in mid-supination/pronation position; best of three trials [180] - both hands, one trial in each hand; best score [63] - both hands; three trials in each hand, best score for each hand [238]; best trial of the dominant hand [239] - best score of both hands [203]; best score of each hand [240]; sum of best score of each hand [111]; mean score of each hand [241] - both hands, wrist in neutral position, elbow flexed at 90°; three trials for each hand; mean of each hand [242] - both hands, two trials for each hand, mean for each hand and larger mean from one of the hands [207] - N/A [28, 91]   Bulb hand dynamometer   - both hands, holding at shoulder level, two trials in each hand; mean of both hands added [243] - dominant hand, medium (women) or large (men) dynamometer, sitting; best of three trials [71] - both hands, three trials in each hand; mean for each hand [244] - both hands, two trials in each hand; mean for each hand [245]   Calibrated dynamometer   - both hands, elbow flexed to 90° [167] - preferred hand, arm raised overhead then slowly lowered towards floor [21]; three trials [32]   Handheld dynamometer   - both hands, standing, best of two trials (each hand) [2, 59, 69, 128]; mean of three trials (each hand) [110] - both hands, two trials on the dominant and three trials on each hand [246] - two trials; mean score [197]; best score [120, 247] - dominant hand [179]; mean of two trials [248]; mean of three trials [249]; best of two trials [133] - sitting, elbow flexed two 90°, best of two trials [204]; best of three trials [250, 251] - standing with their forearms bent at 90 °, best of two trials [7] - elbow positioned at 90° of the side of the body; dominant hand; mean of three trials [252]   N/A [48, 103, 139, 150, 152, 165, 214, 216, 217, 253-261] | Force (kg) [2, 9, 31, 40, 49, 57, 69, 70, 105, 111, 128, 139, 167, 180, 184, 188, 197, 199, 201, 203, 207, 228, 231, 235, 237-243, 245-251, 253] [59, 179, 203, 204, 233, 234] [7, 32, 82, 91, 120, 121, 124, 133, 147, 150, 152, 165, 176, 214, 216, 217, 229, 230, 240, 252, 254-261]  Force (pounds) [21]  Force (kg)/bodyweight (kg) [168]  % of people with force scores (kg, classified as weakness) [196]  kg/cm^2^ [71]  Force (pound per square) [244]  Force (kPa) [28]  Force (Newton) [63] Quartiles (% lower quartile reported) [236] | R | 1-2 | 130821 | | 34-89  (60.4-70.5) | 75538 F, 49439 M |
| Shoulder flexor strength  (1 study) | Right arm, 90° shoulder flexion, elbow in full extension; mean of three trials [47] | Force (kg) | R | 1 | 85 | | 65-84  (69.0±0.4) | 37 F, 48 M |
| Hip muscle strength  (2 studies) | Supine on a plinth, both legs 10° abducted, a strap (5 cm wide) around the plinth and over the pelvis; for the examiner-resisted test, participants pushed as hard as possible against the HHD as the examiner provided resistance, stabilizing and positioning the HHD; for the belt-resisted test, HHD is placed between the side of the test leg and a second strap (5cm width), participants spread their legs apart simultaneously as hard as possible [262] Right, and left hip abduction strength on isotonic external resistance machine [263] | Torques  kg/body mass [263] | R | 2 | 45 | | 55-75  (63.7-68.4) | 31 F, 14 M |
| Knee extensor strength  (1 study) | Computer-based manual muscle testing, knee at 30° flexion; mean of three trials [47] | Force (kg) | R | 1 | 85 | | 65-84  (69.0±0.4) | 37 F, 48 M |
| Leg strength  (6 studies) | Sitting in a standard chair (45 cm high), connected to a WBB (57° angle from the ground) via custom seatbelt straps; pressing the feet on the WBB as hard and as fast as possible; three trials [264]  Dynametry [254]  Leg press machine [34, 145, 176, 263] | Force (kg)  Kg/body mass [263] | R | 1 | 272 | | 55-75  (61.1-69.3) | 140 F, 76 M |
| Toe grasping strength  (2 studies) | Barefoot, one-leg stand, both hands on the wall in front, holding the dynamometer grasping bar with the toes [265]  Sitting upright on a chair, without leaning on the backrest; both hips and knees flexed at approximately 90°; ankles placed in a neutral position and fixed with a strap [107] | Force (kg) | R | 1 | 7227 | | 52-78  (66.3-67.6) | 534 F, 188 M |
| **Maximal Isometric Strength (MIS)** | | | | | | | | |
| Elbow extensor strength  (1 study) | Instrumented wooden pole that subjects pressed against the ground; Subjects sit on a bench, with the shoulder in a neutral position, elbow angle of 90°, forearm was parallel to the ground; best of three trials [146] | MVC (kg) | R | 1 | 26 | | 69.2-70.0 | 17 F, 9 M |
| Hip extensor strength  (1 study) | HHD, mean score [178] | % (strength/body weight) | R | 1 | 39 | | 60-78  (68.5-69.7) | 15 F, 24 M |
| Hip flexor strength  (2 studies) | HHD [91], mean score [178]  HHD, sitting position, hip flexed at 90° and knee flexed at 90°; sensor of the HHD was applied to a distal site on the anterior surface of the thigh [55] | % (strength/body weight)  Force (kg) [55, 91] | R | 1 | 818 | | 60-78  (68.5-69.7) | 775 F, 313 M |
| Hip abductor strength  (2 studies) | Supine position with the hip and knee fully extended and hip positioned in neutral abduction; sensor of the HHD was applied 5 cm proximal from the lateral malleolus [55]  HHD, three trials (each hold for 3-5 s) [101] | Force (kg) | R | 1 | 744 | | 61.8-68.7 | 482 F, 262 M |
| Knee extensor strength  (11 studies) | HHD, sitting upright, raising lower legs up 90°, parallel to the ground, holding this position as strongly as possible against the maximum persistent (5 s) force applied by the examiner through the HHD placed on the front of the ankle proximal to the medial malleolus; two trials for each leg, best score [266]  HHD [91, 107]; mean score [10, 178]  Leaning back in a chair, extending both legs at the knee while pulling against a dynamometer; best of two trials [66]  Sitting on a high chair and pushing against a strap linked to a spring gauge [100]  Seated position using an adjustable chair with a 90° angle of hip and knee joints, dominant leg, as fast as forcefully, strongest of five trials [34]  Dynamometer, right side; sitting on a backwardly-inclined (5°) chair, range of motion was set from a knee joint angle of 90° to 160° (180° represents full extension); best of two trials [145]  Sitting in an upright position with back support and with both the hip and knee flexed at 70°; distal leg affixed to a strain gauge force transducer; best of three trials [146]  Dominant limb [232] | Force (kg) [34, 66, 107, 232, 266]  % (strength/body weight) [100, 178]  N/kg [10]  Peak torque (Nm) [145, 232]  MVC (kg) [146] | R | 1-2 | 1595 | | 60-78  (61.1-71.6) | 1038 F, 402 M |
| Knee flexor strength  (1 study) | HHD, mean score [178] | % (strength/body weight) | R | 1 | 39 | | 60-78  (68.5-69.7) | 15 F, 24 M |
| Leg strength  (6 studies) | Dynamometer, both legs simultaneously [267]; mean of two trials [245]; best of two trials [268]  Dynamometer, both legs simultaneously, standing with back straight against a wall and knees 115° flexed; a bar connected by a chain to the dynamometer was held in front of the thighs and has to be lifted upwards with maximum force using only the legs, and keeping the neck and back straight; mean of two trials [243, 269]  Fitted with the harness around their hips and seated in a standard chair (45 cm height) with the seatbelt straps connecting the harness to the FysioMeter-mount. The lengths of the seatbelt straps were adjusted using a tape measure and a goniometer angle between sessions to reach a knee angle of approximately 120°; foot placed in the middle of the Wii Balance Board [270] | Force (kg) | R | 1 | 2544 | | 50-79  (61.4-69.0) | 1230 F, 1277 M |
| Ankle dorsiflexor strength  (7 studies) | HHD [91, 149]; mean score [10, 178]; mean of three trials [90, 112]; three trials (each for 3-5 s) [101] | % (strength/body weight) [178]  N/kg [10]  Force (kg) [90, 91, 101, 112, 149] | R | 1 | 357 | | 60-78  (61.8-69.7) | 222 F, 135 M |
| Ankle plantar flexor strength  (5 studies) | HHD [91, 142, 149]; mean of three trials [90, 112]; three trials (each for 3-5 s) [101] | Force (kg) | R | 1 | 832 | | 50-80  (61.8-68.5) | 450 F, 392 M |
| **Functional muscle power** | | | | | | | | |
| *Upper body functional muscle power* | | | | | | | | |
| 30 second arm curl  (20 studies) | Part of the SFT [42, 45, 154, 156, 160, 162-164, 166, 170, 271]  Performing as many biceps curls as possible in 30 s, using a 2.27-kg dumbbell (full range of motion; study in women) [169, 272]  Flexing and extending the elbow of the dominant hand, lifting a weight (8 lb [3629g] dumbbell for men; 5lb dumbbell [2268g] for women) through the complete range of motion as many times as possible in 30 s [173]  Sitting on a chair, using the dominant hand to bring a weight (2.0 kg) up and down (flex and extend the biceps) as many times as possible in 30 s [179]  Hand curling a hand weight (5 pounds for women and 8 pounds for men) for 30 s [131]  Siting on the chair, holding the dumbbell (women 5 lbs, men 8 lbs) in the hand with palm facing towards the body (with the arm in a vertically down position beside the chair), bracing the upper arm against the body so that only the lower arm is moving, then curling the arm up through a full range of motion, gradually performing elbow flexion with supination; as the arm is lowered through the full range of motion, gradually return to the starting position [152]  N/A [144, 175] | Repetitions | R | 1 | 5768 | | 51-89  (61.9.0-69.9) | 2652 F, 1111 M |
| Abdominal Strength  (2 studies) | Lying down on an abdominal pad, with knees flexed at 90°, hands set on the pad frame. Rise with the chest up to approximately 30° from the floor as many times as possible in 30 s [167]  Lying on sit-up equipment and performing sit-ups with the feet attached to the equipment’s foot holders as many times as possible in 30 s [235] | Repetitions | R | 1 | 252 | | 59-60+  (63.0-66.9) | 230 F, 122 M |
| Single forearm contractions  (1 study) | Dynamic single contractions in both arms, HHD at 10%, 20%, and 40% of the subject's maximum voluntary contraction [273] | MVC (kg) | R | 1 | 32 | | 59-85  (66.0±2) | 13 F, 19 M |
| Seated medicine ball throw  (1 study) | Participants horizontally propel a 3 kg ball without trunk flexion [144] | Distance (m) | R | 1 | 36 | | 68.8-68.9 | 20 F, 16 M |
| *Lower body functional muscle power* | | | | | | | | |
| Five times Sit-to-Stand  (61 studies) | Part of the SPPB [27, 28]  Rising from a chair and sitting back down five times [9, 34, 112, 168]; without arm support [147, 240, 274, 275]; time measured at the final sitting down, best of three trials [127]; mean of three trials [90]  Five repetitive chair stands as quickly as possible with arms folded across the chest [1, 7, 41, 63, 70, 203, 246, 249]); mean of two trials [8, 276]; mean of three trials [95, 188]  Sitting in a standard chair, arms folded across the chest, standing up and sitting down five times [124]; as fast as possible [247]  Sitting in the middle of an armless folding chair, then standing up and sitting down with an arm folded in front of the chest; mean of two trials [36]  Standing all the way up and sitting all the way down 5 times as quickly as possible while keeping the arms folded across the chests; straight back, flat, level, firm seat (45 cm high) [215]  Getting up and sitting from a chair (43 cm high, flat seat), arms crossed over the chest, rising until full extension at trunk and lower limb joints, and returning with the back fully supported at the back of the chair; best of two trials [136]  Standard chair (43.2 cm high), transferring to a standing position and returning to a sitting position, not allowed to use arms [277]  Standard padded chair (43.2 cm high) without armrests, both arms crossed against the chest, starting from a seated position and standing up (legs straight) and sitting down (full weight on the chair) [278]  Getting up from and sitting down on the chair (43.6 cm high) without arm rests [137]  Standing and sitting five times from an armless chair (46 cm high), not permitted to use arms [125]  Straight-back chair, placed against a wall, with a hard seat and standard height, sitting with the feet on the floor and arms folded across the chests on the chair, time measured at the final standing position [2]  Standing up and sitting down as quickly as possible five times in a row from an armless straight-back chair, arms across the chest, time measured at the final standing position [5]; time measured at the final sitting position [3]  Sitting in a hard-backed chair (43 cm high), arms folded across the chest, rising as fast as possible to a full standing position, then returning to a full-sitting position five times [93, 94]  Rising fully from a standard armless, backless chair five times as fast as possible, arms folded closely to the trunk, no moving of the feet during the test, time measured at the final sitting position [38]  Standing up from a straight-backed chair (43 cm high) five times at a self-selected pace, arms folded across the chest [53]  Sitting on a chair with the back touching the backrest, seat height adjusted to participant’s lower leg length, knees flexed at 90°, time measured at the final sitting position [279]  Standing up and sitting down five times as quickly as possible from a straight-backed chair [280]; time measured at the final standing position [23]  Standard chair with arm rests, both arms crossed against the chest, starting from a seated position (upper back against seat), standing up to full extension and sitting down again (upper back against seat), best of two trials [118]  N/A [6, 60, 81, 85, 91, 109, 141, 142, 145, 149, 232, 260, 261, 266, 281-283] | Time (s) [1-3, 5, 6, 9, 23, 27, 34, 36, 41, 53, 60, 63, 70, 81, 85, 90, 91, 93-95, 109, 112, 118, 124, 125, 127, 136, 137, 141, 142, 145, 147, 149, 168, 188, 203, 215, 232, 240, 246, 247, 249, 260, 261, 266, 274, 275, 277-284]  Stands/min (60*[5/time, s]) [7]  Score (0-4) [28] | R | 1 | 81289 | | 40-90+  (58.7-71.0) | 41301 F, 36656 M |
| One time sit-to-stand  (7 studies) | Sitting in a straight-back chair, barefooted, on cue, standing up and sitting down as quickly as possible, upper extremity use not permitted [21]  Sitting on a chair (43 cm high), on cue, rising to full stance; best of three trials [285]  Adjusted seat height (5 cm increments from 45 to 60 cm) to achieve a 90/90 (hip/knee angle), sitting on the front half of an instrumented chair, using the arms as normally during the task, while standing as quickly as possible, three trials [126]  Chair rise from different seat heights (43 cm, 38 cm, 30 cm) [286]  Standing up as quickly as possible from a standard chair (43 cm high), arms crossed across the chest and feet shoulder-width apart placed flat on the floor [4]  N/A [144, 287] | Time (s) [4, 21, 285-287]  Force (N/s [kg]; W [kg]) [126, 144] | R | 1 | 414 | | 60-74  (61.6-69.9) | 235 F, 79 M |
| Ten times sit-to-stand  (6 studies) | Rising from a sitting to a standing position with straight back and legs and sitting down again as fast as possible [49]  Straight-backed chair (45cm high), arms crossed against the chest, rising as quickly as possible without the use of the hands [215, 250, 251]  Rising from a chair as quickly as possible with arms placed across the chest [229]  N/A [86] | Time (s) [86, 229, 250, 251]  Speed (stands per minute: [10/s]*60) [49] | R | 1 | 3283 | | 50-81  (62.6-69.0) | 1182 F, 1012 M |
| 15 second Sit-to-stand  (1 study) | Straight-backed, non-padded, flat-seated, armless chair, Standing without using hands or arms, arms folded across the chest; mean of two trials [197] | Repetitions | R | 1 | 5777 | | 65-79  (69.8-70.1) | 5777 F |
| 30 second sit-to-stand  (51 studies) | Part of the SFT [42, 45, 57, 154-156, 158-164, 166, 271]; two trials [157]  Part of the Fullerton Functional Fitness Test [170]  Standing in front of a stable chair, hands across the chest, then practicing sitting down and standing up for 30 s [31, 272]  Sitting in a chair (43 cm high) with arms crossed at the wrists and holding against the chest, then standing up as many times as possible [171, 172, 288])  Sitting on a standard armless chair (45 cm high), looking straight forward with arms folded across the chest, then standing up and sitting down as many times as possible [167]  Rising up and sitting down with arms folded in front of the chest as quickly as possible on a firm, armless chair placed against a wall [82, 289]  Standing up and sitting down from a bench without armrests and back support as many times as possible, feet flat on the floor, initial foot placement and chair height individually adjusted [290]  Stand up from a seated position as many times as possible [291]  Stand fully upright and then return to the seated position as many times as possible [66, 67, 87, 173, 174]  Different chair heights (43 cm; then adjusted to 80, 90, 100, 110 and 120% of the participants’ lower leg length), last attempt at the end of 30 s is counted as a full stand if the participant is more than halfway up from sitting [284]  Standard padded chair (43.2 cm high) without armrests, starting from the seated position and standing up (legs straight) and sitting down (full weight on the chair); mean of two trials [278]  Sitting on a chair, back straight, feet shoulder-width apart and flat on the floor, arms crossed at the wrists and held against the chest, then rising to a full stand and returning to a fully seated position as many times as possible [179]  Chair (44 cm high) without arms, sitting in the middle of the chair, feet shoulder width apart and placed on the floor at an angle slightly behind the knees, arms crossed at the wrists and held against the chest, then rising to a full stand and returning back to the initial seated position, as many full stands as possible; mean of two trials [16, 17]  Sitting in the middle of the chair, arms across the chest, then rising to a full stand and returning to a fully seated position as many times as possible [131]  Standard chair with arm rests, both arms crossed against the chest, starting from a seated position (upper back against seat), standing up to full extension and sitting down again (upper back against seat); best of two trials [118]  Sitting in a standard-height chair with arms crossed over the chest, then stand fully and sit down again as many times as possible [97]  N/A [10, 26, 43, 96, 144, 145, 152, 153, 175, 232, 234] | Repetitions | R | 1 | 7493 | | 51-91  (61.1-71.6) | 3730 F, 1697 M |
| 1 minute sit-to-stand  (2 studies) | Stand up from and sit down from a standard height chair without the use of the arms [292]  Sitting on the edge of a standard-height chair, arms crossed over the chest, and repeatedly standing up from and returning to a seated position without assistance [4] | Repetitions | R | 1 | 123 | | 55-70  (62.2-70.7) | 76 F, 47 M |
| One time kneel-to-stand  (1 study) | Part of MOD scale [286] | Score (0-5) | R | 1 | 259 | | 60+  (67.6±7.0) | 143 F, 116 M |
| Floor rise to standing  (6 studies) | Lying in a supine position, with feet together and hands palm down and at the side, then rising to a standing position [93, 94]  Sit and rise from the floor (flat, non-slippery surface), using the minimum support needed [112]  Stand up from a supine position [282]  Sitting and rising unassisted from the floor with partial scores assigned from the two required actions of sitting (5 points) and rising (5 points) and a final composite SRT score [153]  N/A [141] | Time (s)  Score [112, 153] | R | 1 | 172 | | 58-84  (67.0-69.3) | 50 F,49 M |
| Five Step Test  (1 study) | N/A [266] | Time (s) | R | 1 | 621 | | 50+  (66.8-69.4) | 428 F, 193 M |
| Stair climbing  (2 studies) | Walking up and down a standard ﬂight of stairs, three times at self-selected pace, using the handrail for support only if needed [53, 216] | Time (s) | R | 1 | 1143 | | 55-79  (63.8-67.5) | 634 F, 509 M |
| Stair climbing (8 steps)  (2 studies) | Climbing eight steps (17 cm high, 31cm long) without using the handrail, requiring a step by step pattern; best of two trials [136]  N/A [261] | Time (s) | R | 1 | 111 | | 65.6-67.8 | 50 F, 35 M |
| Stair climbing (10 steps)  (3 studies) | Climbing a flight of stairs (10 steps) as quickly as possible without using the handrails or any other aid (14 cm high [87]; 7.8 cm high [89]  Ascending and descending a flight of stairs (10 steps, 0.27 m high and 0.18 m deep) as quickly and safely as possible, while having the option of using a single handrail for support [293] | SCP (W) [87, 89]  Time (s) [293] | R | 1 | 212 | | 50-75  (62.7-71.5) | 152 F, 67 M |
| Stair climbing (11 steps)  (3 studies) | Ascending a standard ﬁght of stairs (11 stairs, 16 cm high), avoiding the use of the handrail [93, 94]; as rapidly as possible [92] | Time (s) [92-94]  SCP (W) [93, 94] | R | 1 | 77 | | 65-84  (68.9-69.3) | 37 F,40 M |
| Stair climbing (12 steps)  (2 studies) | Ascending and descending 12 stairs, permitted to use the handrail, but not allowed to use it to push or pull oneself [277]  Ascending and descending 12 stairs (15cm high, 20 cm tread), as fast as possible while being safe [102] | Time (s) | R | 1 | 337 | | 45-80  (58.7-64.8) | 183 F, 164 M |
| Stair climbing (14 steps)  (1 study) | Walk as fast as possible up 14 stairs without the use of railings [289] | Time (s) | R | 1 | 30 | | 68.5±5.1 | 15 F, 15 M |
| Stair climbing (15 steps)  (1 study) | Ascending and descending a flight of 15 stairs (18 cm high, 27 cm tread) at normal pace, preferably without using the handrail [135] | Time (s) | R | 1 | 134 | | 69.6-70.3 | 85 F, 49 M |
| Stair ascent (23 steps)  (1 study) | Walking up one flight of stairs consisting of 23 steps (16.5 cm high, 19.2 cm wide) as quickly as possible; after 14 steps, the participants make a left-hand wrap-around turn and then completed the remaining nine steps; not allowed to use the handrails; best of the two trials [294] | Time (s) | R | 1 | 62 | | 60-83  (66.6-71.0) | N/A |
| Stair ascent (16 steps)  (1 study) | 16 steps, height of 15 cm; not allowed to hold the handrails [143] | Time (s) | R | 1 | 48 | | 60-80  (68.6±6.1) | N/A |
| Stair ascent (10 steps)  (4 studies) | Ascending a 10-stair prop (17 cm high, 30 cm deep) at fast pace [168]  Walking up 10 steps in an expeditious and safe manner, placing one hand close to the handrail for balance if necessary, but not on the handrail [275]  Ascending a 10-stair flight (16.5 cm stair high) as fast as possible, use of handrail allowed [70]  Climbing 10 steps as fast as comfortably possible with one hand near, but not on, the handrail [200] | Time (s) | R | 1 | 158 | | 62-80  (66.0-70.0) | 69 F, 35 M |
| Stair ascent (9 steps)  (2 studies) | Walking quickly but safely up and down a nine step flight of stairs (step height: 17 cm); time started after the cue to go and stopped when the second foot reached the top step [147, 283] | Time (s) | R | 1 | 71 | | 62.7-70.0 | 46 F, 25 M |
| Stair ascent (4 steps)  (1 study) | Walking up 4 stairs (15 cm high), arriving on a full stance on the fourth step without any support or help, three trials, best score [285] | Time (s) | R | 1 | 33 | | 60-74  (64.4-65.7) | 21 F, 12 M |
| Stair ascent (one time)  (1 study) | Part of MOD scale [286] | Score (0-5) | R | 1 | 259 | | 60+  (67.6±7.0) | 143 F, 116 M |
| Stair descent (16 steps)  (1 study) | 16 steps, height of 15 cm; not allowed to hold the handrails [143] | Time (s) | R | 1 | 48 | | 60-80  (68.6±6.1) | N/A |
| Stair descent (14 steps)  (1 study) | 14 steps (height 17 cm, length 30 cm); time starts with participants’ initiation of first step and stops when both feet are on the landing [282] | Time (s) | R | 1 | 33 | | 67±4.5 | N/A |
| Stair descent (10 steps)  (1 study) | Walking down 10 steps in an expeditious and safe manner, placing one hand close to the handrail for balance if necessary, but not on the handrail [275] | Time (s) | R | 1 | 19 | | 66.0±1.0 | 14 F, 5 M |
| Stair descent (9 steps)  (1 study) | Walking quickly but safely up and down a nine step flight of stairs (step height: 17 cm); time started after the cue to go and stopped when the second foot reached the floor [147] | Time (s) | R | 1 | 48 | | 69.8-70.0 | 26 F, 22 M |
| Stair descent (one time)  (1 study) | Part of MOD scale [286] | Score (0-5) | R | 1 | 259 | | 60+  (67.6±7.0) | 143 F, 116 M |
| Functional leg extensor strength  (1 study) | Taking a short step forward, ﬁrst with the right leg, squat down until the knee of the tracking leg lightly touches the mat, and then rise up immediately and step back to the starting position, then repeating with the left leg [53] | Maximal weight relative to the subject’s body weight | R | 1 | 1133 | | 55-79  (63.8-64.1) | 632 F, 501 M |
| Lift and reach (one minute)  (2 studies) | Sitting at a standard height desk, then repeatedly lifting a weight onto and off a shelf placed on the desk located at shoulder level immediately in front (10 pound for women, 20 pound for men) [292]  Sitting in a standard chair at a standard height desk (75 cm), then lifting a weight repeatedly onto and off a shelf positioned at approximate shoulder height, 37 cm above the desktop (5 kg dumbbell for women, 8 kg dumbbell for men) [4] | Repetitions | R | 1 | 123 | | 55-70  (62.6-70.7) | 76 F, 47M |
| Standing long jump  (2 studies) | Jumping horizontally, using a 2-ft. takeoff and landing, three trials, measured at the heel of the foot [32]  Jumping with no restriction to arm movements as high and as fast without bending legs in air and landing with both feet on the jump mat; average of three trials [263] | Distance (cm [32]; m [263])  Power (kg/body mass), Velocity (m/s) [263] | R | 1 | 98 | | 50-79  (63.7±1.1) | 54 F, 44 M |
| Squat jump  (1 study) | Static position, knee bent in a 90° angle, hands on the hip during the whole jump; three maximal trials separated by 1.5 min of rest [34] | Maximal Ground Reaction Force (A; N*kg-1), Rate of Force Development (N*kg-1), Force (N) | R | 1 | 63 | | 65-70  (67.5±0.4) | 63 F |
| Single knee extension contractions  (1 study) | Single knee extension contractions with a handgrip device at 20%, 40%, and 60% of the subject's max voluntary contraction [273] | Maximum work rate (WR_max_) | R | 3 | 32 | | 59-85  (66.0±2.0) | 12 F, 19 M |

| **Test characteristics** | | | **Scale** | | **Study population** | | |
| --- | --- | --- | --- | --- | --- | --- | --- |
| **Strength test** | **Description / Variation** | **Unit** | **Level** | **Items** | **N^a^** | **Age** | **Sex^b^** |
| **One repetition maximum** | | | | | | | |
| Handgrip strength  (81 studies) | Both hands, best trial [228]  Bi-handgrip strength, two trials [105]  Standing and then grasping a grip device; best of three trials [31]; mean of two trials [147]; best of two trials [229]  Sitting, elbow fully extended in front on shoulder height; mean of three trials [230]  Sitting position, shoulders adducted, neutrally rotated, elbow flexed at 90°, forearm neutral   - dominant hand; best of three trials [231] - both hands, three trials for each hand, best score [121]   Electronic / hydraulic dynamometer   - best trial (number not specified) [9, 40] - two trials [199] - three trials [82]; mean of three trials [196] - best of three trials [49, 57, 146, 184, 188, 232, 233] - both arms, mean of six trials [124] - standing; mean of three trials [234] - Sitting, shoulders adducted, neutrally rotated, elbow flexed at 90°, forearm neutral; mean of two trials [235]; mean of three trials [236] - dominant hand [176]; arm by side; best of three trials [168, 237] - dominant hand, sitting in an upright position, arm of the measured hand unsupported and parallel to the body; one trial [70] - dominant hand, sitting, dominant shoulder in rest position, elbow flexed 90° without support, forearm and wrist at neutral position; best of three trials [201] - dominant hand, sitting comfortably, dominant arm by side, elbow flexed 90°, hand held in mid-supination/pronation position; best of three trials [180] - both hands, one trial in each hand; best score [63] - both hands; three trials in each hand, best score for each hand [238]; best trial of the dominant hand [239] - best score of both hands [203]; best score of each hand [240]; sum of best score of each hand [111]; mean score of each hand [241] - both hands, wrist in neutral position, elbow flexed at 90°; three trials for each hand; mean of each hand [242] - both hands, two trials for each hand, mean for each hand and larger mean from one of the hands [207] - N/A [28, 91]   Bulb hand dynamometer   - both hands, holding at shoulder level, two trials in each hand; mean of both hands added [243] - dominant hand, medium (women) or large (men) dynamometer, sitting; best of three trials [71] - both hands, three trials in each hand; mean for each hand [244] - both hands, two trials in each hand; mean for each hand [245]   Calibrated dynamometer   - both hands, elbow flexed to 90° [167] - preferred hand, arm raised overhead then slowly lowered towards floor [21]; three trials [32]   Handheld dynamometer   - both hands, standing, best of two trials (each hand) [2, 59, 69, 128]; mean of three trials (each hand) [110] - both hands, two trials on the dominant and three trials on each hand [246] - two trials; mean score [197]; best score [120, 247] - dominant hand [179]; mean of two trials [248]; mean of three trials [249]; best of two trials [133] - sitting, elbow flexed two 90°, best of two trials [204]; best of three trials [250, 251] - standing with their forearms bent at 90 °, best of two trials [7] - elbow positioned at 90° of the side of the body; dominant hand; mean of three trials [252]   N/A [48, 103, 139, 150, 152, 165, 214, 216, 217, 253-261] | Force (kg) [2, 9, 31, 40, 49, 57, 69, 70, 105, 111, 128, 139, 167, 180, 184, 188, 197, 199, 201, 203, 207, 228, 231, 235, 237-243, 245-251, 253] [59, 179, 203, 204, 233, 234] [7, 32, 82, 91, 120, 121, 124, 133, 147, 150, 152, 165, 176, 214, 216, 217, 229, 230, 240, 252, 254-261]  Force (pounds) [21]  Force (kg)/bodyweight (kg) [168]  % of people with force scores (kg, classified as weakness) [196]  kg/cm^2^ [71]  Force (pound per square) [244]  Force (kPa) [28]  Force (Newton) [63] Quartiles (% lower quartile reported) [236] | R | 1-2 | 130821 | 34-89  (60.4-70.5) | 75538 F, 49439 M |
| Shoulder flexor strength  (1 study) | Right arm, 90° shoulder flexion, elbow in full extension; mean of three trials [47] | Force (kg) | R | 1 | 85 | 65-84  (69.0±0.4) | 37 F, 48 M |
| Hip muscle strength  (2 studies) | Supine on a plinth, both legs 10° abducted, a strap (5 cm wide) around the plinth and over the pelvis; for the examiner-resisted test, participants pushed as hard as possible against the HHD as the examiner provided resistance, stabilizing and positioning the HHD; for the belt-resisted test, HHD is placed between the side of the test leg and a second strap (5cm width), participants spread their legs apart simultaneously as hard as possible [262] Right, and left hip abduction strength on isotonic external resistance machine [263] | Torques  kg/body mass [263] | R | 2 | 45 | 55-75  (63.7-68.4) | 31 F, 14 M |
| Knee extensor strength  (1 study) | Computer-based manual muscle testing, knee at 30° flexion; mean of three trials [47] | Force (kg) | R | 1 | 85 | 65-84  (69.0±0.4) | 37 F, 48 M |
| Leg strength  (6 studies) | Sitting in a standard chair (45 cm high), connected to a WBB (57° angle from the ground) via custom seatbelt straps; pressing the feet on the WBB as hard and as fast as possible; three trials [264]  Dynametry [254]  Leg press machine [34, 145, 176, 263] | Force (kg)  Kg/body mass [263] | R | 1 | 272 | 55-75  (61.1-69.3) | 140 F, 76 M |
| Toe grasping strength  (2 studies) | Barefoot, one-leg stand, both hands on the wall in front, holding the dynamometer grasping bar with the toes [265]  Sitting upright on a chair, without leaning on the backrest; both hips and knees flexed at approximately 90°; ankles placed in a neutral position and fixed with a strap [107] | Force (kg) | R | 1 | 7227 | 52-78  (66.3-67.6) | 534 F, 188 M |
| **Maximal Isometric Strength (MIS)** | | | | | | | |
| Elbow extensor strength  (1 study) | Instrumented wooden pole that subjects pressed against the ground; Subjects sit on a bench, with the shoulder in a neutral position, elbow angle of 90°, forearm was parallel to the ground; best of three trials [146] | MVC (kg) | R | 1 | 26 | 69.2-70.0 | 17 F, 9 M |
| Hip extensor strength  (1 study) | HHD, mean score [178] | % (strength/body weight) | R | 1 | 39 | 60-78  (68.5-69.7) | 15 F, 24 M |
| Hip flexor strength  (2 studies) | HHD [91], mean score [178]  HHD, sitting position, hip flexed at 90° and knee flexed at 90°; sensor of the HHD was applied to a distal site on the anterior surface of the thigh [55] | % (strength/body weight)  Force (kg) [55, 91] | R | 1 | 818 | 60-78  (68.5-69.7) | 775 F, 313 M |
| Hip abductor strength  (2 studies) | Supine position with the hip and knee fully extended and hip positioned in neutral abduction; sensor of the HHD was applied 5 cm proximal from the lateral malleolus [55]  HHD, three trials (each hold for 3-5 s) [101] | Force (kg) | R | 1 | 744 | 61.8-68.7 | 482 F, 262 M |
| Knee extensor strength  (11 studies) | HHD, sitting upright, raising lower legs up 90°, parallel to the ground, holding this position as strongly as possible against the maximum persistent (5 s) force applied by the examiner through the HHD placed on the front of the ankle proximal to the medial malleolus; two trials for each leg, best score [266]  HHD [91, 107]; mean score [10, 178]  Leaning back in a chair, extending both legs at the knee while pulling against a dynamometer; best of two trials [66]  Sitting on a high chair and pushing against a strap linked to a spring gauge [100]  Seated position using an adjustable chair with a 90° angle of hip and knee joints, dominant leg, as fast as forcefully, strongest of five trials [34]  Dynamometer, right side; sitting on a backwardly-inclined (5°) chair, range of motion was set from a knee joint angle of 90° to 160° (180° represents full extension); best of two trials [145]  Sitting in an upright position with back support and with both the hip and knee flexed at 70°; distal leg affixed to a strain gauge force transducer; best of three trials [146]  Dominant limb [232] | Force (kg) [34, 66, 107, 232, 266]  % (strength/body weight) [100, 178]  N/kg [10]  Peak torque (Nm) [145, 232]  MVC (kg) [146] | R | 1-2 | 1595 | 60-78  (61.1-71.6) | 1038 F, 402 M |
| Knee flexor strength  (1 study) | HHD, mean score [178] | % (strength/body weight) | R | 1 | 39 | 60-78  (68.5-69.7) | 15 F, 24 M |
| Leg strength  (6 studies) | Dynamometer, both legs simultaneously [267]; mean of two trials [245]; best of two trials [268]  Dynamometer, both legs simultaneously, standing with back straight against a wall and knees 115° flexed; a bar connected by a chain to the dynamometer was held in front of the thighs and has to be lifted upwards with maximum force using only the legs, and keeping the neck and back straight; mean of two trials [243, 269]  Fitted with the harness around their hips and seated in a standard chair (45 cm height) with the seatbelt straps connecting the harness to the FysioMeter-mount. The lengths of the seatbelt straps were adjusted using a tape measure and a goniometer angle between sessions to reach a knee angle of approximately 120°; foot placed in the middle of the Wii Balance Board [270] | Force (kg) | R | 1 | 2544 | 50-79  (61.4-69.0) | 1230 F, 1277 M |
| Ankle dorsiflexor strength  (7 studies) | HHD [91, 149]; mean score [10, 178]; mean of three trials [90, 112]; three trials (each for 3-5 s) [101] | % (strength/body weight) [178]  N/kg [10]  Force (kg) [90, 91, 101, 112, 149] | R | 1 | 357 | 60-78  (61.8-69.7) | 222 F, 135 M |
| Ankle plantar flexor strength  (5 studies) | HHD [91, 142, 149]; mean of three trials [90, 112]; three trials (each for 3-5 s) [101] | Force (kg) | R | 1 | 832 | 50-80  (61.8-68.5) | 450 F, 392 M |
| **Functional muscle power** | | | | | | | |
| *Upper body functional muscle power* | | | | | | | |
| 30 second arm curl  (20 studies) | Part of the SFT [42, 45, 154, 156, 160, 162-164, 166, 170, 271]  Performing as many biceps curls as possible in 30 s, using a 2.27-kg dumbbell (full range of motion; study in women) [169, 272]  Flexing and extending the elbow of the dominant hand, lifting a weight (8 lb [3629g] dumbbell for men; 5lb dumbbell [2268g] for women) through the complete range of motion as many times as possible in 30 s [173]  Sitting on a chair, using the dominant hand to bring a weight (2.0 kg) up and down (flex and extend the biceps) as many times as possible in 30 s [179]  Hand curling a hand weight (5 pounds for women and 8 pounds for men) for 30 s [131]  Siting on the chair, holding the dumbbell (women 5 lbs, men 8 lbs) in the hand with palm facing towards the body (with the arm in a vertically down position beside the chair), bracing the upper arm against the body so that only the lower arm is moving, then curling the arm up through a full range of motion, gradually performing elbow flexion with supination; as the arm is lowered through the full range of motion, gradually return to the starting position [152]  N/A [144, 175] | Repetitions | R | 1 | 5768 | 51-89  (61.9.0-69.9) | 2652 F, 1111 M |
| Abdominal Strength  (2 studies) | Lying down on an abdominal pad, with knees flexed at 90°, hands set on the pad frame. Rise with the chest up to approximately 30° from the floor as many times as possible in 30 s [167]  Lying on sit-up equipment and performing sit-ups with the feet attached to the equipment’s foot holders as many times as possible in 30 s [235] | Repetitions | R | 1 | 252 | 59-60+  (63.0-66.9) | 230 F, 122 M |
| Single forearm contractions  (1 study) | Dynamic single contractions in both arms, HHD at 10%, 20%, and 40% of the subject's maximum voluntary contraction [273] | MVC (kg) | R | 1 | 32 | 59-85  (66.0±2) | 13 F, 19 M |
| Seated medicine ball throw  (1 study) | Participants horizontally propel a 3 kg ball without trunk flexion [144] | Distance (m) | R | 1 | 36 | 68.8-68.9 | 20 F, 16 M |
| *Lower body functional muscle power* | | | | | | | |
| Five times Sit-to-Stand  (61 studies) | Part of the SPPB [27, 28]  Rising from a chair and sitting back down five times [9, 34, 112, 168]; without arm support [147, 240, 274, 275]; time measured at the final sitting down, best of three trials [127]; mean of three trials [90]  Five repetitive chair stands as quickly as possible with arms folded across the chest [1, 7, 41, 63, 70, 203, 246, 249]); mean of two trials [8, 276]; mean of three trials [95, 188]  Sitting in a standard chair, arms folded across the chest, standing up and sitting down five times [124]; as fast as possible [247]  Sitting in the middle of an armless folding chair, then standing up and sitting down with an arm folded in front of the chest; mean of two trials [36]  Standing all the way up and sitting all the way down 5 times as quickly as possible while keeping the arms folded across the chests; straight back, flat, level, firm seat (45 cm high) [215]  Getting up and sitting from a chair (43 cm high, flat seat), arms crossed over the chest, rising until full extension at trunk and lower limb joints, and returning with the back fully supported at the back of the chair; best of two trials [136]  Standard chair (43.2 cm high), transferring to a standing position and returning to a sitting position, not allowed to use arms [277]  Standard padded chair (43.2 cm high) without armrests, both arms crossed against the chest, starting from a seated position and standing up (legs straight) and sitting down (full weight on the chair) [278]  Getting up from and sitting down on the chair (43.6 cm high) without arm rests [137]  Standing and sitting five times from an armless chair (46 cm high), not permitted to use arms [125]  Straight-back chair, placed against a wall, with a hard seat and standard height, sitting with the feet on the floor and arms folded across the chests on the chair, time measured at the final standing position [2]  Standing up and sitting down as quickly as possible five times in a row from an armless straight-back chair, arms across the chest, time measured at the final standing position [5]; time measured at the final sitting position [3]  Sitting in a hard-backed chair (43 cm high), arms folded across the chest, rising as fast as possible to a full standing position, then returning to a full-sitting position five times [93, 94]  Rising fully from a standard armless, backless chair five times as fast as possible, arms folded closely to the trunk, no moving of the feet during the test, time measured at the final sitting position [38]  Standing up from a straight-backed chair (43 cm high) five times at a self-selected pace, arms folded across the chest [53]  Sitting on a chair with the back touching the backrest, seat height adjusted to participant’s lower leg length, knees flexed at 90°, time measured at the final sitting position [279]  Standing up and sitting down five times as quickly as possible from a straight-backed chair [280]; time measured at the final standing position [23]  Standard chair with arm rests, both arms crossed against the chest, starting from a seated position (upper back against seat), standing up to full extension and sitting down again (upper back against seat), best of two trials [118]  N/A [6, 60, 81, 85, 91, 109, 141, 142, 145, 149, 232, 260, 261, 266, 281-283] | Time (s) [1-3, 5, 6, 9, 23, 27, 34, 36, 41, 53, 60, 63, 70, 81, 85, 90, 91, 93-95, 109, 112, 118, 124, 125, 127, 136, 137, 141, 142, 145, 147, 149, 168, 188, 203, 215, 232, 240, 246, 247, 249, 260, 261, 266, 274, 275, 277-284]  Stands/min (60*[5/time, s]) [7]  Score (0-4) [28] | R | 1 | 81289 | 40-90+  (58.7-71.0) | 41301 F, 36656 M |
| One time sit-to-stand  (7 studies) | Sitting in a straight-back chair, barefooted, on cue, standing up and sitting down as quickly as possible, upper extremity use not permitted [21]  Sitting on a chair (43 cm high), on cue, rising to full stance; best of three trials [285]  Adjusted seat height (5 cm increments from 45 to 60 cm) to achieve a 90/90 (hip/knee angle), sitting on the front half of an instrumented chair, using the arms as normally during the task, while standing as quickly as possible, three trials [126]  Chair rise from different seat heights (43 cm, 38 cm, 30 cm) [286]  Standing up as quickly as possible from a standard chair (43 cm high), arms crossed across the chest and feet shoulder-width apart placed flat on the floor [4]  N/A [144, 287] | Time (s) [4, 21, 285-287]  Force (N/s [kg]; W [kg]) [126, 144] | R | 1 | 414 | 60-74  (61.6-69.9) | 235 F, 79 M |
| Ten times sit-to-stand  (6 studies) | Rising from a sitting to a standing position with straight back and legs and sitting down again as fast as possible [49]  Straight-backed chair (45cm high), arms crossed against the chest, rising as quickly as possible without the use of the hands [215, 250, 251]  Rising from a chair as quickly as possible with arms placed across the chest [229]  N/A [86] | Time (s) [86, 229, 250, 251]  Speed (stands per minute: [10/s]*60) [49] | R | 1 | 3283 | 50-81  (62.6-69.0) | 1182 F, 1012 M |
| 15 second Sit-to-stand  (1 study) | Straight-backed, non-padded, flat-seated, armless chair, Standing without using hands or arms, arms folded across the chest; mean of two trials [197] | Repetitions | R | 1 | 5777 | 65-79  (69.8-70.1) | 5777 F |
| 30 second sit-to-stand  (51 studies) | Part of the SFT [42, 45, 57, 154-156, 158-164, 166, 271]; two trials [157]  Part of the Fullerton Functional Fitness Test [170]  Standing in front of a stable chair, hands across the chest, then practicing sitting down and standing up for 30 s [31, 272]  Sitting in a chair (43 cm high) with arms crossed at the wrists and holding against the chest, then standing up as many times as possible [171, 172, 288])  Sitting on a standard armless chair (45 cm high), looking straight forward with arms folded across the chest, then standing up and sitting down as many times as possible [167]  Rising up and sitting down with arms folded in front of the chest as quickly as possible on a firm, armless chair placed against a wall [82, 289]  Standing up and sitting down from a bench without armrests and back support as many times as possible, feet flat on the floor, initial foot placement and chair height individually adjusted [290]  Stand up from a seated position as many times as possible [291]  Stand fully upright and then return to the seated position as many times as possible [66, 67, 87, 173, 174]  Different chair heights (43 cm; then adjusted to 80, 90, 100, 110 and 120% of the participants’ lower leg length), last attempt at the end of 30 s is counted as a full stand if the participant is more than halfway up from sitting [284]  Standard padded chair (43.2 cm high) without armrests, starting from the seated position and standing up (legs straight) and sitting down (full weight on the chair); mean of two trials [278]  Sitting on a chair, back straight, feet shoulder-width apart and flat on the floor, arms crossed at the wrists and held against the chest, then rising to a full stand and returning to a fully seated position as many times as possible [179]  Chair (44 cm high) without arms, sitting in the middle of the chair, feet shoulder width apart and placed on the floor at an angle slightly behind the knees, arms crossed at the wrists and held against the chest, then rising to a full stand and returning back to the initial seated position, as many full stands as possible; mean of two trials [16, 17]  Sitting in the middle of the chair, arms across the chest, then rising to a full stand and returning to a fully seated position as many times as possible [131]  Standard chair with arm rests, both arms crossed against the chest, starting from a seated position (upper back against seat), standing up to full extension and sitting down again (upper back against seat); best of two trials [118]  Sitting in a standard-height chair with arms crossed over the chest, then stand fully and sit down again as many times as possible [97]  N/A [10, 26, 43, 96, 144, 145, 152, 153, 175, 232, 234] | Repetitions | R | 1 | 7493 | 51-91  (61.1-71.6) | 3730 F, 1697 M |
| 1 minute sit-to-stand  (2 studies) | Stand up from and sit down from a standard height chair without the use of the arms [292]  Sitting on the edge of a standard-height chair, arms crossed over the chest, and repeatedly standing up from and returning to a seated position without assistance [4] | Repetitions | R | 1 | 123 | 55-70  (62.2-70.7) | 76 F, 47 M |
| One time kneel-to-stand  (1 study) | Part of MOD scale [286] | Score (0-5) | R | 1 | 259 | 60+  (67.6±7.0) | 143 F, 116 M |
| Floor rise to standing  (6 studies) | Lying in a supine position, with feet together and hands palm down and at the side, then rising to a standing position [93, 94]  Sit and rise from the floor (flat, non-slippery surface), using the minimum support needed [112]  Stand up from a supine position [282]  Sitting and rising unassisted from the floor with partial scores assigned from the two required actions of sitting (5 points) and rising (5 points) and a final composite SRT score [153]  N/A [141] | Time (s)  Score [112, 153] | R | 1 | 172 | 58-84  (67.0-69.3) | 50 F,49 M |
| Five Step Test  (1 study) | N/A [266] | Time (s) | R | 1 | 621 | 50+  (66.8-69.4) | 428 F, 193 M |
| Stair climbing  (2 studies) | Walking up and down a standard ﬂight of stairs, three times at self-selected pace, using the handrail for support only if needed [53, 216] | Time (s) | R | 1 | 1143 | 55-79  (63.8-67.5) | 634 F, 509 M |
| Stair climbing (8 steps)  (2 studies) | Climbing eight steps (17 cm high, 31cm long) without using the handrail, requiring a step by step pattern; best of two trials [136]  N/A [261] | Time (s) | R | 1 | 111 | 65.6-67.8 | 50 F, 35 M |
| Stair climbing (10 steps)  (3 studies) | Climbing a flight of stairs (10 steps) as quickly as possible without using the handrails or any other aid (14 cm high [87]; 7.8 cm high [89]  Ascending and descending a flight of stairs (10 steps, 0.27 m high and 0.18 m deep) as quickly and safely as possible, while having the option of using a single handrail for support [293] | SCP (W) [87, 89]  Time (s) [293] | R | 1 | 212 | 50-75  (62.7-71.5) | 152 F, 67 M |
| Stair climbing (11 steps)  (3 studies) | Ascending a standard ﬁght of stairs (11 stairs, 16 cm high), avoiding the use of the handrail [93, 94]; as rapidly as possible [92] | Time (s) [92-94]  SCP (W) [93, 94] | R | 1 | 77 | 65-84  (68.9-69.3) | 37 F,40 M |
| Stair climbing (12 steps)  (2 studies) | Ascending and descending 12 stairs, permitted to use the handrail, but not allowed to use it to push or pull oneself [277]  Ascending and descending 12 stairs (15cm high, 20 cm tread), as fast as possible while being safe [102] | Time (s) | R | 1 | 337 | 45-80  (58.7-64.8) | 183 F, 164 M |
| Stair climbing (14 steps)  (1 study) | Walk as fast as possible up 14 stairs without the use of railings [289] | Time (s) | R | 1 | 30 | 68.5±5.1 | 15 F, 15 M |
| Stair climbing (15 steps)  (1 study) | Ascending and descending a flight of 15 stairs (18 cm high, 27 cm tread) at normal pace, preferably without using the handrail [135] | Time (s) | R | 1 | 134 | 69.6-70.3 | 85 F, 49 M |
| Stair ascent (23 steps)  (1 study) | Walking up one flight of stairs consisting of 23 steps (16.5 cm high, 19.2 cm wide) as quickly as possible; after 14 steps, the participants make a left-hand wrap-around turn and then completed the remaining nine steps; not allowed to use the handrails; best of the two trials [294] | Time (s) | R | 1 | 62 | 60-83  (66.6-71.0) | N/A |
| Stair ascent (16 steps)  (1 study) | 16 steps, height of 15 cm; not allowed to hold the handrails [143] | Time (s) | R | 1 | 48 | 60-80  (68.6±6.1) | N/A |
| Stair ascent (10 steps)  (4 studies) | Ascending a 10-stair prop (17 cm high, 30 cm deep) at fast pace [168]  Walking up 10 steps in an expeditious and safe manner, placing one hand close to the handrail for balance if necessary, but not on the handrail [275]  Ascending a 10-stair flight (16.5 cm stair high) as fast as possible, use of handrail allowed [70]  Climbing 10 steps as fast as comfortably possible with one hand near, but not on, the handrail [200] | Time (s) | R | 1 | 158 | 62-80  (66.0-70.0) | 69 F, 35 M |
| Stair ascent (9 steps)  (2 studies) | Walking quickly but safely up and down a nine step flight of stairs (step height: 17 cm); time started after the cue to go and stopped when the second foot reached the top step [147, 283] | Time (s) | R | 1 | 71 | 62.7-70.0 | 46 F, 25 M |
| Stair ascent (4 steps)  (1 study) | Walking up 4 stairs (15 cm high), arriving on a full stance on the fourth step without any support or help, three trials, best score [285] | Time (s) | R | 1 | 33 | 60-74  (64.4-65.7) | 21 F, 12 M |
| Stair ascent (one time)  (1 study) | Part of MOD scale [286] | Score (0-5) | R | 1 | 259 | 60+  (67.6±7.0) | 143 F, 116 M |
| Stair descent (16 steps)  (1 study) | 16 steps, height of 15 cm; not allowed to hold the handrails [143] | Time (s) | R | 1 | 48 | 60-80  (68.6±6.1) | N/A |
| Stair descent (14 steps)  (1 study) | 14 steps (height 17 cm, length 30 cm); time starts with participants’ initiation of first step and stops when both feet are on the landing [282] | Time (s) | R | 1 | 33 | 67±4.5 | N/A |
| Stair descent (10 steps)  (1 study) | Walking down 10 steps in an expeditious and safe manner, placing one hand close to the handrail for balance if necessary, but not on the handrail [275] | Time (s) | R | 1 | 19 | 66.0±1.0 | 14 F, 5 M |
| Stair descent (9 steps)  (1 study) | Walking quickly but safely up and down a nine step flight of stairs (step height: 17 cm); time started after the cue to go and stopped when the second foot reached the floor [147] | Time (s) | R | 1 | 48 | 69.8-70.0 | 26 F, 22 M |
| Stair descent (one time)  (1 study) | Part of MOD scale [286] | Score (0-5) | R | 1 | 259 | 60+  (67.6±7.0) | 143 F, 116 M |
| Functional leg extensor strength  (1 study) | Taking a short step forward, ﬁrst with the right leg, squat down until the knee of the tracking leg lightly touches the mat, and then rise up immediately and step back to the starting position, then repeating with the left leg [53] | Maximal weight relative to the subject’s body weight | R | 1 | 1133 | 55-79  (63.8-64.1) | 632 F, 501 M |
| Lift and reach (one minute)  (2 studies) | Sitting at a standard height desk, then repeatedly lifting a weight onto and off a shelf placed on the desk located at shoulder level immediately in front (10 pound for women, 20 pound for men) [292]  Sitting in a standard chair at a standard height desk (75 cm), then lifting a weight repeatedly onto and off a shelf positioned at approximate shoulder height, 37 cm above the desktop (5 kg dumbbell for women, 8 kg dumbbell for men) [4] | Repetitions | R | 1 | 123 | 55-70  (62.6-70.7) | 76 F, 47M |
| Standing long jump  (2 studies) | Jumping horizontally, using a 2-ft. takeoff and landing, three trials, measured at the heel of the foot [32]  Jumping with no restriction to arm movements as high and as fast without bending legs in air and landing with both feet on the jump mat; average of three trials [263] | Distance (cm [32]; m [263])  Power (kg/body mass), Velocity (m/s) [263] | R | 1 | 98 | 50-79  (63.7±1.1) | 54 F, 44 M |
| Squat jump  (1 study) | Static position, knee bent in a 90° angle, hands on the hip during the whole jump; three maximal trials separated by 1.5 min of rest [34] | Maximal Ground Reaction Force (A; N*kg-1), Rate of Force Development (N*kg-1), Force (N) | R | 1 | 63 | 65-70  (67.5±0.4) | 63 F |
| Single knee extension contractions  (1 study) | Single knee extension contractions with a handgrip device at 20%, 40%, and 60% of the subject's max voluntary contraction [273] | Maximum work rate (WR_max_) | R | 3 | 32 | 59-85  (66.0±2.0) | 12 F, 19 M |

^a^ ^a^The total number included was the total number of participants in all studies per balance test; ^b^M: Male; F: Female; (sex was not reported in all papers, and the number will differ from total included in ^a^); OLS: One-leg standing balance; SEBT: Star Excursion Balance Test; TUG: Timed Up and Go; FRT: Functional Reach Test; LRT: Lateral Reach Test; SOT: Sensory Organization Test; BBS: Berg Balance Scale; SPPB: Short Physical Performance Battery; PPT: Physical Performance Test; mPPT: modified Physical Performance Test; FAB: Fullerton Advanced Balance; CS-PFP-10: Continuous Scale-Physical Functional Performance-10 item test; PPB: Physical Performance Battery; CBM: Community Balance & Mobility scale; FFM : Functional Movement Measurement; N: Nominal; O: Ordinal; R: Ratio; RMS: Root Mean Square; MPF: Mean Power Frequency; CoP: Center of Pressure; CoG: Center of Gravity; MSL: Maximum Step Length; ft: feet; max: maximum; s: seconds; rep: repetitions; %: percentage; cm: centimeter; HHD: Hand Held Dynamometer; WBB: Wii Balance Board; SFT: Senior Fitness Test; MVC: Maximum Voluntary Contraction; MOD scale: Modification scale; SCP: Stair Climbing Power; n: number of participants; N/A: not applicable.

**References**

1. Al Snih S, Kaushik V, Eschbach K, Markides K: **Ethnic differences in physical performance in older Americans: data from the Third National Health and Nutrition Examination Survey (1988-1994)**. *Aging-Clinical & Experimental Research* 2008, **20**(2):139-144.

2. Keevil VL, Hayat S, Dalzell N, Moore S, Bhaniani A, Luben R, Wareham NJ, Khaw KT: **The physical capability of community-based men and women from a British cohort: the European Prospective Investigation into Cancer (EPIC)-Norfolk study**. *BMC Geriatrics* 2013, **13**:93.

3. Nickel KJ, Acree LS, Montgomery PS, Gardner AW: **Association between lower-extremity function and arterial compliance in older adults**. *Angiology* 2008, **59**(2):203-208.

4. Ritchie C, Trost SG, Brown W, Armit C: **Reliability and validity of physical fitness field tests for adults aged 55 to 70 years**. *Journal of Science & Medicine in Sport* 2005, **8**(1):61-70.

5. Sharkey JR, Ory MG, Branch LG: **Severe elder obesity and 1-year diminished lower extremity physical performance in homebound older adults**. *Journal of the American Geriatrics Society* 2006, **54**(9):1407-1413.

6. Tiedemann A, O'Rourke S, Sesto R, Sherrington C: **A 12-week Iyengar yoga program improved balance and mobility in older community-dwelling people: a pilot randomized controlled trial**. *Journals of Gerontology Series A-Biological Sciences & Medical Sciences* 2013, **68**(9):1068-1075.

7. Keevil VL, Luben R, Hayat S, Sayer AA, Wareham NJ, Khaw K-T: **Physical capability predicts mortality in late mid-life as well as in old age: Findings from a large British cohort study**. *Archives of Gerontology and Geriatrics* 2018, **74**:77-82.

8. Goldberg A, Talley SA: **Performance on a test of rapid stepping in community-dwelling older adults: validity, relative and absolute reliability and minimum detectable change**. *Physiotherapy Theory & Practice* 2015, **31**(7):483-488.

9. Alfred T, Ben-Shlomo Y, Cooper R, Hardy R, Cooper C, Deary IJ, Gaunt TR, Gunnell D, Harris SE, Kumari M *et al*: **A multi-cohort study of polymorphisms in the GH/IGF axis and physical capability: the HALCyon programme**. *PLoS ONE [Electronic Resource]* 2012, **7**(1):e29883.

10. Scaglioni-Solano P, Aragon-Vargas LF: **Gait characteristics and sensory abilities of older adults are modulated by gender**. *Gait & Posture* 2015, **42**(1):54-59.

11. Won H, Singh DK, Din NC, Badrasawi M, Manaf ZA, Tan ST, Tai CC, Shahar S: **Relationship between physical performance and cognitive performance measures among community-dwelling older adults**. *Clinical Epidemiology* 2014, **6**:343-350.

12. Hsieh KL, Roach KL, Wajda DA, Sosnoff JJ: **Smartphone technology can measure postural stability and discriminate fall risk in older adults**. *Gait & Posture* 2019, **67**:160-165.

13. McBean AL, Najjar RP, Schuchard RA, Hall CD, Wang C-A, Ku B, Zeitzer JM: **Standing balance and spatiotemporal aspects of gait are impaired upon nocturnal awakening in healthy late middle-aged and older adults**. *Journal of Clinical Sleep Medicine* 2016, **12**(11):1477-1486.

14. Quek J, Treleaven J, Clark RA, Brauer SG: **An exploratory study examining factors underpinning postural instability in older adults with idiopathic neck pain**. *Gait & Posture* 2018, **60**:93-98.

15. Fleury A, Mourcou Q, Franco C, Diot B, Demongeot J, Vuillerme N: **Evaluation of a Smartphone-based audio-biofeedback system for improving balance in older adults--a pilot study**. *Conference Proceedings: Annual International Conference of the IEEE Engineering in Medicine & Biology Society* 2013, **2013**:1198-1201.

16. Nicholson VP, McKean MR, Burkett BJ: **Twelve weeks of BodyBalance training improved balance and functional task performance in middle-aged and older adults**. *Clinical Interventions In Aging* 2014, **9**:1895-1904.

17. Nicholson VP, McKean MR, Burkett BJ: **Low-load high-repetition resistance training improves strength and gait speed in middle-aged and older adults**. *Journal of Science & Medicine in Sport* 2015, **18**(5):596-600.

18. Hageman PA, Leibowitz JM, Blanke D: **Age and gender effects on postural control measures**. *Archives of Physical Medicine & Rehabilitation* 1995, **76**(10):961-965.

19. Shaw JA, Stefanyk LE, Frank JS, Jog MS, Adkin AL: **Effects of age and pathology on stance modifications in response to increased postural threat**. *Gait & Posture* 2012, **35**(4):658-661.

20. Quek JM, Pua YH, Bryant AL, Clark RA: **The influence of cervical spine flexion-rotation range-of-motion asymmetry on postural stability in older adults**. *Spine* 2013, **38**(19):1648-1655.

21. Brotherton SS, Williams HG, Gossard JL, Hussey JR, McClenaghan BA, Eleazer P: **Are measures employed in the assessment of balance useful for detecting differences among groups that vary by age and disease state?** *Journal of Geriatric Physical Therapy* 2005, **28**(1):14-19.

22. Scaglioni-Solano P, Aragon-Vargas LF: **Validity and reliability of the Nintendo Wii Balance Board to assess standing balance and sensory integration in highly functional older adults**. *International Journal of Rehabilitation Research* 2014, **37**(2):138-143.

23. Stijntjes M, de Craen AJ, van der Grond J, Meskers CG, Slagboom PE, Maier AB: **Cerebral Microbleeds and Lacunar Infarcts Are Associated with Walking Speed Independent of Cognitive Performance in Middle-Aged to Older Adults**. *Gerontology* 2016, **15**:15.

24. Medell JL, Alexander NB: **A clinical measure of maximal and rapid stepping in older women**. *Journals of Gerontology Series A-Biological Sciences & Medical Sciences* 2000, **55**(8):M429-433.

25. Abe T, Ogawa M, Loenneke JP, Thiebaud RS, Loftin M, Mitsukawa N: **Association between site-specific muscle loss of lower body and one-leg standing balance in active women: the HIREGASAKI study**. *Geriatrics & gerontology international* 2014, **14**(2):381-387.

26. Balachandran A, C NV, Potiaumpai M, Ni M, Signorile JF: **Validity and reliability of a video questionnaire to assess physical function in older adults**. *Experimental Gerontology* 2016, **16**:16.

27. Martin H, Aihie Sayer A, Jameson K, Syddall H, Dennison EM, Cooper C, Robinson S: **Does diet influence physical performance in community-dwelling older people? Findings from the Hertfordshire Cohort Study**. *Age & Ageing* 2011, **40**(2):181-186.

28. St-Arnaud-McKenzie D, Payette H, Gray-Donald K: **Low physical function predicts either 2-year weight loss or weight gain in healthy community-dwelling older adults. the NuAge Longitudinal Study**. *Journals of Gerontology Series A-Biological Sciences & Medical Sciences* 2010, **65**(12):1362-1368.

29. Vaughan S, Wallis M, Polit D, Steele M, Shum D, Morris N: **The effects of multimodal exercise on cognitive and physical functioning and brain-derived neurotrophic factor in older women: a randomised controlled trial**. *Age & Ageing* 2014, **43**(5):623-629.

30. Jalali MM, Gerami H, Heidarzadeh A, Soleimani R: **Balance performance in older adults and its relationship with falling**. *Aging-Clinical & Experimental Research* 2015, **27**(3):287-296.

31. Chen HT, Lin CH, Yu LH: **Normative physical fitness scores for community-dwelling older adults**. *Journal of Nursing Research* 2009, **17**(1):30-41.

32. Rudisill ME, Toole T: **Gender differences in motor performance of 50- to 79-year-old adults**. *Perceptual & Motor Skills* 1993, **77**(3 Pt 1):939-947.

33. Liu XY, Gao J, Yin BX, Yang XY, Bai DX: **Efficacy of Ba Duan Jin in improving balance: A study in Chinese community-dwelling older adults**. *Journal of Gerontological Nursing* 2016, **42**(5):38-46.

34. Edholm P, Strandberg E, Kadi F: **Lower limb explosive strength capacity in elderly women: effects of resistance training and healthy diet**. *Journal of Applied Physiology* 2017, **123**(1):190-196.

35. Wayne PM, Gow BJ, Costa MD, Peng CK, Lipsitz LA, Hausdorff JM, Davis RB, Walsh JN, Lough M, Novak V *et al*: **Complexity-based measures inform effects of Tai Chi training on standing postural control: Cross-sectional and randomized trial Studies**. *PLoS One* 2014, **9**(12):e114731.

36. Kokai Y, Mikami N, Tada M, Tomonobu K, Ochiai R, Osaki N, Katsuragi Y, Sohma H, Ito YM: **Effects of dietary supplementation with milk fat globule membrane on the physical performance of community-dwelling Japanese adults: a randomised, double-blind, placebo-controlled trial**. *Journal of Nutritional Science* 2018, **7**.

37. Schilling BK, Falvo MJ, Karlage RE, Weiss LW, Lohnes CA, Chiu LZ: **Effects of unstable surface training on measures of balance in older adults**. *Journal of Strength & Conditioning Research* 2009, **23**(4):1211-1216.

38. Kuo YL, Huang KY, Chiang PT, Lee PY, Tsai YJ: **Steadiness of spinal regions during single-leg standing in older adults with and without chronic low back pain**. *PLoS One* 2015, **10**(5):e0128318.

39. Fujisawa T, Suzuki S, Tanaka K, Kamekura N, Fukushima K, Kemmotsu O: **Recovery of postural stability following conscious sedation with midazolam in the elderly**. *Journal of Anesthesia* 2002, **16**(3):198-202.

40. Hardy R, Cooper R, Aihie Sayer A, Ben-Shlomo Y, Cooper C, Deary IJ, Demakakos P, Gallacher J, Martin RM, McNeill G *et al*: **Body mass index, muscle strength and physical performance in older adults from eight cohort studies: the HALCyon programme**. *PLoS ONE [Electronic Resource]* 2013, **8**(2):e56483.

41. Demnitz N, Zsoldos E, Mahmood A, Mackay CE, Kivimäki M, Singh-Manoux A, Dawes H, Johansen-Berg H, Ebmeier KP, Sexton CE: **Associations between mobility, cognition, and brain structure in healthy older adults**. *Frontiers in Aging Neuroscience* 2017, **9**:155.

42. Chen TC-C, Tseng W-C, Huang G-L, Chen H-L, Tseng K-W, Nosaka K: **Superior effects of eccentric to concentric knee extensor resistance training on physical fitness, insulin sensitivity and lipid profiles of elderly men**. *Frontiers in Physiology* 2017, **8**:209.

43. Paes T, Belo LF, da Silva DR, Morita AA, Donária L, Furlanetto KC, Sant'Anna T, Pitta F, Hernandes NA: **Londrina activities of daily living protocol: reproducibility, validity, and reference values in physically independent adults Age 50 years and older**. *Respiratory Care* 2017:respcare. 05059.

44. Porto JM, Júnior RCF, Bocarde L, Fernandes JA, Marques NR, Rodrigues NC, de Abreu DCC: **Contribution of hip abductor–adductor muscles on static and dynamic balance of community-dwelling older adults**. *Aging Clinical and Experimental Eesearch* 2018:1-7.

45. Gothe NP, McAuley E: **Yoga Is as good as stretching-strengthening exercises in improving functional fitness outcomes: results from a randomized controlled trial**. *Journals of Gerontology Series A-Biological Sciences & Medical Sciences* 2016, **71**(3):406-411.

46. Santoni G, Angleman SB, Ek S, Heiland EG, Lagergren M, Fratiglioni L, Welmer A-K: **Temporal trends in impairments of physical function among older adults during 2001–16 in Sweden: towards a healthier ageing**. *Age and Ageing* 2018.

47. Hulya TD, Sevi YS, Serap A, Ayse OE: **Factors affecting the benefits of a six-month supervised exercise program on community-dwelling older adults: interactions among age, gender, and participation**. *Journal of Physical Therapy Science* 2015, **27**(5):1421-1427.

48. Coelho-Junior HJ, Rodrigues B, de Oliveira Gonçalves I, Asano RY, Uchida MC, Marzetti E: **The physical capabilities underlying timed “Up and Go” test are time-dependent in community-dwelling older women**. *Experimental Gerontology* 2018, **104**:138-146.

49. Cooper AJ, Simmons RK, Kuh D, Brage S, Cooper R, scientific N, data collection t: **Physical activity, sedentary time and physical capability in early old age: British birth cohort study**. *PLoS ONE [Electronic Resource]* 2015, **10**(5):e0126465.

50. Yıldırım P, Ofluoglu D, Aydogan S, Akyuz G: **Tai chi vs. combined exercise prescription: a comparison of their effects on factors related to falls**. *Journal of Back and Musculoskeletal Rehabilitation* 2016, **29**(3):493-501.

51. Chun SH, Cho B, Yang H-K, Ahn E, Han MK, Oh B, Shin DW, Son KY: **Performance on physical function tests and the risk of fractures and admissions: findings from a national health screening of 557,648 community-dwelling older adults**. *Archives of Gerontology and Geriatrics* 2017, **68**:174-180.

52. Benavent-Caballer V, Rosado-Calatayud P, Segura-Orti E, Amer-Cuenca JJ, Lison JF: **The effectiveness of a video-supported group-based Otago exercise programme on physical performance in community-dwelling older adults: a preliminary study**. *Physiotherapy* 2015, **1**:1.

53. Malmberg JJ, Miilunpalo SI, Vuori IM, Pasanen ME, Oja P, Haapanen-Niemi NA: **A health-related fitness and functional performance test battery for middle-aged and older adults: feasibility and health-related content validity**. *Archives of Physical Medicine & Rehabilitation* 2002, **83**(5):666-677.

54. Rogers CE, Nseir S, Keller C: **Sign Chi Do and physical function: a pilot study**. *Geriatric Nursing* 2013, **34**(1):12-18.

55. Inoue W, Ikezoe T, Tsuboyama T, Sato I, Malinowska KB, Kawaguchi T, Tabara Y, Nakayama T, Matsuda F, Ichihashi N: **Are there different factors affecting walking speed and gait cycle variability between men and women in community-dwelling older adults?** *Aging Clinical and Experimental Research* 2017, **29**(2):215-221.

56. Brown M, Holloszy JO: **Effects of walking, jogging and cycling on strength, flexibility, speed and balance in 60- to 72-year olds**. *Aging-Clinical & Experimental Research* 1993, **5**(6):427-434.

57. Chung PK, Zhao Y, Liu JD, Quach B: **Functional fitness norms for community-dwelling older adults in Hong Kong**. *Archives of Gerontology & Geriatrics* 2016, **65**:54-62.

58. Mickle KJ, Caputi P, Potter JM, Steele JR: **Efficacy of a progressive resistance exercise program to increase toe flexor strength in older people**. *Clinical Biomechanics* 2016, **40**:14-19.

59. Nakamura M, Hashizume H, Oka H, Okada M, Takakura R, Hisari A, Yoshida M, Utsunomiya H: **Physical performance measures associated with locomotive syndrome in middle-aged and older Japanese women**. *Journal of Geriatric Physical Therapy* 2015, **38**(4):202-207.

60. Demnitz N, Hogan DB, Dawes H, Johansen-Berg H, Ebmeier KP, Poulin MJ, Sexton CE: **Cognition and mobility show a global association in middle-and late-adulthood: Analyses from the Canadian longitudinal study on aging**. *Gait & Posture* 2018.

61. Urushihata T, Kinugasa T, Soma Y, Miyoshi H: **Aging effects on the structure underlying balance abilities tests**. *Journal of the Japanese Physical Therapy Association* 2010, **13**(1):1-8.

62. Heiland EG, Welmer AK, Wang R, Santoni G, Angleman S, Fratiglioni L, Qiu C: **Association of mobility limitations with incident disability among older adults: a population-based study**. *Age & Ageing* 2016, **28**:28.

63. Welmer AK, Kareholt I, Angleman S, Rydwik E, Fratiglioni L: **Can chronic multimorbidity explain the age-related differences in strength, speed and balance in older adults?** *Aging-Clinical & Experimental Research* 2012, **24**(5):480-489.

64. Heiland EG, Welmer A-K, Wang R, Santoni G, Angleman S, Fratiglioni L, Qiu C: **Association of mobility limitations with incident disability among older adults: a population-based study**. *Age and Ageing* 2016, **45**(6):812-819.

65. Hafström A: **Perceived and functional balance control is negatively affected by diminished touch and vibration sensitivity in relatively healthy older adults and elderly**. *Gerontology and Geriatric Medicine* 2018, **4**:2333721418775551.

66. Sasai H, Matsuo T, Numao S, Sakai T, Mochizuki M, Kuroda K, Okamoto M, Tanaka K: **Aotake: a modified stepping exercise as a useful means of improving lower-extremity functional fitness in older adults**. *Geriatrics & gerontology international* 2010, **10**(3):244-250.

67. Shigematsu R, Okura T, Sakai T, Rantanen T: **Square-stepping exercise versus strength and balance training for fall risk factors**. *Aging-Clinical & Experimental Research* 2008, **20**(1):19-24.

68. Hong Y, Li JX, Robinson PD: **Balance control, flexibility, and cardiorespiratory fitness among older Tai Chi practitioners**. *British Journal of Sports Medicine* 2000, **34**(1):29-34.

69. Moriya S, Notani K, Murata A, Inoue N, Miura H: **Analysis of moment structures for assessing relationships among perceived chewing ability, dentition status, muscle strength, and balance in community-dwelling older adults**. *Gerodontology* 2014, **31**(4):281-287.

70. Gschwind YJ, Kressig RW, Lacroix A, Muehlbauer T, Pfenninger B, Granacher U: **A best practice fall prevention exercise program to improve balance, strength / power, and psychosocial health in older adults: study protocol for a randomized controlled trial**. *BMC Geriatrics* 2013, **13**:105.

71. Daly RM, Ahlborg HG, Ringsberg K, Gardsell P, Sernbo I, Karlsson MK: **Association between changes in habitual physical activity and changes in bone density, muscle strength, and functional performance in elderly men and women**. *Journal of the American Geriatrics Society* 2008, **56**(12):2252-2260.

72. Xie YJ, Liu EY, Anson ER, Agrawal Y: **Age-related imbalance is associated with slower walking speed: an analysis from the National Health and nutrition examination survey**. *Journal of Geriatric Physical Therapy* 2017, **40**(4):183-189.

73. Bloem BR, Valkenburg VV, Slabbekoorn M, van Dijk JG: **The multiple tasks test. Strategies in Parkinson's disease**. *Experimental Brain Research* 2001, **137**(3-4):478-486.

74. Bulbulian R, Hargan ML: **The effect of activity history and current activity on static and dynamic postural balance in older adults**. *Physiology & Behavior* 2000, **70**(3-4):319-325.

75. Misic MM, Rosengren KS, Woods JA, Evans EM: **Muscle quality, aerobic fitness and fat mass predict lower-extremity physical function in community-dwelling older adults**. *Gerontology* 2007, **53**(5):260-266.

76. Gao KL, Hui-Chan CW, Tsang WW: **Golfers have better balance control and confidence than healthy controls**. *European Journal of Applied Physiology* 2011, **111**(11):2805-2812.

77. Lee S-C: **Relationship of visual dependence to age, balance, attention, and vertigo**. *Journal of Physical Therapy Science* 2017, **29**(8):1318-1322.

78. Gordt K, Mikolaizak AS, Nerz C, Barz C, Gerhardy T, Weber M, Becker C, Schwenk M: **German version of the Community Balance and Mobility Scale**. *Zeitschrift für Gerontologie und Geriatrie* 2018:1-9.

79. Weber M, Van Ancum J, Bergquist R, Taraldsen K, Gordt K, Mikolaizak AS, Nerz C, Pijnappels M, Jonkman NH, Maier AB: **Concurrent validity and reliability of the Community Balance and Mobility scale in young-older adults**. *BMC Geriatrics* 2018, **18**(1):156.

80. Frey DJ, Ortega JD, Wiseman C, Farley CT, Wright KP, Jr.: **Influence of zolpidem and sleep inertia on balance and cognition during nighttime awakening: a randomized placebo-controlled trial**. *Journal of the American Geriatrics Society* 2011, **59**(1):73-81.

81. Goudarzian M, Ghavi S, Shariat A, Shirvani H, Rahimi M: **Effects of whole body vibration training and mental training on mobility, neuromuscular performance, and muscle strength in older men**. *Journal of Exercise Rehabilitation* 2017, **13**(5):573.

82. Tarnopolsky M, Zimmer A, Paikin J, Safdar A, Aboud A, Pearce E, Roy B, Doherty T: **Creatine monohydrate and conjugated linoleic acid improve strength and body composition following resistance exercise in older adults**. *PLoS ONE [Electronic Resource]* 2007, **2**(10):e991.

83. da Silva Costa AA, dos Santos LO, Mauerberg-deCastro E, Moraes R: **Task difficulty has no effect on haptic anchoring during tandem walking in young and older adults**. *Neuroscience Letters* 2018, **666**:133-138.

84. Bird M, Hill KD, Ball M, Hetherington S, Williams AD: **The long-term benefits of a multi-component exercise intervention to balance and mobility in healthy older adults**. *Archives of Gerontology & Geriatrics* 2011, **52**(2):211-216.

85. Vrantsidis F, Hill K, Haralambous B, Renehan E, Ledgerwood K, Pinikahana J, Harper S, Penberthy M: **Living Longer Living StrongerTM: a community-delivered strength training program improving function and quality of life**. *Australasian Journal on Ageing* 2014, **33**(1):22-25.

86. Bird ML, Hill KD, Fell JW: **A randomized controlled study investigating static and dynamic balance in older adults after training with Pilates**. *Archives of Physical Medicine & Rehabilitation* 2012, **93**(1):43-49.

87. Gianoudis J, Bailey CA, Ebeling PR, Nowson CA, Sanders KM, Hill K, Daly RM: **Effects of a targeted multimodal exercise program incorporating high-speed power training on falls and fracture risk factors in older adults: a community-based randomized controlled trial**. *Journal of Bone & Mineral Research* 2014, **29**(1):182-191.

88. Justine M, Ruzali D, Hazidin E, Said A, Bukry SA, Manaf H: **Range of motion, muscle length, and balance performance in older adults with normal, pronated, and supinated feet**. *Journal of Physical Therapy Science* 2016, **28**(3):916-922.

89. Pirotta S, Kidgell DJ, Daly RM: **Effects of vitamin D supplementation on neuroplasticity in older adults: a double-blinded, placebo-controlled randomised trial**. *Osteoporosis International* 2015, **26**(1):131-140.

90. Robinson RL, Ng SS: **The timed 180° turn test for assessing people with hemiplegia from chronic stroke**. *BioMed Research International* 2018, **2018**.

91. Tsai Y-J, Yang Y-C, Lu F-H, Lee P-Y, Lee I-T, Lin S-I: **Functional balance and its determinants in older people with diabetes**. *PloS One* 2016, **11**(7):e0159339.

92. Galvao DA, Taaffe DR: **Resistance exercise dosage in older adults: single- versus multiset effects on physical performance and body composition**. *Journal of the American Geriatrics Society* 2005, **53**(12):2090-2097.

93. Henwood TR, Taaffe DR: **Short-term resistance training and the older adult: the effect of varied programmes for the enhancement of muscle strength and functional performance**. *Clinical Physiology & Functional Imaging* 2006, **26**(5):305-313.

94. Henwood TR, Taaffe DR: **Detraining and retraining in older adults following long-term muscle power or muscle strength specific training**. *Journals of Gerontology Series A-Biological Sciences & Medical Sciences* 2008, **63**(7):751-758.

95. Ng SS, Fong SS, Chan CW, Fung FM, Pang PK, Tsang NN, Yeung CL, Kwong PW, Tse MM, Tam EW: **Floor transfer test for assessing people with chronic stroke**. *Journal of Rehabilitation Medicine* 2015, **47**(6):489-494.

96. Ward CL, Valentine RJ, Evans EM: **Greater effect of adiposity than physical activity or lean mass on physical function in community-dwelling older adults**. *Journal of Aging & Physical Activity* 2014, **22**(2):284-293.

97. Zhuang J, Huang L, Wu Y, Zhang Y: **The effectiveness of a combined exercise intervention on physical fitness factors related to falls in community-dwelling older adults**. *Clinical Interventions In Aging* 2014, **9**:131-140.

98. Teixeira CV, Gobbi S, Pereira JR, Ueno DT, Shigematsu R, Gobbi LT: **Effect of square-stepping exercise and basic exercises on functional fitness of older adults**. *Geriatrics & gerontology international* 2013, **13**(4):842-848.

99. Wood RH, Reyes R, Welsch MA, Favaloro-Sabatier J, Sabatier M, Matthew Lee C, Johnson LG, Hooper PF: **Concurrent cardiovascular and resistance training in healthy older adults**. *Medicine & Science in Sports & Exercise* 2001, **33**(10):1751-1758.

100. Kwok BC, Pua YH: **Effects of WiiActive exercises on fear of falling and functional outcomes in community-dwelling older adults: a randomised control trial**. *Age and Ageing* 2016, **45**(5):621-627.

101. Ng SS, Lau BK, Law GT, Wom CW, Liu T-W, Tam EW, Tse MM, Fong SS: **Sideways walk test: reliability and association with lower limb motor function after stroke**. *Journal of Rehabilitation Medicine* 2016, **48**(8):657-665.

102. Pozzi F, White DK, Snyder-Mackler L, Zeni JA: **Restoring physical function after knee replacement: a cross sectional comparison of progressive strengthening vs standard physical therapy**. *Physiotherapy Theory and Practice* 2018:1-12.

103. Fife E, Kostka J, Kroc Ł, Guligowska A, Pigłowska M, Sołtysik B, Kaufman-Szymczyk A, Fabianowska-Majewska K, Kostka T: **Relationship of muscle function to circulating myostatin, follistatin and GDF11 in older women and men**. *BMC Geriatrics* 2018, **18**(1):200.

104. Coetsee C, Terblanche E: **The effect of three different exercise training modalities on cognitive and physical function in a healthy older population**. *European Review of Aging and Physical Activity* 2017, **14**(1):13.

105. Gusi N, Hernandez-Mocholi MA, Olivares PR: **Changes in HRQoL after 12 months of exercise linked to primary care are associated with fitness effects in older adults**. *European Journal of Public Health* 2015, **25**(5):873-879.

106. Weiss A, Herman T, Plotnik M, Brozgol M, Maidan I, Giladi N, Gurevich T, Hausdorff JM: **Can an accelerometer enhance the utility of the Timed Up & Go Test when evaluating patients with Parkinson's disease?** *Medical Engineering & Physics* 2010, **32**(2):119-125.

107. Uritani D, Fukumoto T, Matsumoto D, Shima M: **The relationship between toe grip strength and dynamic balance or functional mobility among community-dwelling Japanese older adults: a cross-sectional study**. *Journal of Aging and Physical Activity* 2016, **24**(3):459-464.

108. Hoang OTT, Jullamate P, Piphatvanitcha N, Rosenberg E: **Factors related to fear of falling among community‐dwelling older adults**. *Journal of Clinical Nursing* 2017, **26**(1-2):68-76.

109. Duclos C, Mieville C, Gagnon D, Leclerc C: **Dynamic stability requirements during gait and standing exergames on the wii fit system in the elderly**. *Journal of Neuroengineering & Rehabilitation* 2012, **9**:28.

110. Alonso AC, Ribeiro SM, Luna NMS, Peterson MD, Bocalini DS, Serra MM, Brech GC, Greve JMDA, Garcez-Leme LE: **Association between handgrip strength, balance, and knee flexion/extension strength in older adults**. *PloS One* 2018, **13**(6):e0198185.

111. Olivares PR, Gusi N, Prieto J, Hernandez-Mocholi MA: **Fitness and health-related quality of life dimensions in community-dwelling middle aged and older adults**. *Health & Quality of Life Outcomes* 2011, **9**:117.

112. Ng SS, Fong SS, Chan WL, Hung BK, Chung RK, Chim TH, Kwong PW, Liu T-W, Tse MM, Chung RC: **The sitting and rising test for assessing people with chronic stroke**. *Journal of Physical Therapy Science* 2016, **28**(6):1701-1708.

113. Campos de Oliveira L, Goncalves de Oliveira R, Pires-Oliveira DA: **Effects of Pilates on muscle strength, postural balance and quality of life of older adults: a randomized, controlled, clinical trial**. *Journal of Physical Therapy Science* 2015, **27**(3):871-876.

114. Beauchet O, Launay CP, Sejdic E, Allali G, Annweiler C: **Motor imagery of gait: a new way to detect mild cognitive impairment?** *Journal of Neuroengineering & Rehabilitation* 2014, **11**:66.

115. Coetsee C, Terblanche E: **The time course of changes induced by resistance training and detraining on muscular and physical function in older adults**. *European Reviews of Aging & Physical Activity* 2015, **12**:7.

116. Simpson LA, Miller WC, Eng JJ: **Effect of stroke on fall rate, location and predictors: a prospective comparison of older adults with and without stroke**. *PLoS ONE [Electronic Resource]* 2011, **6**(4):e19431.

117. Yu W, An C, Kang H: **Effects of resistance exercise using thera-band on balance of elderly adults: A randomized controlled trial**. *Journal of Physical Therapy Science* 2013, **25**(11):1471-1473.

118. Van Roie E, Delecluse C, Coudyzer W, Boonen S, Bautmans I: **Strength training at high versus low external resistance in older adults: effects on muscle volume, muscle strength, and force-velocity characteristics**. *Experimental Gerontology* 2013, **48**(11):1351-1361.

119. Lamarche L, Gammage KL, Klentrou P, Adkin AL: **What will they think? The relationship between self-presentational concerns and balance and mobility outcomes in older women**. *Experimental Aging Research* 2014, **40**(4):426-435.

120. Wang X, Ma Y, Wang J, Han P, Dong R, Kang L, Zhang W, Shen S, Wang J, Li D *et al*: **Mobility and muscle strength together are more strongly correlated with falls in suburb-dwelling older Chinese**. *Scientific Reports* 2016, **6**:25420.

121. Wu IC, Chang HY, Hsu CC, Chiu YF, Yu SH, Tsai YF, Shen SC, Kuo KN, Chen CY, Liu K *et al*: **Association between dietary fiber intake and physical performance in older adults: a nationwide study in Taiwan**. *PLoS ONE [Electronic Resource]* 2013, **8**(11):e80209.

122. Tsuji T, Kitano N, Tsunoda K, Himori E, Okura T, Tanaka K: **Short-term effects of whole-body vibration on functional mobility and flexibility in healthy, older adults: a randomized crossover study**. *Journal of Geriatric Physical Therapy* 2014, **37**(2):58-64.

123. Uemura SI, Kanbayashi T, Wakasa M, Satake M, Ito W, Shimizu K, Shioya T, Shimizu T, Nishino S: **Residual effects of zolpidem, triazolam, rilmazafone and placebo in healthy elderly subjects: a randomized double-blind study**. *Sleep Medicine* 2015, **16**(11):1395-1402.

124. Siervo M, Oggioni C, Jakovljevic DG, Trenell M, Mathers JC, Houghton D, Celis-Morales C, Ashor AW, Ruddock A, Ranchordas M: **Dietary nitrate does not affect physical activity or outcomes in healthy older adults in a randomized, cross-over trial**. *Nutrition Research* 2016, **36**(12):1361-1369.

125. Furness TP, Maschette WE, Lorenzen C, Naughton GA, Williams MD: **Efficacy of a whole-body vibration intervention on functional performance of community-dwelling older adults**. *Journal of Alternative & Complementary Medicine* 2010, **16**(7):795-797.

126. Houck J, Kneiss J, Bukata SV, Puzas JE: **Analysis of vertical ground reaction force variables during a Sit to Stand task in participants recovering from a hip fracture**. *Clinical Biomechanics* 2011, **26**(5):470-476.

127. Gault ML, Clements RE, Willems ME: **Functional mobility of older adults after concentric and eccentric endurance exercise**. *European Journal of Applied Physiology* 2012, **112**(11):3699-3707.

128. Meng Y, Wu H, Yang Y, Du H, Xia Y, Guo X, Liu X, Li C, Niu K: **Relationship of anabolic and catabolic biomarkers with muscle strength and physical performance in older adults: a population-based cross-sectional study**. *BMC Musculoskeletal Disorders* 2015, **16**:202.

129. Kwok BC, Clark RA, Pua YH: **Novel use of the Wii Balance Board to prospectively predict falls in community-dwelling older adults**. *Clinical Biomechanics* 2015, **30**(5):481-484.

130. Nagamatsu LS, Weinstein AM, Erickson KI, Fanning J, Awick EA, Kramer AF, McAuley E: **Exercise mode moderates the relationship between mobility and basal ganglia volume in healthy older adults**. *Journal of the American Geriatrics Society* 2016, **64**(1):102-108.

131. Rogers HL, Cromwell RL, Grady JL: **Adaptive changes in gait of older and younger adults as responses to challenges to dynamic balance**. *Journal of Aging & Physical Activity* 2008, **16**(1):85-96.

132. Mulford D, Taggart HM, Nivens A, Payrie C: **Arch support use for improving balance and reducing pain in older adults**. *Applied Nursing Research* 2008, **21**(3):153-158.

133. Canney M, Sexton DJ, O’connell MD, Kenny RA, Little MA, O’seaghdha CM: **Kidney function estimated from cystatin C, but not creatinine, is related to objective tests of physical performance in community-dwelling older adults**. *Journals of Gerontology Series A: Biomedical Sciences and Medical Sciences* 2017, **72**(11):1554-1560.

134. Araujo TB, Silva NA, Costa JN, Pereira MM, Safons MP: **Effect of equine-assisted therapy on the postural balance of the elderly**. *Revista Brasileira de Fisioterapia* 2011, **15**(5):414-419.

135. Valentine RJ, Misic MM, Rosengren KS, Woods JA, Evans EM: **Sex impacts the relation between body composition and physical function in older adults**. *Menopause* 2009, **16**(3):518-523.

136. Dias CP, Toscan R, de Camargo M, Pereira EP, Griebler N, Baroni BM, Tiggemann CL: **Effects of eccentric-focused and conventional resistance training on strength and functional capacity of older adults**. *Age* 2015, **37**(5):99.

137. Morat T, Mechling H: **The functional movement circle for older adults: feasibility and effects on physical performance**. *Aging-Clinical & Experimental Research* 2014, **26**(5):529-537.

138. Vervoort D, Vuillerme N, Kosse N, Hortobágyi T, Lamoth CJ: **Multivariate analyses and classification of inertial sensor data to identify aging effects on the Timed-Up-and-Go test**. *PloS One* 2016, **11**(6):e0155984.

139. Merchant RA, Banerji S, Singh G, Chew E, Poh CL, Tapawan SC, Guo YR, Pang YW, Sharma M, Kambadur R *et al*: **Is trunk posture in walking a better marker than gait speed in predicting decline in function and subsequent frailty?** *Journal of the American Medical Directors Association* 2016, **17**(1):65-70.

140. Voos MC, Custodio EB, Malaquias J, Jr.: **Relationship of executive function and educational status with functional balance in older adults**. *Journal of Geriatric Physical Therapy* 2011, **34**(1):11-18.

141. Buskard A, Zalma B, Cherup N, Armitage C, Dent C, Signorile JF: **Effects of linear periodization versus daily undulating periodization on neuromuscular performance and activities of daily living in an elderly population**. *Experimental Gerontology* 2018, **113**:199-208.

142. Foong YC, Chherawala N, Aitken D, Scott D, Winzenberg T, Jones G: **Accelerometer‐determined physical activity, muscle mass, and leg strength in community‐dwelling older adults**. *Journal of Cachexia, Sarcopenia and Muscle* 2016, **7**(3):275-283.

143. Raj IS, Bird SR, Westfold BA, Shield AJ: **Determining criteria to predict repeatability of performance in older adults: Using coefficients of variation for strength and functional measures**. *Journal of Aging and Physical Activity* 2017, **25**(1):94-98.

144. Balachandran AT, Gandia K, Jacobs KA, Streiner DL, Eltoukhy M, Signorile JF: **Power training using pneumatic machines vs. plate-loaded machines to improve muscle power in older adults**. *Experimental Gerontology* 2017, **98**:134-142.

145. Van Roie E, Walker S, Van Driessche S, Baggen R, Coudyzer W, Bautmans I, Delecluse C: **Training load does not affect detraining's effect on muscle volume, muscle strength and functional capacity among older adults**. *Experimental Gerontology* 2017, **98**:30-37.

146. Bellumori M, Uygur M, Knight CA: **High-speed cycling intervention improves rate-dependent mobility in older adults**. *Medicine and Science in Sports and Exercise* 2017, **49**(1):106.

147. Eckardt N: **Lower-extremity resistance training on unstable surfaces improves proxies of muscle strength, power and balance in healthy older adults: a randomised control trial**. *BMC Geriatrics* 2016, **16**(1):191.

148. Noopud P, Suputtitada A, Khongprasert S, Kanungsukkasem V: **Effects of thai traditional dance on balance performance in daily life among older women**. *Aging Clinical and Experimental Research* 2018:1-7.

149. Ng SS, Tse MM, Tam EW, Lai CY: **The psychometric properties of the toe tap test in people with stroke**. *Disability and Rehabilitation* 2018:1-9.

150. Merchant RA, van Dam R, Tan L, Lim M, Low J, Morley J: **Vitamin D binding protein and vitamin D levels in multi-ethnic population**. *The Journal of Nutrition, Health & Aging* 2018:1-6.

151. Afridi A, Malik AN, Ali S, Amjad I: **Effect of balance training in older adults using Wii fit plus**. *JPMA The Journal of the Pakistan Medical Association* 2018, **68**(3):480-483.

152. Lou L, Zou L, Fang Q, Wang H, Liu Y, Tian Z, Han Y: **Effect of Taichi softball on function-related outcomes in older adults: A randomized control trial**. *Evidence-Based Complementary and Alternative Medicine* 2017, **2017**.

153. Shahtahmassebi B, Hebert JJ, Hecimovich MD, Fairchild TJ: **Associations between trunk muscle morphology, strength and function in older adults**. *Scientific Reports* 2017, **7**(1):10907.

154. Bates A, Donaldson A, Lloyd B, Castell S, Krolik P, Coleman R: **Staying active, staying strong: pilot evaluation of a once-weekly, community-based strength training program for older adults**. *Health Promotion Journal of Australia* 2009, **20**(1):42-47.

155. Lee M, Son J, Kim J, Yoon B: **Individualized feedback-based virtual reality exercise improves older women's self-perceived health: a randomized controlled trial**. *Archives of Gerontology & Geriatrics* 2015, **61**(2):154-160.

156. Reichert T, Kanitz AC, Delevatti RS, Bagatini NC, Barroso BM, Kruel LF: **Continuous and interval training programs using deep water running improves functional fitness and blood pressure in the older adults**. *Age* 2016, **38**(1):20.

157. Sousa N, Mendes R, Abrantes C, Sampaio J, Oliveira J: **Effectiveness of combined exercise training to improve functional fitness in older adults: A randomized controlled trial**. *Geriatrics & gerontology international* 2014, **14**(4):892-898.

158. Zettel-Watson L, Suen M, Wehbe L, Rutledge DN, Cherry BJ: **Aging well: Processing speed inhibition and working memory related to balance and aerobic endurance**. *Geriatrics & gerontology international* 2015, **23**:23.

159. Zhao Q, Wang J, Feng W, Jia W, Burke LE, Zgibor JC, Sun M: **Assessing physical performance in free-living older adults with a wearable computer**. *Proceedings of the IEEE Annual Northeast Bioengineering Conference* 2015.

160. Mason RC, Horvat M, Nocera J: **The effects of exercise on the physical fitness of high and moderate-low functioning older adult women**. *Journal of Aging Research* 2016, **2016**.

161. Ramalho F, Santos-Rocha R, Branco M, Moniz-Pereira V, André H-I, Veloso AP, Carnide F: **Effect of 6-month community-based exercise interventions on gait and functional fitness of an older population: a quasi-experimental study**. *Clinical Interventions in Aging* 2018, **13**:595.

162. Lee M, Lim T, Lee J, Kim K, Yoon B: **Optimal retraining time for regaining functional fitness using multicomponent training after long-term detraining in older adults**. *Archives of Gerontology and Geriatrics* 2017, **73**:227-233.

163. Noradechanunt C, Worsley A, Groeller H: **Thai Yoga improves physical function and well-being in older adults: A randomised controlled trial**. *Journal of Science and Medicine in Sport* 2017, **20**(5):494-501.

164. Zettel‐Watson L, Suen M, Wehbe L, Rutledge DN, Cherry BJ: **Aging well: Processing speed inhibition and working memory related to balance and aerobic endurance**. *Geriatrics & Gerontology International* 2017, **17**(1):108-115.

165. Richardson DL, Duncan MJ, Jimenez A, Juris PM, Clarke ND: **Effects of movement velocity and training frequency of resistance exercise on functional performance in older adults: a randomised controlled trial**. *European Journal of Sport Science* 2018:1-13.

166. Prusik K, Kortas J, Prusik K, Mieszkowski J, Jaworska J, Skrobot W, Lipinski M, Ziemann E, Antosiewicz J: **Nordic walking training causes a decrease in blood cholesterol in elderly women supplemented with vitamin D**. *Frontiers in Endocrinology* 2018, **9**:42.

167. Bergamin M, Gobbo S, Bullo V, Zanotto T, Vendramin B, Duregon F, Cugusi L, Camozzi V, Zaccaria M, Neunhaeuserer D *et al*: **Effects of a Pilates exercise program on muscle strength, postural control and body composition: results from a pilot study in a group of post-menopausal women**. *Age* 2015, **37**(6):118.

168. Canuto Wanderley FA, Oliveira NL, Marques E, Moreira P, Oliveira J, Carvalho J: **Aerobic versus resistance training effects on health-related quality of life, body composition, and function of older adults**. *Journal of Applied Gerontology* 2015, **34**(3):NP143-165.

169. Carvalho MJ, Marques E, Mota J: **Training and detraining effects on functional fitness after a multicomponent training in older women**. *Gerontology* 2009, **55**(1):41-48.

170. Krause MP, Januario RS, Hallage T, Haile L, Miculis CP, Gama MP, Goss FL, da Silva SG: **A comparison of functional fitness of older Brazilian and American women**. *Journal of Aging & Physical Activity* 2009, **17**(4):387-397.

171. Toraman F, Sahin G: **Age responses to multicomponent training programme in older adults**. *Disability & Rehabilitation* 2004, **26**(8):448-454.

172. Marques EA, Mota J, Viana JL, Tuna D, Figueiredo P, Guimaraes JT, Carvalho J: **Response of bone mineral density, inflammatory cytokines, and biochemical bone markers to a 32-week combined loading exercise programme in older men and women**. *Archives of Gerontology & Geriatrics* 2013, **57**(2):226-233.

173. Milanovic Z, Pantelic S, Trajkovic N, Sporis G, Kostic R, James N: **Age-related decrease in physical activity and functional fitness among elderly men and women.[Erratum appears in Clin Interv Aging. clin interv aging. 2014;9:979]**. *Clinical Interventions In Aging* 2013, **8**:549-556.

174. Yamauchi T, Islam MM, Koizumi D, Rogers ME, Rogers NL, Takeshima N: **Effect of home-based well-rounded exercise in community-dwelling older adults**. *Journal of Sports Science & Medicine* 2005, **4**(4):563-571.

175. Palmer RC, Batra A, Anderson C, Page T, Vieira E, Seff L: **Implementation of an evidence-based exercise program for older adults in South Florida**. *Journal of Aging Research* 2016, **2016**.

176. Timmons JF, Minnock D, Hone M, Cogan KE, Murphy JC, Egan B: **Comparison of time‐matched aerobic, resistance, or concurrent exercise training in older adults**. *Scandinavian Journal of Medicine & Science in Sports* 2018, **28**(11):2272-2283.

177. Yagci N, Cavlak U, Aslan UB, Akdag B: **Relationship between balance performance and musculoskeletal pain in lower body comparison healthy middle aged and older adults**. *Archives of Gerontology & Geriatrics* 2007, **45**(1):109-119.

178. Lin SI, Tsai TT: **Muscle weakness and imbalance in older dizzy patients**. *Aging-Clinical & Experimental Research* 2005, **17**(3):168-173.

179. Nakamura Y, Tanaka K, Yabushita N, Sakai T, Shigematsu R: **Effects of exercise frequency on functional fitness in older adult women**. *Archives of Gerontology & Geriatrics* 2007, **44**(2):163-173.

180. Singh DK, Pillai SG, Tan ST, Tai CC, Shahar S: **Association between physiological falls risk and physical performance tests among community-dwelling older adults**. *Clinical Interventions In Aging* 2015, **10**:1319-1326.

181. !!! INVALID CITATION !!! [13, 70].

182. Abe T, Patterson KM, Stover CD, Geddam DA, Tribby AC, Lajza DG, Young KC: **Site-specific thigh muscle loss as an independent phenomenon for age-related muscle loss in middle-aged and older men and women**. *Age* 2014, **36**(3):9634.

183. Salkovic D, Hobert MA, Bellut C, Funer F, Renno S, Haertner L, Hasmann SE, Staebler J, Geritz J, Suenkel U: **Evidence for a selectively regulated prioritization shift depending on walking situations in older adults**. *Frontiers in Aging Neuroscience* 2017, **9**:75.

184. Schrager MA, Hilton J, Gould R, Kelly VE: **Effects of blueberry supplementation on measures of functional mobility in older adults**. *Applied Physiology, Nutrition, & Metabolism = Physiologie Appliquee, Nutrition et Metabolisme* 2015, **40**(6):543-549.

185. Elbar O, Tzedek I, Vered E, Shvarth G, Friger M, Melzer I: **A water-based training program that includes perturbation exercises improves speed of voluntary stepping in older adults: a randomized controlled cross-over trial**. *Archives of Gerontology & Geriatrics* 2013, **56**(1):134-140.

186. Kim J, Son J, Ko N, Yoon B: **Unsupervised virtual reality-based exercise program improves hip muscle strength and balance control in older adults: a pilot study**. *Archives of Physical Medicine & Rehabilitation* 2013, **94**(5):937-943.

187. Garland SJ, Stevenson TJ, Ivanova T: **Postural responses to unilateral arm perturbation in young, elderly, and hemiplegic subjects**. *Archives of Physical Medicine & Rehabilitation* 1997, **78**(10):1072-1077.

188. Lip RW, Fong SS, Ng SS, Liu KP, Guo X: **Effects of Ving Tsun Chinese martial art training on musculoskeletal health, balance performance, and self-efficacy in community-dwelling older adults**. *Journal of Physical Therapy Science* 2015, **27**(3):667-672.

189. Misic MM, Valentine RJ, Rosengren KS, Woods JA, Evans EM: **Impact of training modality on strength and physical function in older adults**. *Gerontology* 2009, **55**(4):411-416.

190. Nematollahi A, Kamali F, Ghanbari A, Etminan Z, Sobhani S: **Improving balance in older people: A double-blind randomized clinical trial of three modes of balance training**. *Journal of Aging & Physical Activity* 2015, **23**:23.

191. Stanley J, Hollands M: **A novel video-based paradigm to study the mechanisms underlying age- and falls risk-related differences in gaze behaviour during walking**. *Ophthalmic & Physiological Optics* 2014, **34**(4):459-469.

192. Stellefson M, Yannessa JF, Martel GF: **Using canonical commonality analysis to examine the predictive quality of aging and falls efficacy on balance functioning in older adults**. *Evaluation & the Health Professions* 2012, **35**(2):239-255.

193. Li Z, Wang X-X, Liang Y-Y, Chen S-Y, Sheng J, Ma S-J: **Effects of the visual-feedback-based force platform training with functional electric stimulation on the balance and prevention of falls in older adults: a randomized controlled trial**. *PeerJ* 2018, **6**:e4244.

194. Barone Gibbs B, Brach JS, Byard T, Creasy S, Davis KK, McCoy S, Peluso A, Rogers RJ, Rupp K, Jakicic JM: **Reducing sedentary behavior versus increasing moderate-to-vigorous intensity physical activity in older adults: A 12-week randomized, clinical trial**. *Journal of Aging & Health* 2016, **3**:3.

195. Beavers KM, Gordon MM, Easter L, Beavers DP, Hairston KG, Nicklas BJ, Vitolins MZ: **Effect of protein source during weight loss on body composition, cardiometabolic risk and physical performance in abdominally obese, older adults: a pilot feeding study**. *Journal of Nutrition, Health & Aging* 2015, **19**(1):87-95.

196. da Camara SM, Alvarado BE, Guralnik JM, Guerra RO, Maciel AC: **Using the Short Physical Performance Battery to screen for frailty in young-old adults with distinct socioeconomic conditions**. *Geriatrics & gerontology international* 2013, **13**(2):421-428.

197. Gray SL, Aragaki AK, LaMonte MJ, Cochrane BB, Kooperberg C, Robinson JG, Woods NF, LaCroix AZ: **Statins, angiotensin-converting enzyme inhibitors, and physical performance in older women**. *Journal of the American Geriatrics Society* 2012, **60**(12):2206-2214.

198. Hannam K, Deere K, Worrall S, Hartley A, Tobias JH: **Characterization of vertical accelerations experienced by older people attending an aerobics class designed to produce high impacts**. *Journal of Aging & Physical Activity* 2016, **24**(2):268-274.

199. Hau C, Reid KF, Wong KF, Chin RJ, Botto TJ, Eliasziw M, Bermudez OI, Fielding RA: **Collaborative evaluation of the healthy habits program: an effective community intervention to improve mobility and cognition of chinese older adults living in the U.S**. *Journal of Nutrition, Health & Aging* 2016, **20**(4):391-397.

200. Kortebein P, Symons TB, Ferrando A, Paddon-Jones D, Ronsen O, Protas E, Conger S, Lombeida J, Wolfe R, Evans WJ: **Functional impact of 10 days of bed rest in healthy older adults**. *Journals of Gerontology Series A-Biological Sciences & Medical Sciences* 2008, **63**(10):1076-1081.

201. Martins WR, Safons MP, Bottaro M, Blasczyk JC, Diniz LR, Fonseca RM, Bonini-Rocha AC, de Oliveira RJ: **Effects of short term elastic resistance training on muscle mass and strength in untrained older adults: a randomized clinical trial**. *BMC Geriatrics* 2015, **15**:99.

202. Moore-Harrison TL, Speer EM, Johnson FT, Cress ME: **The effects of aerobic training and nutrition education on functional performance in low socioeconomic older adults**. *Journal of Geriatric Physical Therapy* 2008, **31**(1):18-23.

203. Nicklas BJ, Chmelo E, Delbono O, Carr JJ, Lyles MF, Marsh AP: **Effects of resistance training with and without caloric restriction on physical function and mobility in overweight and obese older adults: a randomized controlled trial**. *American Journal of Clinical Nutrition* 2015, **101**(5):991-999.

204. Reeves GR, Whellan DJ, Patel MJ, O'Connor CM, Duncan P, Eggebeen JD, Morgan TM, Hewston LA, Pastva AM, Kitzman DW: **Comparison of frequency of frailty and severely impaired physical function in patients >60 years hospitalized with acute decompensated heart failure versus chronic stable heart failure with reduced and preserved left ventricular ejection fraction**. *American Journal of Cardiology* 2016, **6**:6.

205. Roma MF, Busse AL, Betoni RA, Melo AC, Kong J, Santarem JM, Jacob Filho W: **Effects of resistance training and aerobic exercise in elderly people concerning physical fitness and ability: a prospective clinical trial**. *Einstein* 2013, **11**(2):153-157.

206. Satariano WA, Kealey M, Hubbard A, Kurtovich E, Ivey SL, Bayles CM, Hunter RH, Prohaska TR: **Mobility disability in older adults: At the intersection of people and places**. *Gerontologist* 2016, **56**(3):525-534.

207. Stehle JR, Jr., Leng X, Kitzman DW, Nicklas BJ, Kritchevsky SB, High KP: **Lipopolysaccharide-binding protein, a surrogate marker of microbial translocation, is associated with physical function in healthy older adults**. *Journals of Gerontology Series A-Biological Sciences & Medical Sciences* 2012, **67**(11):1212-1218.

208. Gomez JF, Curcio CL, Alvarado B, Zunzunegui MV, Guralnik J: **Validity and reliability of the Short Physical Performance Battery (SPPB): a pilot study on mobility in the Colombian Andes**. *Colombia Medica* 2013, **44**(3):165-171.

209. Lana A, Struijk E, Guallar-Castillón P, Martín-Moreno JM, Rodríguez Artalejo F, Lopez-Garcia E: **Leptin concentration and risk of impaired physical function in older adults: the Seniors-ENRICA cohort**. *Age and Ageing* 2016, **45**(6):819-826.

210. Wilson C, Perkin OJ, McGuigan MP, Stokes KA: **The effect of age on technique variability and outcome variability during a leg press**. *PloS One* 2016, **11**(10):e0163764.

211. Ahmed T, Vafaei A, Auais M, Guralnik J, Zunzunegui MV: **Gender roles and physical function in older adults: Cross-sectional analysis of the International Mobility in Aging Study (IMIAS)**. *PloS One* 2016, **11**(6):e0156828.

212. Bollaert RE, Marsh AP, Cutter GR, Motl RW: **The virtual Short Physical Performance Battery: psychometric properties and validation in older adults with multiple sclerosis**. *Journal of Applied Gerontology* 2017:0733464817709532.

213. Holwerda AM, Overkamp M, Paulussen KJ, Smeets JS, van Kranenburg J, Backx EM, Gijsen AP, Goessens JP, Verdijk LB, van Loon LJ: **Protein supplementation after exercise and before sleep does not further augment muscle mass and strength gains during resistance exercise training in active older men**. *The Journal of Nutrition* 2018, **148**(11):1723-1732.

214. Auais M, French S, Alvarado B, Pirkle C, Belanger E, Guralnik J: **Fear of falling predicts incidence of functional disability 2 years later: A perspective from an international cohort study**. *The Journals of Gerontology: Series A* 2017, **73**(9):1212-1215.

215. Osawa Y, Studenski SA, Ferrucci L: **Knee extension rate of torque development and peak torque: associations with lower extremity function**. *Journal of Cachexia, Sarcopenia and Muscle* 2018.

216. Distefano G, Standley RA, Zhang X, Carnero EA, Yi F, Cornnell HH, Coen PM: **Physical activity unveils the relationship between mitochondrial energetics, muscle quality, and physical function in older adults**. *Journal of Cachexia, Sarcopenia and Muscle* 2018, **9**(2):279-294.

217. Cegielski J, Brook MS, Quinlan JI, Wilkinson DJ, Smith K, Atherton PJ, Phillips BE: **A 4-week, lifestyle-integrated, home-based exercise training programme elicits improvements in physical function and lean mass in older men and women: a pilot study**. *F1000Research* 2017, **6**.

218. Ćwirlej-Sozańska A, Wiśniowska-Szurlej A, Wilmowska-Pietruszyńska A, Sozański B, Wołoszyn N: **Assessment of psychophysical capacities for professional work in late middle age and at the beginning of old age**. *Medycyna Pracy* 2018, **69**(4):375-381.

219. Manckoundia P, Thomas F, Buatois S, Guize L, Jego B, Aquino JP, Benetos A: **Impact of clinical, psychological, and social factors on decreased Tinetti test score in community-living elderly subjects: a prospective study with two-year follow-up**. *Medical Science Monitor* 2008, **14**(6):CR316-322.

220. Aung KC, Feng L, Yap KB, Sitoh YY, Leong IY, Ng TP: **Serum albumin and hemoglobin are associated with physical function in community-living older persons in Singapore**. *Journal of Nutrition, Health & Aging* 2011, **15**(10):877-882.

221. Ng TP, Aung KC, Feng L, Feng L, Nyunt MS, Yap KB: **Tea consumption and physical function in older adults: a cross-sectional study**. *Journal of Nutrition, Health & Aging* 2014, **18**(2):161-166.

222. Ng TP, Aung KC, Feng L, Scherer SC, Yap KB: **Homocysteine, folate, vitamin B-12, and physical function in older adults: cross-sectional findings from the Singapore Longitudinal Ageing Study**. *American Journal of Clinical Nutrition* 2012, **96**(6):1362-1368.

223. Sobhani S, Sinaei E, Motealleh A, Hooshyar F, Kashkooli NS, Yoosefinejad AK: **Combined effects of whole body vibration and unstable shoes on balance measures in older adults: A randomized clinical trial**. *Archives of Gerontology and Geriatrics* 2018, **78**:30-37.

224. Schrack JA, Kuo P-L, Wanigatunga AA, Di J, Simonsick EM, Spira AP, Ferrucci L, Zipunnikov V: **Active-to-sedentary behavior transitions, fatigability, and physical functioning in older adults**. *The Journals of Gerontology: Series A* 2018.

225. Martinez-Amezcua P, Matsushita K, Simonsick EM, Ferrucci L, Schrack JA: **Fatigability and functional performance among older adults with low-normal ankle-brachial index: Cross-sectional findings from the Baltimore Longitudinal Study of Aging**. *Atherosclerosis* 2018, **272**:200-206.

226. Zhang W, Schwenk M, Mellone S, Paraschiv-Ionescu A, Vereijken B, Pijnappels M, Mikolaizak A, Boulton E, Jonkman N, Maier A: **Complexity of daily physical activity is more sensitive than conventional metrics to assess functional change in younger older adults**. *Sensors* 2018, **18**(7):2032.

227. Wang H, Wei A, Lu Y, Yu B, Chen W, Lu Y, Liu Y, Yu D, Zou L: **Simplified tai chi program training versus traditional tai chi on the functional movement screening in older adults**. *Evidence-based Complementary and Alternative Medicine* 2016, **2016**.

228. Coelho VA, Probst VS, Nogari BM, Teixeira DC, Felcar JM, Santos DC, Gomes MV, Andraus RA, Fernandes KB: **Angiotensin-II blockage, muscle strength, and exercise capacity in physically independent older adults**. *Journal of Physical Therapy Science* 2016, **28**(2):547-552.

229. Yaginuma Y, Abe T, Thiebaud RS, Kitamura T, Kawanishi M, Fukunaga T: **Can handgrip strength improve following body mass-based lower body exercise?** *BioResearch Open Access* 2017, **6**(1):19-27.

230. Scott D, Shore-Lorenti C, McMillan L, Mesinovic J, Clark RA, Hayes A, Sanders KM, Duque G, Ebeling PR: **Associations of components of sarcopenic obesity with bone health and balance in older adults**. *Archives of Gerontology and Geriatrics* 2018, **75**:125-131.

231. de Souza Barbosa JF, Zepeda MU, Beland F, Guralnik JM, Zunzunegui MV, Guerra RO: **Clinically relevant weakness in diverse populations of older adults participating in the International Mobility in Aging Study**. *Age* 2016, **38**(1):25.

232. Francis P, Mc Cormack W, Toomey C, Norton C, Saunders J, Kerin E, Lyons M, Jakeman P: **Twelve weeks’ progressive resistance training combined with protein supplementation beyond habitual intakes increases upper leg lean tissue mass, muscle strength and extended gait speed in healthy older women**. *Biogerontology* 2017, **18**(6):881-891.

233. Garcia IFF, Tiuganji CT, Simões MdSMP, Santoro IL, Lunardi AC: **Systemic effects of chronic obstructive pulmonary disease in young-old adults’ life-space mobility**. *International Journal of Chronic Obstructive Pulmonary Disease* 2017, **12**:2777.

234. Muller AM, Khoo S, Morris T: **Text messaging for exercise promotion in older adults from an upper-middle-income country: randomized controlled trial**. *Journal of Medical Internet Research* 2016, **18**(1):e5.

235. Hwang HJ, Kim SH: **The association among three aspects of physical fitness and metabolic syndrome in a Korean elderly population**. *Diabetology & metabolic syndrome* 2015, **7**:112.

236. Dip RM, Cabrera MA, Prato SF: **Association between body composition and stair negotiation ability among individuals> 55 years of age: a cross-sectional study**. *Clinical Interventions in Aging* 2017, **12**:1289.

237. Wanderley FA, Silva G, Marques E, Oliveira J, Mota J, Carvalho J: **Associations between objectively assessed physical activity levels and fitness and self-reported health-related quality of life in community-dwelling older adults**. *Quality of Life Research* 2011, **20**(9):1371-1378.

238. Gonzales JU, Defferari E, Fisher A, Shephard J, Proctor DN: **Calf exercise-induced vasodilation is blunted in healthy older adults with increased walking performance fatigue**. *Experimental Gerontology* 2014, **57**:1-5.

239. Aribisala BS, Gow AJ, Bastin ME, del Carmen Valdes Hernandez M, Murray C, Royle NA, Munoz Maniega S, Starr JM, Deary IJ, Wardlaw JM: **Associations between level and change in physical function and brain volumes**. *PLoS ONE [Electronic Resource]* 2013, **8**(11):e80386.

240. Zeng P, Han Y, Pang J, Wu S, Gong H, Zhu J, Li J, Zhang T: **Sarcopenia-related features and factors associated with lower muscle strength and physical performance in older Chinese: a cross sectional study**. *BMC Geriatrics* 2016, **16**:45.

241. Woo J, Leung J, Kwok T: **BMI, body composition, and physical functioning in older adults**. *Obesity* 2007, **15**(7):1886-1894.

242. Ismail N, Hairi F, Choo WY, Hairi NN, Peramalah D, Bulgiba A: **The Physical Activity Scale for the Elderly (PASE): Validity and reliability among community-dwelling older adults in Malaysia**. *Asia-Pacific Journal of Public Health* 2015, **27**(8 Suppl):62S-72S.

243. Antony B, Jones G, Stannus O, Blizzard L, Ding C: **Body fat predicts an increase and limb muscle strength predicts a decrease in leptin in older adults over 2.6 years**. *Clinical Endocrinology* 2013, **79**(5):652-660.

244. Hashemi R, Shafiee G, Motlagh AD, Pasalar P, Esmailzadeh A, Siassi F, Larijani B, Heshmat R: **Sarcopenia and its associated factors in Iranian older individuals: Results of SARIR study**. *Archives of Gerontology & Geriatrics* 2016, **66**:18-22.

245. Scott D, Hayes A, Sanders KM, Aitken D, Ebeling PR, Jones G: **Operational definitions of sarcopenia and their associations with 5-year changes in falls risk in community-dwelling middle-aged and older adults**. *Osteoporosis International* 2014, **25**(1):187-193.

246. Goldman N, Glei DA, Rosero-Bixby L, Chiou ST, Weinstein M: **Performance-based measures of physical function as mortality predictors: Incremental value beyond self-reports**. *Demographic Research [electronic resource]* 2014, **30**(7):227-252.

247. Leite JC, Forte R, de Vito G, Boreham CA, Gibney MJ, Brennan L, Gibney ER: **Comparison of the effect of multicomponent and resistance training programs on metabolic health parameters in the elderly**. *Archives of Gerontology & Geriatrics* 2015, **60**(3):412-417.

248. Hong GR, Cho SH, Tak Y: **Falls among Koreans 45 years of age and older: incidence and risk factors**. *Journal of Advanced Nursing* 2010, **66**(9):2014-2024.

249. Hamer M, Molloy GJ: **Association of C-reactive protein and muscle strength in the English Longitudinal Study of Ageing**. *Age* 2009, **31**(3):171-177.

250. Wong CH, Wong SF, Pang WS, Azizah MY, Dass MJ: **Habitual walking and its correlation to better physical function: implications for prevention of physical disability in older persons**. *Journals of Gerontology Series A-Biological Sciences & Medical Sciences* 2003, **58**(6):555-560.

251. Wong CH, Wong SF, Yusoff AM, Karunananthan S, Bergman H: **The effect of later-life health promotion on functional performance and body composition**. *Aging-Clinical & Experimental Research* 2008, **20**(5):454-460.

252. Shah KN, Lin FV, Yu F, McMahon JM: **Activity engagement and physical function in old age sample**. *Archives of Gerontology and Geriatrics* 2017, **69**:55-60.

253. Guedes RC, Dias RC, Pereira LS, Silva SL, Lustosa LP, Dias JM: **Influence of dual task and frailty on gait parameters of older community-dwelling individuals**. *Brazilian Journal of Physical Therapy* 2014, **0**:0.

254. Evans M, Guthrie N, Pezzullo J, Sanli T, Fielding RA, Bellamine A: **Efficacy of a novel formulation of L-Carnitine, creatine, and leucine on lean body mass and functional muscle strength in healthy older adults: a randomized, double-blind placebo-controlled study**. *Nutrition & Metabolism* 2017, **14**(1):7.

255. Patel A, Edwards M, Jameson K, Ward K, Fuggle N, Cooper C, Dennison E: **Longitudinal change in peripheral quantitative computed tomography assessment in older adults: the hertfordshire cohort study**. *Calcified tissue international* 2018, **103**(5):476-482.

256. Arias-Fernández L, Machado-Fragua MD, Graciani A, Guallar-Castillón P, Banegas JR, Rodríguez-Artalejo F, Lana A, Lopez-Garcia E: **Prospective association between nut consumption and physical function in older men and women**. *The Journals of Gerontology: Series A* 2018.

257. Hurst C, Weston KL, Weston M: **The effect of 12 weeks of combined upper-and lower-body high-intensity interval training on muscular and cardiorespiratory fitness in older adults**. *Aging Clinical and Experimental Research* 2018:1-11.

258. McGrath RP, Vincent BM, Lee I-M, Kraemer WJ, Peterson MD: **Handgrip strength, function, and mortality in older adults: a time-varying approach**. *Medicine and science in sports and exercise* 2018, **50**(11):2259-2266.

259. Ryan A, Murphy C, Boland F, Galvin R, Smith SM: **What Is the impact of physical activity and physical function on the development of multimorbidity in older adults over time? A population-based cohort study**. *Journals of Gerontology: Series A Medical Sciences* 2018, **73**(11):1538-1544.

260. Yiengprugsawan V, Steptoe A: **Impacts of persistent general and site‐specific pain on activities of daily living and physical performance: A prospective analysis of the English Longitudinal Study of Ageing**. *Geriatrics & Gerontology International* 2018.

261. Trudelle-Jackson E, Jackson AW: **Do older adults who meet 2008 Physical Activity Guidelines have better physical performance than those who do not meet?** *Journal of Geriatric Physical Therapy* 2018, **41**(3):180-185.

262. Kramer JF, Vaz MD, Vandervoort AA: **Reliability of isometric hip abductor torques during examiner- and belt-resisted tests**. *Journal of Gerontology* 1991, **46**(2):M47-51.

263. Singh H, Kim D, Bemben M, Bemben D: **Relationship between muscle performance and DXA-derived bone parameters in community-dwelling older adults**. *Journal of Musculoskeletal & Neuronal Interactions* 2017, **17**(2):50.

264. Gronbech Jorgensen M, Andersen S, Ryg J, Masud T: **Novel use of the Nintendo Wii Board for measuring isometric lower limb strength: a reproducible and valid method in older adults**. *PLoS ONE* 2015, **10**(10):e0138660.

265. Abe T, Thiebaud RS, Loenneke JP, Mitsukawa N: **Association between toe grasping strength and accelerometer-determined physical activity in middle-aged and older women**. *Journal of Physical Therapy Science* 2015, **27**(6):1893-1897.

266. Assantachai P, Muangpaisan W, Intalapaporn S, Sitthichai K, Udompunturak S: **Cut-off points of quadriceps strength, declines and relationships of sarcopenia-related variables among Thai community-dwelling older adults**. *Geriatrics & gerontology international* 2014, **14 Suppl 1**:61-68.

267. Ahedi H, Aitken D, Scott D, Blizzard L, Cicuttini F, Jones G: **The association between hip muscle cross-sectional area, muscle strength, and bone mineral density**. *Calcified Tissue International* 2014, **95**(1):64-72.

268. Johnson LG, Butson ML, Polman RC, Raj IS, Borkoles E, Scott D, Aitken D, Jones G: **Light physical activity is positively associated with cognitive performance in older community dwelling adults**. *Journal of Science and Medicine in Sport* 2016, **19**(11):877-882.

269. Scott D, Blizzard L, Fell J, Ding C, Winzenberg T, Jones G: **A prospective study of the associations between 25‐hydroxy‐vitamin D, sarcopenia progression and physical activity in older adults**. *Clinical endocrinology* 2010, **73**(5):581-587.

270. Blomkvist AW, Andersen S, de Bruin E, Jorgensen MG: **Unilateral lower limb strength assessed using the Nintendo Wii Balance Board: a simple and reliable method**. *Aging Clinical and Experimental Research* 2017, **29**(5):1013-1020.

271. Gouveia ER, Maia JA, Beunen GP, Blimkie CJ, Fena EM, Freitas DL: **Functional fitness and physical activity of Portuguese community-residing older adults**. *Journal of Aging & Physical Activity* 2013, **21**(1):1-19.

272. Trapé AA, Lizzi EAdS, Gonçalves TCP, Rodrigues JAL, Tavares SS, Lacchini R, Pinheiro LC, Ferreira GC, Tanus-Santos JE, Ovídio PP: **Effect of multicomponent training on blood pressure, nitric oxide, redox status, and physical fitness in older adult women: influence of endothelial nitric oxide synthase (NOS3) haplotypes**. *Oxidative Medicine and Cellular Longevity* 2017, **2017**.

273. Hughes WE, Ueda K, Casey DP: **Chronic endurance exercise training offsets the age-related attenuation in contraction-induced rapid vasodilation**. *Journal of Applied Physiology* 2016:jap.00057.02016.

274. Borschmann K, Moore K, Russell M, Ledgerwood K, Renehan E, Lin X, Brown C, Sison J: **Overcoming barriers to physical activity among culturally and linguistically diverse older adults: a randomised controlled trial**. *Australasian Journal on Ageing* 2010, **29**(2):77-80.

275. Coker RH, Hays NP, Williams RH, Wolfe RR, Evans WJ: **Bed rest promotes reductions in walking speed, functional parameters, and aerobic fitness in older, healthy adults**. *Journals of Gerontology Series A-Biological Sciences & Medical Sciences* 2015, **70**(1):91-96.

276. Goldberg A, Chavis M, Watkins J, Wilson T: **The five-times-sit-to-stand test: validity, reliability and detectable change in older females**. *Aging-Clinical & Experimental Research* 2012, **24**(4):339-344.

277. Judd DL, Thomas AC, Dayton MR, Stevens-Lapsley JE: **Strength and functional deficits in individuals with hip osteoarthritis compared to healthy, older adults**. *Disability & Rehabilitation* 2014, **36**(4):307-312.

278. McCarthy EK, Horvat MA, Holtsberg PA, Wisenbaker JM: **Repeated chair stands as a measure of lower limb strength in sexagenarian women**. *Journals of Gerontology Series A-Biological Sciences & Medical Sciences* 2004, **59**(11):1207-1212.

279. Ng SS, Kwong PW, Chau MS, Luk IC, Wan SS, Fong SS: **Effect of arm position and foot placement on the five times sit-to-stand test completion times of female adults older than 50 years of age**. *Journal of Physical Therapy Science* 2015, **27**(6):1755-1759.

280. Stijntjes M, Meskers CG, de Craen AJ, van Lummel RC, Rispens SM, Slagboom PE, Maier AB: **Effect of calendar age on physical performance: A comparison of standard clinical measures with instrumented measures in middle-aged to older adults**. *Gait & Posture* 2016, **45**:12-18.

281. Mikhael M, Orr R, Amsen F, Greene D, Singh MA: **Effect of standing posture during whole body vibration training on muscle morphology and function in older adults: a randomised controlled trial**. *BMC Geriatrics* 2010, **10**:74.

282. Gluchowski A, Dulson D, Merien F, Plank L, Harris N: **Comparing the effects of two distinct eccentric modalities to traditional resistance training in resistance trained, higher functioning older adults**. *Experimental Gerontology* 2017, **98**:224-229.

283. Dobbs TJ, Simonson SR, Conger SA: **Improving power output in older adults using plyometrics in a body mass–supported treadmill**. *The Journal of Strength & Conditioning Research* 2018, **32**(9):2458-2465.

284. Kuo YL: **The influence of chair seat height on the performance of community-dwelling older adults' 30-second chair stand test**. *Aging-Clinical & Experimental Research* 2013, **25**(3):305-309.

285. Kalapotharakos VI, Michalopoulos M, Tokmakidis SP, Godolias G, Gourgoulis V: **Effects of a heavy and a moderate resistance training on functional performance in older adults**. *Journal of Strength & Conditioning Research* 2005, **19**(3):652-657.

286. Naugle KM, Higgins TJ, Manini TM: **Obesity and use of compensatory strategies to perform common daily activities in pre-clinically disabled older adults**. *Archives of Gerontology & Geriatrics* 2012, **54**(2):e134-138.

287. Baer GD, Ashburn AM: **Trunk movements in older subjects during sit-to-stand**. *Archives of Physical Medicine & Rehabilitation* 1995, **76**(9):844-849.

288. Marques E, Carvalho J, Pizarro A, Wanderlay F, Mota J: **The influence of physical activity, body composition, and lower extremity strength on walking ability**. *Motor Control* 2011, **15**(4):494-506.

289. Brose A, Parise G, Tarnopolsky MA: **Creatine supplementation enhances isometric strength and body composition improvements following strength exercise training in older adults**. *Journals of Gerontology Series A-Biological Sciences & Medical Sciences* 2003, **58**(1):11-19.

290. Crockett K, Ardell K, Hermanson M, Penner A, Lanovaz J, Farthing J, Arnold C: **The relationship of knee-extensor strength and rate of torque development to sit-to-stand performance in older adults**. *Physiotherapy Canada* 2013, **65**(3):229-235.

291. Yasuda T, Fukumura K, Fukuda T, Uchida Y, Iida H, Meguro M, Sato Y, Yamasoba T, Nakajima T: **Muscle size and arterial stiffness after blood flow-restricted low-intensity resistance training in older adults**. *Scandinavian Journal of Medicine & Science in Sports* 2014, **24**(5):799-806.

292. King AC, Pruitt LA, Phillips W, Oka R, Rodenburg A, Haskell WL: **Comparative effects of two physical activity programs on measured and perceived physical functioning and other health-related quality of life outcomes in older adults**. *Journals of Gerontology Series A-Biological Sciences & Medical Sciences* 2000, **55**(2):M74-83.

293. Smith JW, Marcus RL, Tracy BL, Foreman KB, Christensen JC, LaStayo PC: **Stance time variability during stair stepping before and after total knee arthroplasty: A pilot study**. *Human Movement Science* 2016, **45**:53-62.

294. Vincent KR, Braith RW, Feldman RA, Magyari PM, Cutler RB, Persin SA, Lennon SL, Gabr AH, Lowenthal DT: **Resistance exercise and physical performance in adults aged 60 to 83**. *Journal of the American Geriatrics Society* 2002, **50**(6):1100-1107.
