# Supplementary material for: Performance-based clinical tests of balance and muscle strength used in young seniors: a systematic literature review
Source: BMC Geriatr. 2019 Jan 9;19:9. doi: 10.1186/s12877-018-1011-0 (PMC6327480; doi:10.1186/s12877-018-1011-0)
Supplement: Supplementary file 3 — The quality of studies assessing validity and/or reliability of included balance and strength tools and the rating of the reported results. Brief description: Overview of the identified methodological studies. (DOCX 28 kb) [file 12877_2018_1011_MOESM3_ESM.docx]

**Additional file 3**

**Additional file 3.** The quality of studies assessing validity and/or reliability of included balance and strength tools and the rating of the reported results

|  |  |  | **Validity** | | | | **Reliability** | | | | | | | |
| --- | --- | --- | --- | --- | --- | --- | --- | --- | --- | --- | --- | --- | --- | --- |
| **Assessment Tool** |  |  | Construct | | Criterion | | Internal consistency | | Inter-rater | | Intra-rater | | Test-retest | |
|  | ref | n | Quality  of study | Reported  result | Quality  of study | Reported  result | Quality  of study | Reported  result | Quality of study | Reported  result | Quality of study | Reported  result | Quality  of study | Reported  result |
| Tandem, 10s | [1] | 37 |  |  | F | β. 92  R^2^ .98 |  |  |  |  |  |  |  |  |
|  | [1] | 20 |  |  |  |  |  |  |  |  |  |  | F | ICC_2,1_ .82 |
| TUG | [2] | 12 |  |  |  |  |  |  |  |  |  |  | P | PCC .90-.97 |
|  | [3] | 10 |  |  |  |  |  |  | P | ICC_3,1_ .97 |  |  |  |  |
|  | [4] | 60 | G | RR 3.2 |  |  |  |  |  |  |  |  |  |  |
| SPPB | [5] | 150 | E | N/A |  |  |  |  |  |  |  |  | G | ICC .87 |
| CBM | [6] | 51 |  |  | F | SCC .32-.85 | P | *α* .998 | G | ICC_2,k_ 0.97 | G | ICC_3,k_ 1.00 |  |  |
|  | [7] | 25 | F | PCC .21-.63 |  |  |  |  |  |  |  |  |  |  |
| CBM (German) | [8] | 51 |  |  | F | SCC .32-.85 | G | *α* .998 | G | ICC_2,k_ .996 | G | ICC_3,k_ .998 |  |  |
| 5x STS | [9] | 12 |  |  |  |  |  |  |  |  | F | ICC .89 |  |  |

**Notes:** Timed Up-and-Go (TUG), Short Physical Performance Battery (SPPB), Community Balance & Mobility scale (CBM), Five times sit-to-stand (5x STS)**;** Quality of study (COSMIN): excellent (E), good (G), fair (F), poor (P); Cronbach’s alpha (*α*), beta Correlation coefficient (*β*), Coefficient of determination (R^2^), Risk Ratio (RR), Pearson Correlation Coefficient (PCC), Spearman Correlation Coefficient (SCC); Intraclass Correlation Coefficient (ICC), number (n), reference (ref), Not Applicable (N/A),

**References**

1. Scaglioni-Solano P, Aragón-Vargas LF: **Validity and reliability of the Nintendo Wii Balance Board to assess standing balance and sensory integration in highly functional older adults**. *International Journal of Rehabilitation Research* 2014, **37**(2):138-143.

2. Morris S, Morris ME, Iansek R: **Reliability of measurements obtained with the Timed “Up & Go” test in people with Parkinson disease**. *Physical Therapy* 2001, **81**(2):810-818.

3. Ng SS, Hui-Chan CW: **The timed up & go test: its reliability and association with lower-limb impairments and locomotor capacities in people with chronic stroke**. *Archives of physical medicine and rehabilitation* 2005, **86**(8):1641-1647.

4. Alexandre TS, Meira DM, Rico NC, Mizuta SK: **Accuracy of Timed Up and Go Test for screening risk of falls among community-dwelling elderly**. *Brazilian Journal of Physical Therapy* 2012, **16**(5):381-388.

5. Gomez JF, Curcio CL, Alvarado B, Zunzunegui MV, Guralnik J: **Validity and reliability of the Short Physical Performance Battery (SPPB): a pilot study on mobility in the Colombian Andes**. *Colombia Medica* 2013, **44**(3):165-171.

6. Weber M, Van Ancum J, Bergquist R, Taraldsen K, Gordt K, Mikolaizak AS, Nerz C, Pijnappels M, Jonkman NH, Maier AB: **Concurrent validity and reliability of the Community Balance and Mobility scale in young-older adults**. *BMC Geriatrics* 2018, **18**(1):156.

7. Takacs J, Garland SJ, Carpenter MG, Hunt MA: **Validity and reliability of the Community Balance and Mobility scale in individuals with knee osteoarthritis**. *Physical Therapy* 2014, **94**(6):866-874.

8. Gordt K, Mikolaizak AS, Nerz C, Barz C, Gerhardy T, Weber M, Becker C, Schwenk M: **German version of the Community Balance and Mobility Scale**. *Zeitschrift für Gerontologie und Geriatrie* 2018:1-9.

9. Cani KC, Silva IJCS, Karloh M, Gulart AA, Matte DL, Mayer AF: **Reliability of the five-repetition sit-to-stand test in patients with chronic obstructive pulmonary disease on domiciliary oxygen therapy**. *Physiotherapy Theory and Practice* 2018:1-7.
